# Supplementary material for: Repurposing statins and phenothiazines to treat chemoresistant neuroblastoma
Source: EMBO Mol Med. 2025 Dec 23;18(2):433–61. doi: 10.1038/s44321-025-00349-6 (PMC12905276; doi:10.1038/s44321-025-00349-6)
Supplement: Supplementary file 1 — Appendix [file 44321_2025_349_MOESM1_ESM.pdf]

Appendix – Table of Contents

Appendix Figures

Appendix Figure S1 2–3

Appendix Figure S2 4–5

Appendix Figure S3 6–7

Appendix Figure S4 8–9

Appendix Figure S5 10–11

Appendix Tables

Appendix Table S1 12

Appendix Table S2 13

Appendix Table S3 14

Appendix Table S4 15

Appendix Table S5 16

S1

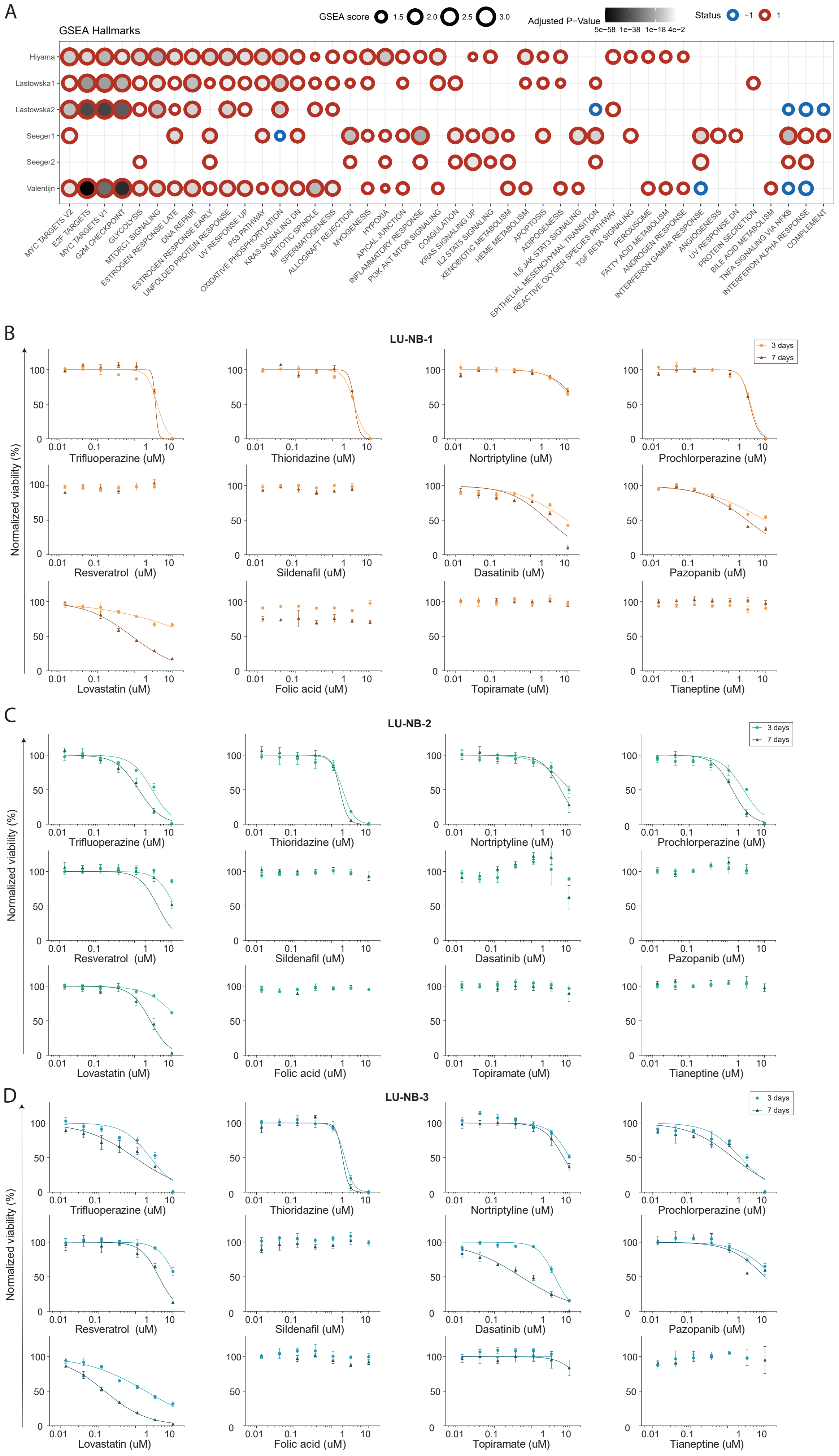
A
GSEA Hallmarks
GSEA score 1.5 2.0 2.5 3.0
Adjusted P−Value 5e−58 1e−38 1e−18 4e−2
Status −1 1
Hiyama
Lastowska1
Lastowska2
Seeger1
Seeger2
Valentijn
MYC TARGETS V2
E2F TARGETS
MYC TARGETS V1
G2M CHECKPOINT
GLYCOLYSIS
MTORC1 SIGNALING
ESTROGEN RESPONSE LATE
DNA REPAIR
ESTROGEN RESPONSE EARLY
UNFOLDED PROTEIN RESPONSE
UV RESPONSE UP
P53 PATHWAY
OXIDATIVE PHOSPHORYLATION
KRAS SIGNALING DN
MITOTIC SPINDLE
SPERMATOGENESIS
ALLOGRAFT REJECTION
MYOGENESIS
HYPOXIA
APICAL JUNCTION
INFLAMMATORY RESPONSE
PI3K AKT MTOR SIGNALING
COAGULATION
KRAS SIGNALING UP
IL2 STAT5 SIGNALING
XENOBIOTIC METABOLISM
HEME METABOLISM
APOPTOSIS
ADIPOGENESIS
IL6 JAK STAT3 SIGNALING
EPITHELIAL MESENCHYMAL TRANSITION
REACTIVE OXYGEN SPECIES PATHWAY
TGF BETA SIGNALING
PEROXISOME
FATTY ACID METABOLISM
ANDROGEN RESPONSE
INTERFERON GAMMA RESPONSE
ANGIOGENESIS
UV RESPONSE DN
PROTEIN SECRETION
BILE ACID METABOLISM
TNFA SIGNALING VIA NFKB
INTERFERON ALPHA RESPONSE
COMPLEMENT
B
LU-NB-1
3 days
7 days
Normalized viability (%)
Trifluoperazine (uM)
Thioridazine (uM)
Nortriptyline (uM)
Prochlorperazine (uM)
Resveratrol (uM)
Sildenafil (uM)
Dasatinib (uM)
Pazopanib (uM)
Lovastatin (uM)
Folic acid (uM)
Topiramate (uM)
Tianeptine (uM)
C
LU-NB-2
3 days
7 days
Normalized viability (%)
D
LU-NB-3
3 days
7 days
Normalized viability (%)

Appendix Figure S1. Gene expression profile (GEP) analysis and *in vitro* response of predicted drugs. A Gene set enrichment analysis (Hallmarks database) of GEPs across datasets included in the original drug predictions (Table S1). **B-D** Single dose-response curves for the predicted 12 drugs (Table 1). NB PDX-derived organoids (n=3) treated for 3 or 7 days: LU-NB-1, LU-NB-2, and LU-NB-3; data represent mean ± SD.

S2

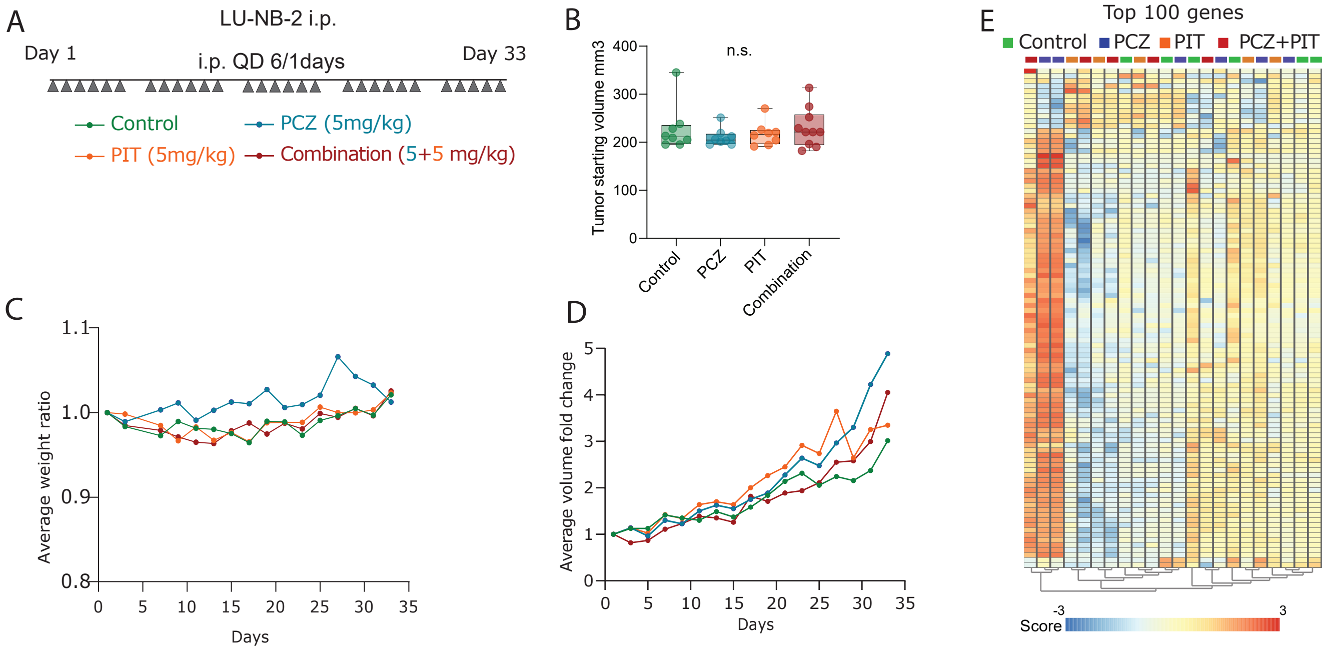
A
LU-NB-2 i.p.
Day 1
i.p. QD 6/1days
Day 33
Control
PCZ (5mg/kg)
PIT (5mg/kg)
Combination (5+5 mg/kg)
B
Tumor starting volume mm3
n.s.
Control
PCZ
PIT
Combination
C
Average weight ratio
Days
D
Average volume fold change
Days
E
Top 100 genes
Control
PCZ
PIT
PCZ+PIT
Score
-3
3

Appendix Figure S2. **Effects of intraperitoneal treatment in LU-NB-2**. **A** Schematic overview of the LU-NB-2 treatment study *in vivo*. Drugs administered intraperitoneally (i.p.) six times a week for 33 days. Control n=8; PCZ n=8; PIT n=8; Combination n=10 **B** Tumor starting volume (control n=8; PCZ n=8; PIT n=8; Combination n=10, one-way ANOVA followed by Tukey's multiple comparisons test, n.s. boxes represent the interquartile range and whiskers indicate minimum and maximum values). **C** Average weight ratio. **D** Average tumor size in each treatment group throughout the study duration. **E** Unsupervised gene expression analysis of the top 100 most variable genes from tumor samples collected on day 33. PCZ-prochlorperazine; PIT-pitavastatin.

S3

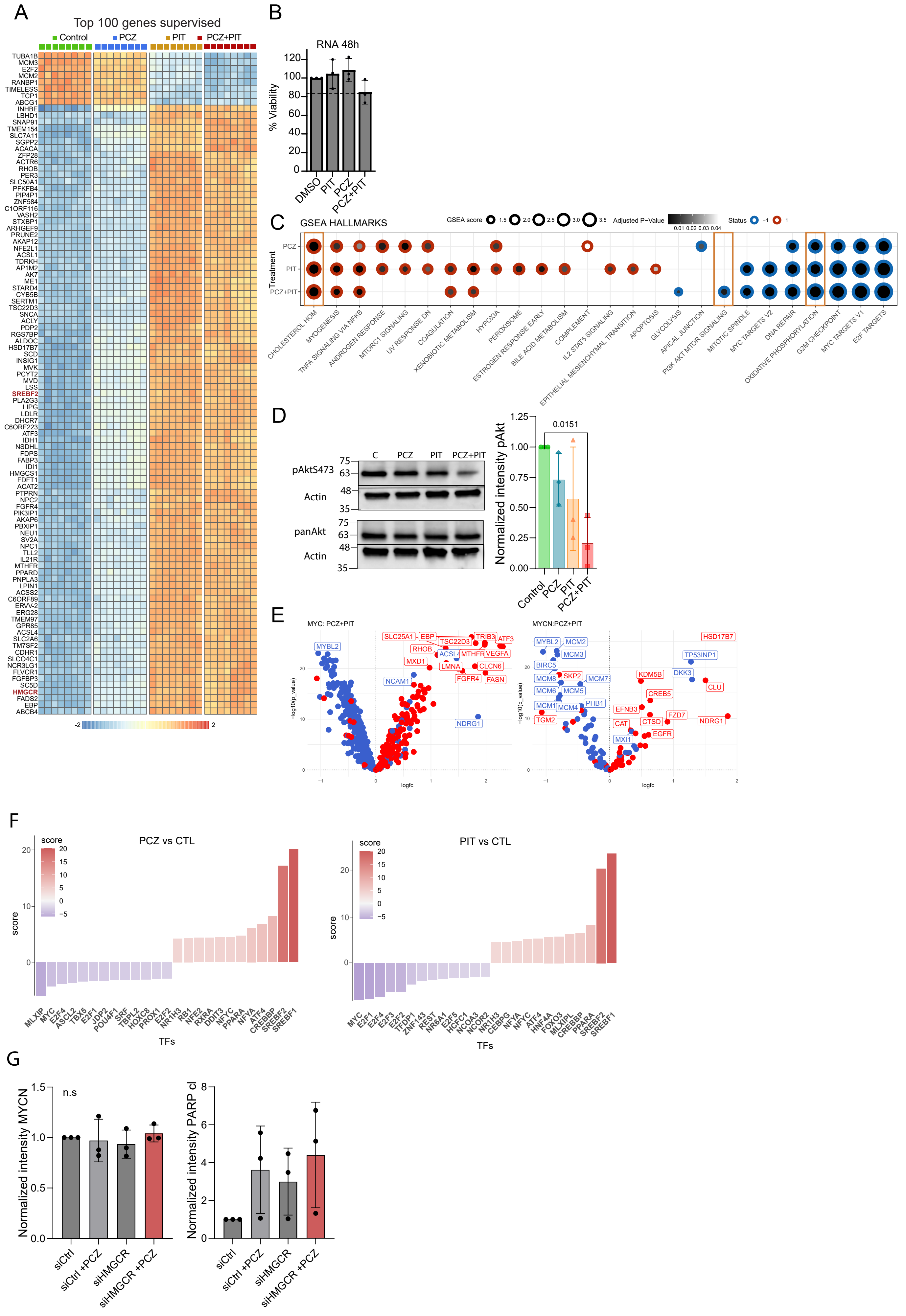

A
Top 100 genes supervised
Control
PCZ
PIT
PCZ+PIT
TUBA1B
MCM3
E2F2
MCM2
RANBP1
TIMELESS
TCP1
ABCG1
INHBE
LBHD1
SNAP91
TMEM154
SLC7A11
SGPP2
ACACA
ZFP28
ACTR6
RHOB
PER3
SLC50A1
PFKFB4
PIP4P1
ZNF584
C1ORF116
VASH2
STXBP1
ARHGEF9
PRUNE2
AKAP12
NFE2L1
ACSL1
TDRKH
AP1M2
AK7
ME1
STARD4
CYB5B
SERTM1
TSC22D3
SNCA
ACLY
PDP2
RGS7BP
ALDOC
HSD17B7
SCD
INSIG1
MVK
PCYT2
MVD
LSS
SREBF2
PLA2G3
LIPG
LDLR
DHCR7
C6ORF223
ATF3
IDH1
NSDHL
FDPS
FABP3
IDI1
HMGCS1
FDFT1
ACAT2
PTPRN
NPC2
FGFR4
PIK3IP1
AKAP6
PBXIP1
NEU1
SV2A
NPC1
TLL2
IL21R
MTHFR
PPARD
PNPLA3
LPIN1
ACSS2
C6ORF89
ERVV-2
ERG28
TMEM97
GPR85
ACSL4
SLC2A6
TM7SF2
CDHR1
SLCO4C1
NCR3LG1
FLVCR1
FGFBP3
HMGCR
SC5D
FADS2
EBP
ABCB4
-2
2
B
RNA 48h
% Viability
120
100
80
60
40
20
0
DMSO
PIT
PCZ
PCZ+PIT
C
GSEA HALLMARKS
GSEA score
1.5
2.0
2.5
3.0
3.5
Adjusted P-Value
0.01 0.02 0.03 0.04
Status
-1
1
Treatment
PCZ
PIT
PCZ+PIT
CHOLESTEROL HOM
MYOGENESIS
TNFA SIGNALING VIA NFKB
ANDROGEN RESPONSE
MTORC1 SIGNALING
UV RESPONSE DN
COAGULATION
XENOBIOTIC METABOLISM
HYPOXIA
PEROXISOME
ESTROGEN RESPONSE EARLY
BILE ACID METABOLISM
COMPLEMENT
IL2 STAT5 SIGNALING
EPITHELIAL MESENCHYMAL TRANSITION
APOPTOSIS
GLYCOLYSIS
APICAL JUNCTION
PI3K AKT MTOR SIGNALING
MITOTIC SPINDLE
MYC TARGETS V2
DNA REPAIR
OXIDATIVE PHOSPHORYLATION
G2M CHECKPOINT
MYC TARGETS V1
E2F TARGETS
D
C
PCZ
PIT
PCZ+PIT
pAktS473
Actin
panAkt
Actin
75
63
48
35
75
63
48
35
Normalized intensity pAkt
1.25
1.00
0.75
0.50
0.25
0.00
0.0151
Control
PCZ
PIT
PCZ+PIT
E
MYC: PCZ+PIT
MYCN:PCZ+PIT
-log10(p_value)
logfc
MYBL2
SLC25A1
EBP
TSC22D3
TRIB3
ATF3
RHOB
ACSL4
MTHFR
VEGFA
MXD1
LMNA
CLCN6
NCAM1
FGFR4
FASN
NDRG1
MCM2
MCM3
HSD17B7
TP53INP1
BIRC5
DKK3
SKP2
KDM5B
MCM8
MCM7
CLU
MCM6
MCM5
CREB5
PHB1
MCM1
MCM4
EFNB3
FZD7
TGM2
CAT
CTSD
MXI1
EGFR
F
PCZ vs CTL
PIT vs CTL
score
TFs
MLXIP
MYC
E2F4
ASCL2
TBX5
E2F1
JDP2
POU4F1
SRF
TBPL2
HOXC8
PROX1
E2F2
NR1H3
RB1
NFE2
RXRA
DDIT3
NFYC
PPARA
NFYA
ATF4
CREBBP
SREBF2
SREBF1
MYC
E2F1
E2F4
E2F3
E2F2
TFDP1
ZNF143
REST
NR6A1
E2F5
HCFC1
NCOA3
NCOR2
NR1H3
CEBPG
NFYA
NFYC
ATF4
HNF4A
FOXO3
MLXIPL
CREBBP
PPARA
SREBF2
SREBF1
G
n.s
Normalized intensity MYCN
Normalized intensity PARP cl
siCtrl
siCtrl +PCZ
siHMGCR
siHMGCR +PCZ

Appendix Figure S3. **Mechanistic consequences of treatment with single drugs and PCZ + PIT combination. A** Heatmap showing differential gene expression between all treatment groups (top 100 most differentially expressed genes) of LU-NB-2 organoids after 48 h treatment with single drugs or PCZ+PIT combination. Corresponding cell viability of the RNAseq experiment shown in **B** (RNAseq: n=8 in each group, viability test: n=3; data represent mean ± SD). **C** GSEA (Hallmarks database) of the respective treatment groups vs. control. **D** Western blot analysis of pAkt-S473 and panAkt protein expression (n=3, one-way ANOVA followed by Tukey's multiple comparison test; data represent mean ± SD). **E** Volcano plots displaying expression of the downstream targets of *MYC*, and *MYCN* after PCZ+PIT combination treatment. **F** Transcription factor (TF) activity after treatment with single drugs (CollecTri) compared to control. **G** Western blot quantification of MYCN and PARP after siRNA treatment (n=3), one-way ANOVA; data represent mean ± SD). PCZ-prochlorperazine; PIT-pitavastatin.

S4

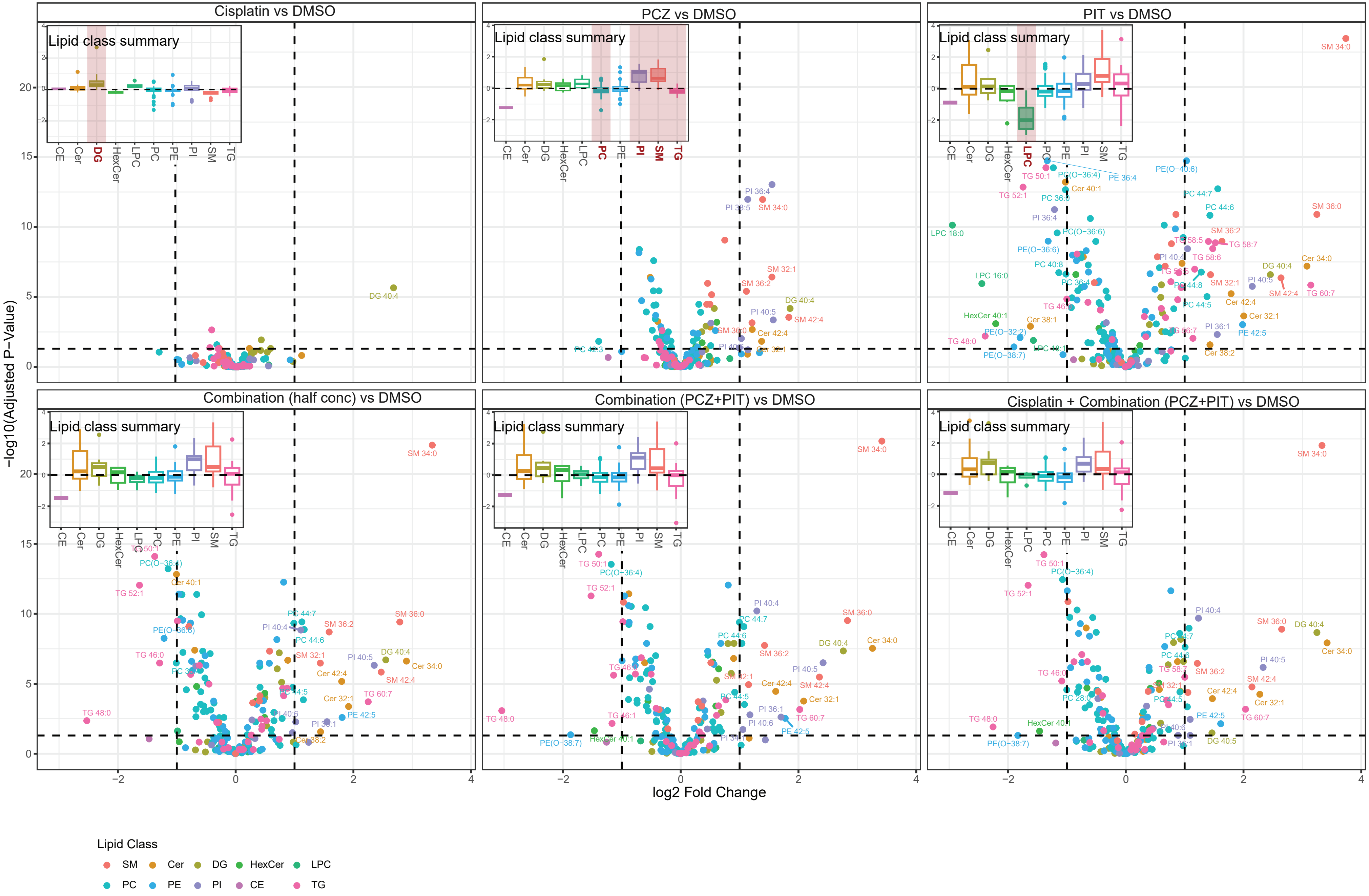

Cisplatin vs DMSO
PCZ vs DMSO
PIT vs DMSO
Combination (half conc) vs DMSO
Combination (PCZ+PIT) vs DMSO
Cisplatin + Combination (PCZ+PIT) vs DMSO
Lipid class summary
CE
Cer
DG
HexCer
LPC
PC
PE
PI
SM
TG
−log10(Adjusted P−Value)
log2 Fold Change
Lipid Class
SM
Cer
DG
HexCer
LPC
PC
PE
PI
CE
TG

Appendix Figure S4. Lipidomics changes after single drugs, combination, and combination + cisplatin treatment *in vitro*. Volcano plot presenting upregulated and downregulated lipids (species level nomenclature) after treatment with cisplatin, PCZ, PIT, PCZ+PIT at halved and full concentrations and cisplatin+PCZ+PIT. Upper left corner graphs summarize logFC of each lipid class change after treatment with significant changes indicated with the red background (boxes represent the interquartile range (IQR), center lines indicate medians, whiskers extend to 1.5×IQR, and points denote outliers). DMSO treated cells were used as a baseline. (n=6, p-values calculated by permutation test, corrected with Benjamini-Hochberg test. PCZ-prochlorperazine; PIT-pitavastatin.

S5

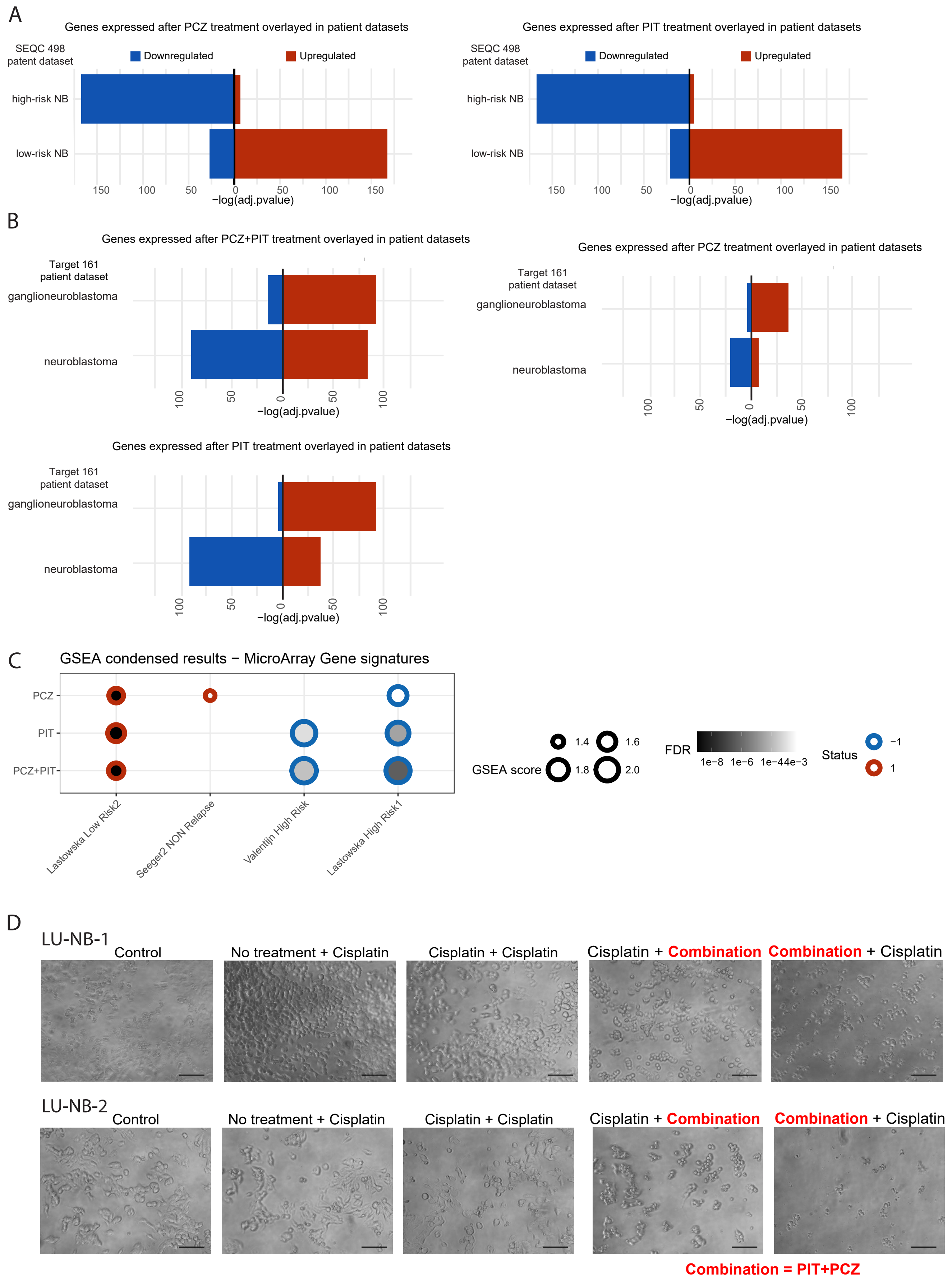
A
Genes expressed after PCZ treatment overlayed in patient datasets
SEQC 498
patent dataset
Downregulated
Upregulated
high-risk NB
low-risk NB
150
100
50
0
50
100
150
−log(adj.pvalue)
Genes expressed after PIT treatment overlayed in patient datasets
B
Genes expressed after PCZ+PIT treatment overlayed in patient datasets
Target 161
patient dataset
ganglioneuroblastoma
neuroblastoma
Genes expressed after PCZ treatment overlayed in patient datasets
Genes expressed after PIT treatment overlayed in patient datasets
C
GSEA condensed results − MicroArray Gene signatures
PCZ
PIT
PCZ+PIT
Lastowska Low Risk2
Seeger2 NON Relapse
Valentijn High Risk
Lastowska High Risk1
GSEA score
1.4
1.6
1.8
2.0
FDR
1e−8
1e−6
1e−44e−3
Status
−1
1
D
LU-NB-1
Control
No treatment + Cisplatin
Cisplatin + Cisplatin
Cisplatin + Combination
Combination + Cisplatin
LU-NB-2
Combination = PIT+PCZ

Appendix Figure S5. Transcriptional phenotype correlates with risk in patient datasets. **A** Association of DEGs (after single 1131 PCZ and PIT treatment) with risk stratification in the SEQC498 patient dataset. **B** Genes expressed after PCZ and/or PIT treatment overlayed in the Target161 dataset including patients with ganglioneuroblastoma and patients with aggressive neuroblastoma. **C** GSEA of RNA expression after treatment with single drugs and combination (LU-NB-2, 48 h) compared with risk in original datasets (Table S1). Only significant (FDR p-value < 0.05) results are displayed. **D** Brightfield images of cells seeded on laminin at different treatment conditions. Corresponding pictures of normalized viability graphs in Fig 4E. Picture magnification: 10x, scale bar 100 µm. PCZ-prochlorperazine; PIT-pitavastatin.

Appendix Table S1. Neuroblastoma expression datasets used for drug predictions.

**Author**	**NCBI GEO**	**Reference name**	**Benign group**	**Progression/higher severity group**	**Patient group**	**Number of patients**
Valentijn et al. (2015) *(87)*	GSE73537	Valentijn	Alive	Dead	All stages	34
Ohtaki et al. (2010)*(88)*	GSE16237	Hiyama	Alive + stages 1/2/4s	Dead of disease + stages 3/4	All stages	51
Asgarzadeh et al.(2006) *(89)*	GSE3446 HG-U133A	Seeger 1	No relapse	Relapse/metastasis	*MYCN* non-amplified	117
Asgarzadeh et al.(2006) *(89)*	GSE3446 HG-U133B	Seeger 2	No relapse	Relapse/metastasis	*MYCN* non-amplified	117
Lastowska et al.(2007)*(90)*	GSE13136	Lastowska 1	Low stage, MYCN non-amplified	*MYCN* amplified, high stage, 1p del	All stages, 17q gain	30
Lastowska et al. (2007) *(90)*	GSE13136	Lastowska 2	Low stage, *MYCN* non-amplified	*MYCN* non-amplified, high stage, 11q del	All stages, 17q gain	30

Appendix Table S2. Neuroblastoma PDX models included in the study.

PDX	Sample type	SNP profile	Stage	Chemotherapy
LU-NB-1	Primary (adrenal gland)	*MYCN* amp, 1p del, 17q gain	IV	No
LU-NB-2	Metastasis (after relapse)	*MYCN* amp, 1p del, 17q gain	IV	Yes
LU-NB-3	Primary (adrenal gland)	*MYCN* amp, 1p del, 17q gain	III	No

Appendix Table S3. **Effect of four selected drugs in three different high-risk neuroblastoma organoid models.** Single dose curves for statistical comparison between 3 and 7 days. NA- not enough data points around IC_{50} to compute the CI.

	LU-NB-1		LU-NB-2		LU-NB-3	
	3 days	7 days	3 days	7 days	3 days	7 days
**Trifluoperazine**						
IC_{50} (µM)	4.01	3.57	2.95	1.31	2.50	0.96
95% CI IC_{50}	NA	NA	2.4 – 3.6	1.1 – 1.5	1.8 – 3.4	0.62 – 1.49
Sig 3 vs 7 days	no	no	yes	yes	yes	yes
AUC	495	542	427	240	408	304
95% CI AUC	486 – 504	521 – 563	390 – 63.4	211 – 268	376 – 441	265 – 342
Sig 3 vs 7 days	yes	yes	yes	yes	yes	yes
**Thioridazine**						
IC_{50}(µM)	3.78	3.81	1.99	1.67	2.25	1.99
95% CI IC_{50}	3.53 – 4.06	NA	1.70 – 2.33	NA	2.06 – 2.50	NA
Sig 3 vs 7 days	no	no	no	no	no	no
AUC	478	533	278	224	300	247

95% CI AUC	468 – 487	521 – 545	258 – 298	205 – 244	270 – 331	212 – 283
Sig 3 vs 7 days	yes	yes	yes	yes	no	no
**Prochlorperazine**						
IC_{50}(μM)	3.93	3.77	2.73	1.38	2.20	1.41
95% CI IC_{50}	NA	NA	2.08 – 3.50	1.24 – 1.53	1.48 – 3.16	0.87 – 2.23
Sig 3 and 7 days	no	no	yes	yes	no	no
AUC	498.2	488.2	409.6	232.4	393.0	333.4
95% CI AUC	486 – 510	477 – 499	388 – 432	199 – 266	363 – 423	318 – 349
Sig 3 vs 7 days	no	no	no	no	no	no
**Lovastatin**						
IC_{50}(μM)	45.9	0.84	15.9	2.67	1.89	0.15
95% CI IC_{50}	24.0 – 119	0.73 – 0.97	13.0 – 21.4	2.30 – 3.09	1.54 – 2.33	0.14 – 0.17
Sig 3 vs 7 days	yes	yes	yes	yes	yes	yes
AUC	700	303	796	398	427	106
95% CI AUC	670 – 730	290 – 315	780 – 811	343 – 453	396 – 458	97.2 – 115
Sig 3 vs 7 days	yes	yes	yes	yes	yes	yes

Appendix Table S4. Neuroblastoma gene signatures included in the analysis.

Author	Material	States	Omics method
Van Groningen *et al.* 2017*(12)*	Cell lines (N=30)<br>Adr-mes cell line pairs (N=4)	Adrenergic<br>Mesenchymal	Chip-Seq, RNA-seq,
Boeva *et al.* 2017*(13)*	Patient tumors (N=10)<br>NB cell lines (N=25)<br>PDXs (N=6)	Noradrenergic<br>NCC-like	Chip-Seq, RNA-seq,
Gartlgruber *et al.* 2021*(16)*	Patient tumors (N=60)<br>Cell lines (N=25)	*MYCN* amp<br>*MYCN* non-amp, high risk<br>*MYCN* non-amp, low risk<br>MES/Schwann cell precursor	Chip-Seq (H3K27ac), RNAseq, ATAC-seq
Olsen *et al.* 2024*(18)*	Patient tumors (N=17)	Adrenergic<br>Mesenchymal<br>SCP-like<br>bridge cells	scRNA-seq
Manas *et al*. 2022*(19)*	Integration of signatures from PDX models (N=3) and multiple published signatures	Adrenergic<br>Mesenchymal-like	RNA-seq
Bedoya-Reina *et al.* 2021*(15)*	Patient tumors (N=11)<br>Normal adrenal gland (human+mouse) (N=3+5)	Undiff (nC2,3)<br>NOR (nC5,7,8,9)<br>Stromal clusters	sc/snRNA-seq
Yuan *et al.* 2022*(17)*	Patient tumors (N=10 peripheral neuroblastic tumors)	Adrenergic,<br>Transitional<br>Mesenchymal	scRNA-seq
Patel *et al.* bioRxiv*(22)*	Patient tumors (N=51)<br>PDX1(N=1)	Adrenergic<br>Mesenchymal<br>Sympathoblast	sc/snRNA-seq<br>Spatial transcriptomics

Appendix Table S5. Multispectral staining antibody-opal pairing.

Antibody name	Company and number	Dilution	Opal name/product number	Dilution
NCAM	DAKO NCL-L-CD56-504	1:50	Opal 780	1:100
SOX9	Abcam Ab76997	1:500	Opal 480	1:100
TH	Abcam Ab112	1:1000	Opal 620	1:100
CD44	Abcam Ab157107	1:500	Opal 520	1:100
PHOX2B	Abcam Ab183741	1:1000	Opal 570	1:100
