# Supplementary figures and images for: Repurposing statins and phenothiazines to treat chemoresistant neuroblastoma
Source: EMBO Mol Med. 2025 Dec 23;18(2):433–61. doi: 10.1038/s44321-025-00349-6 (PMC12905276; doi:10.1038/s44321-025-00349-6)

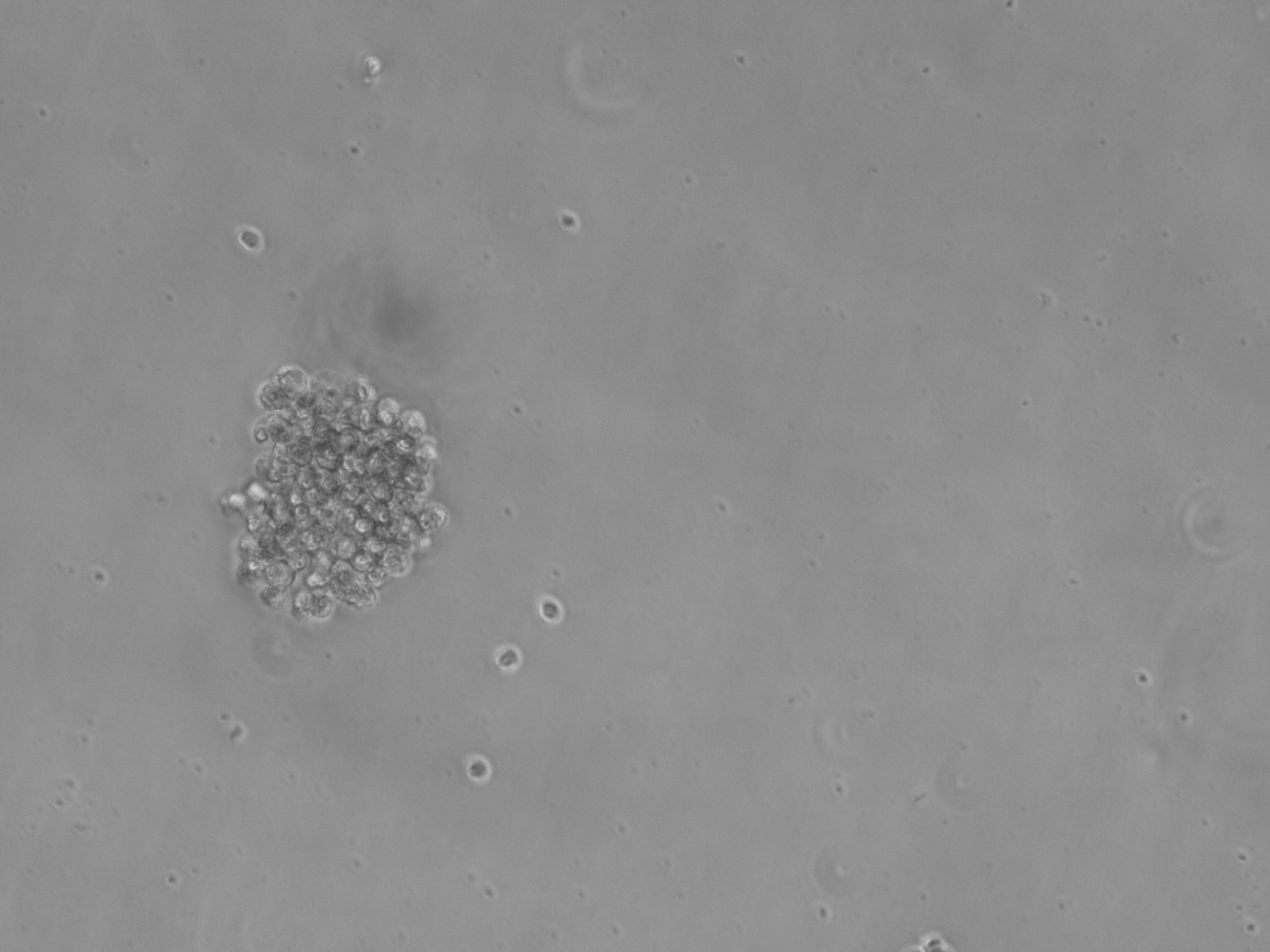

Supplement: Supplementary file 3 — Source data Fig. 2 [file 44321_2025_349_MOESM3_ESM.zip › Source data Figure 2/Fig2D/LUNB1/Combination (PIT+PCZ).tif]

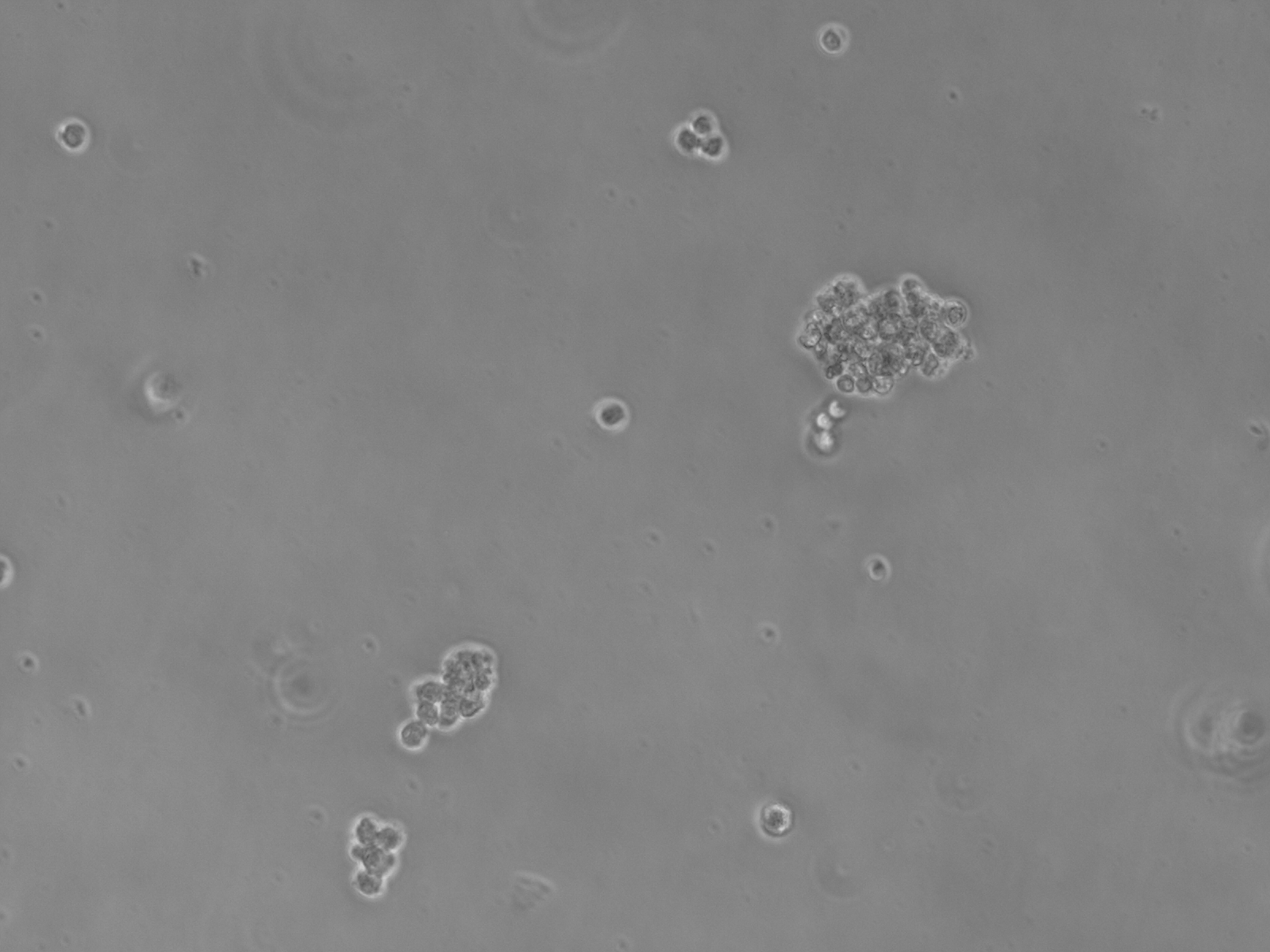

Supplement: Supplementary file 3 — Source data Fig. 2 [file 44321_2025_349_MOESM3_ESM.zip › Source data Figure 2/Fig2D/LUNB1/Combination (PIT+PCZ)_Fig2D.tif]

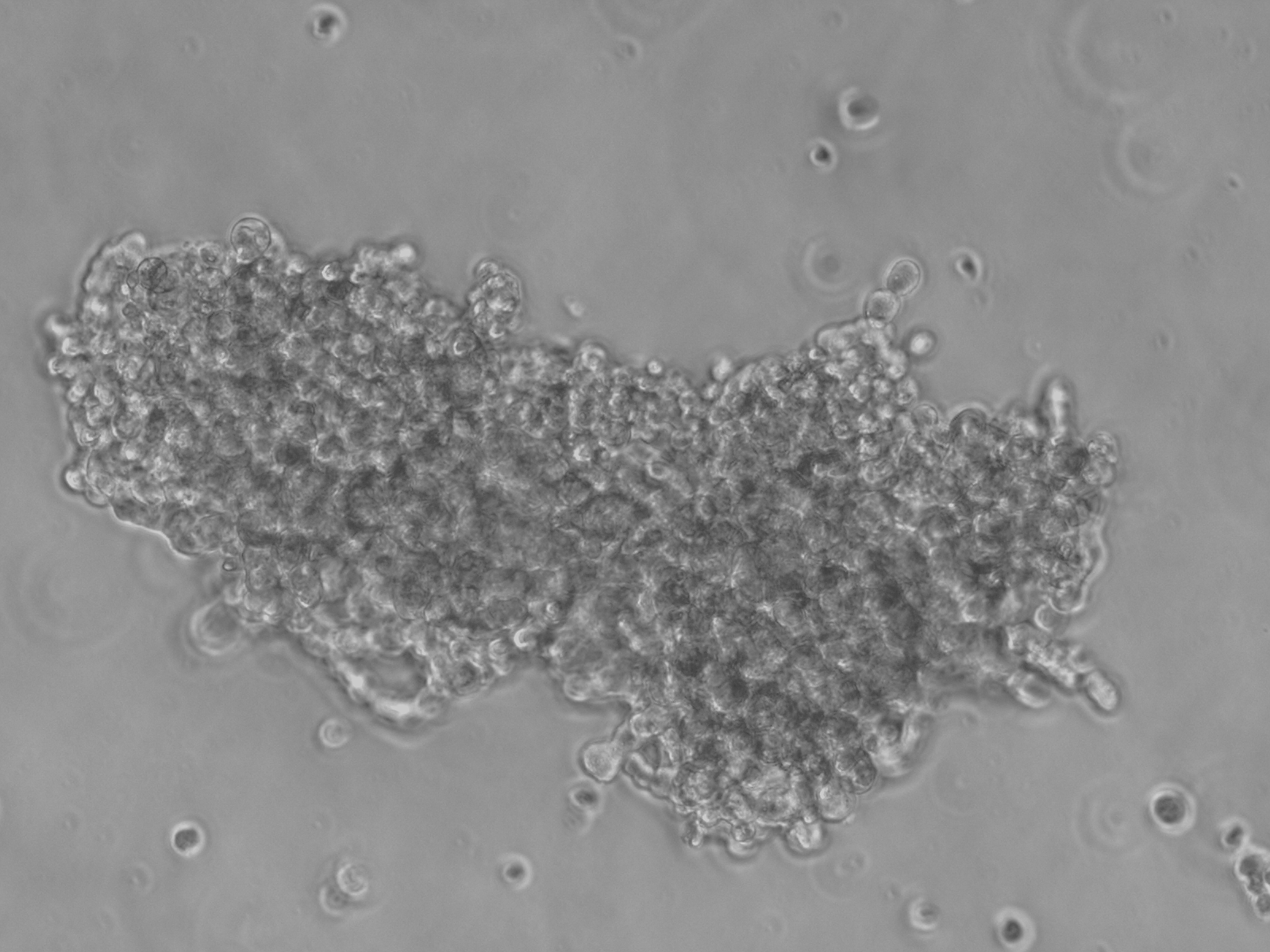

Supplement: Supplementary file 3 — Source data Fig. 2 [file 44321_2025_349_MOESM3_ESM.zip › Source data Figure 2/Fig2D/LUNB1/Control (DMSO).tif]

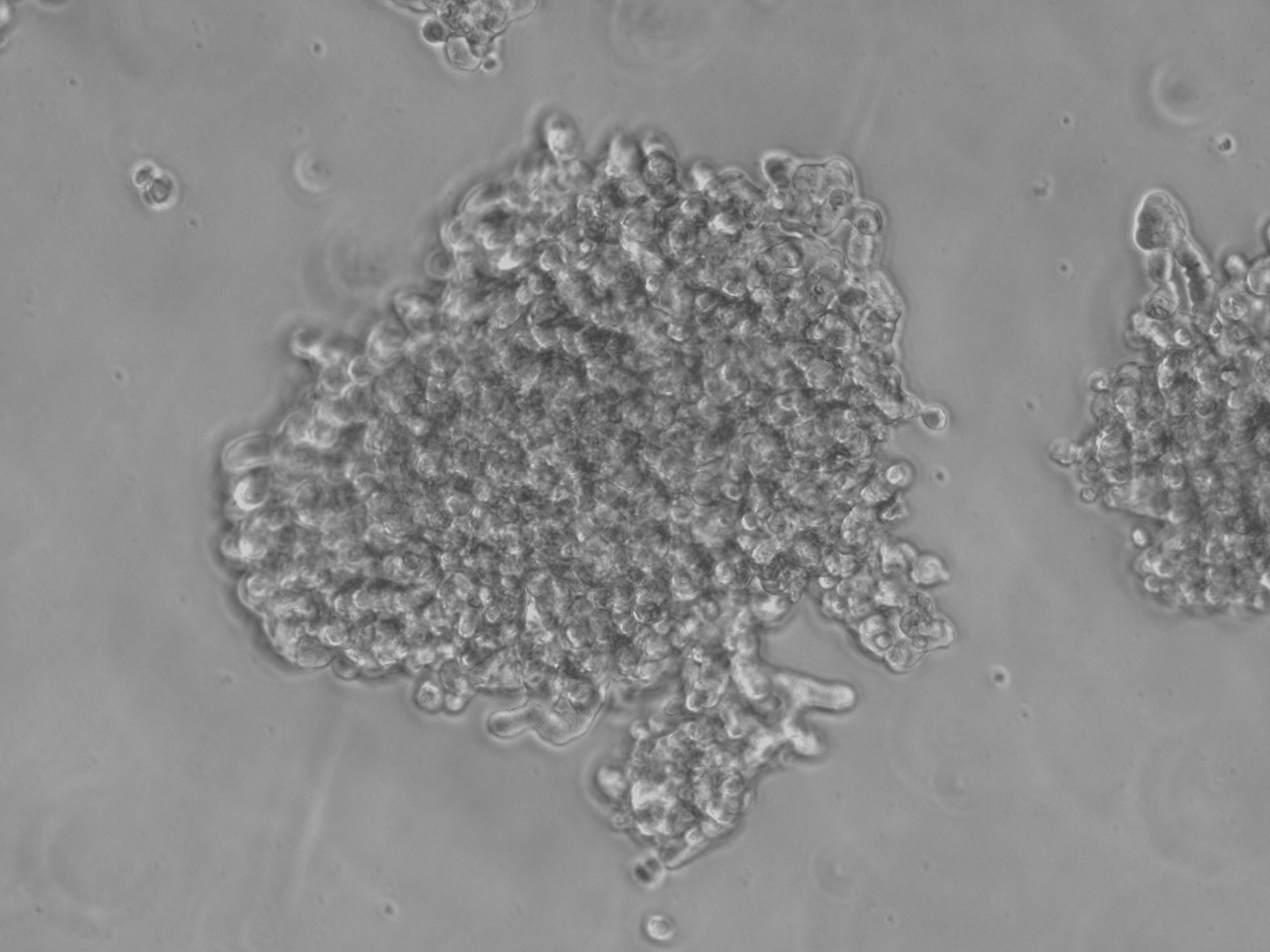

Supplement: Supplementary file 3 — Source data Fig. 2 [file 44321_2025_349_MOESM3_ESM.zip › Source data Figure 2/Fig2D/LUNB1/Control (DMSO)_Fig2D.tif]

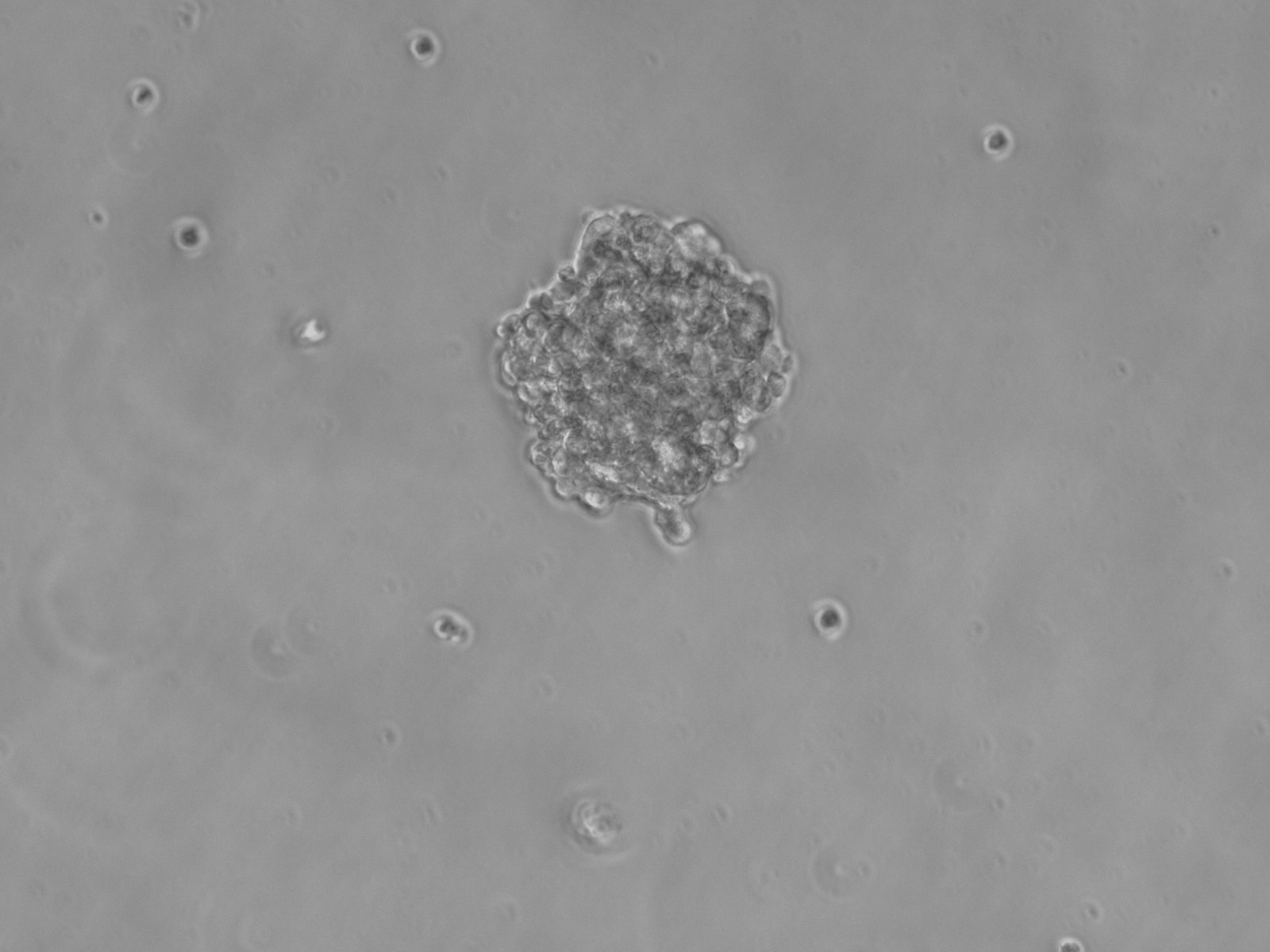

Supplement: Supplementary file 3 — Source data Fig. 2 [file 44321_2025_349_MOESM3_ESM.zip › Source data Figure 2/Fig2D/LUNB1/PCZ.tif]

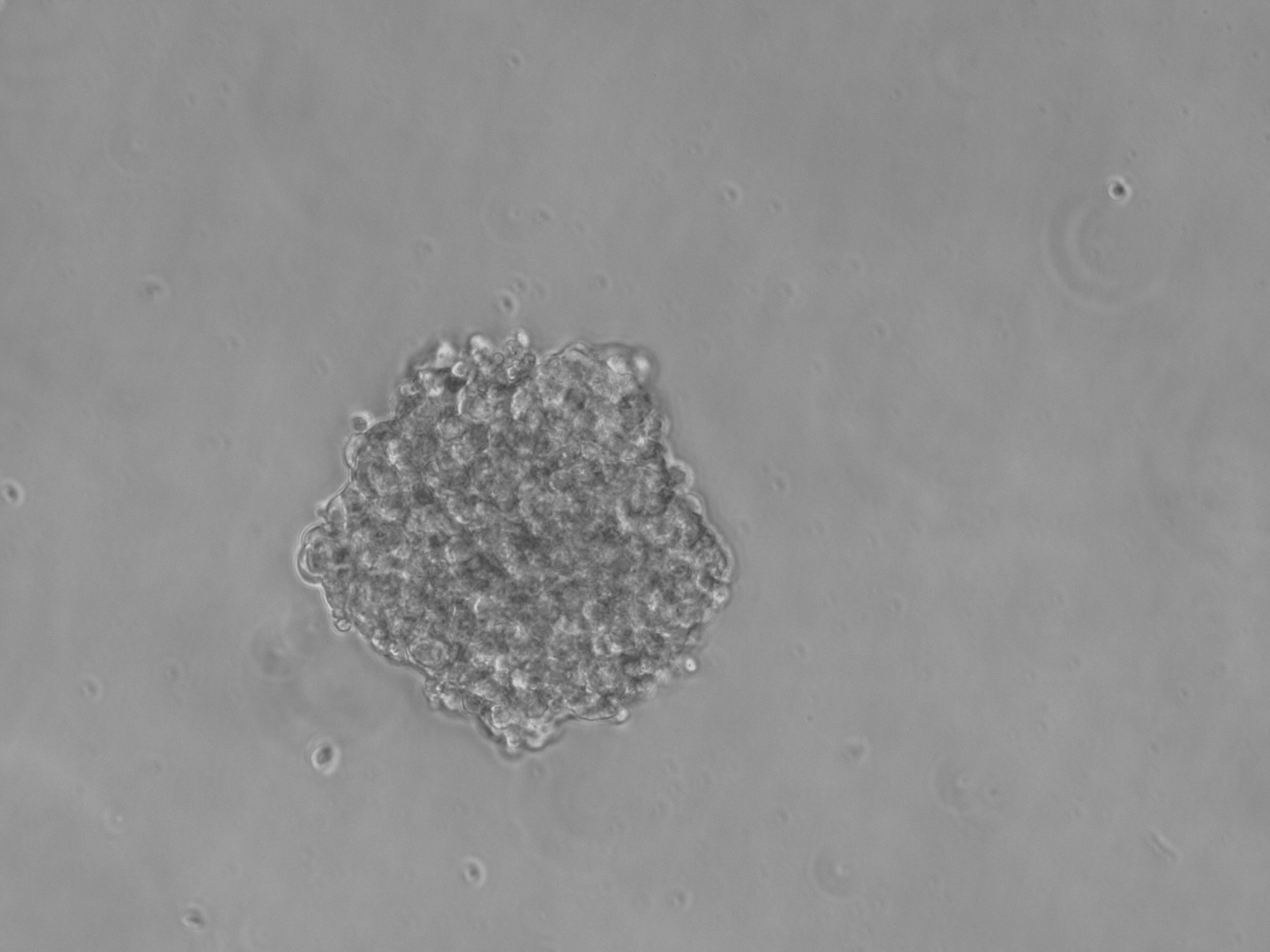

Supplement: Supplementary file 3 — Source data Fig. 2 [file 44321_2025_349_MOESM3_ESM.zip › Source data Figure 2/Fig2D/LUNB1/PCZ_Fig2D.tif]

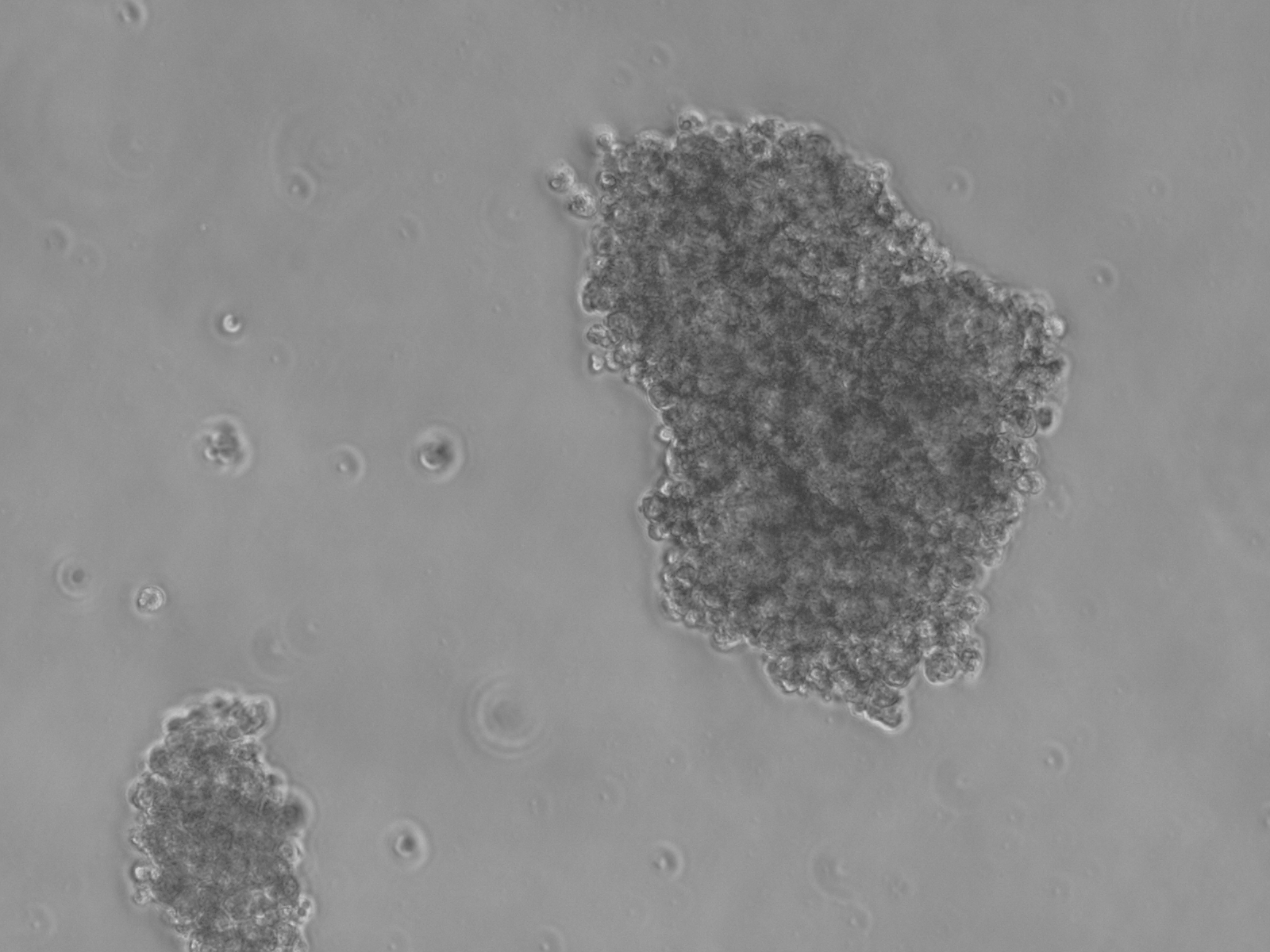

Supplement: Supplementary file 3 — Source data Fig. 2 [file 44321_2025_349_MOESM3_ESM.zip › Source data Figure 2/Fig2D/LUNB1/PIT.tif]

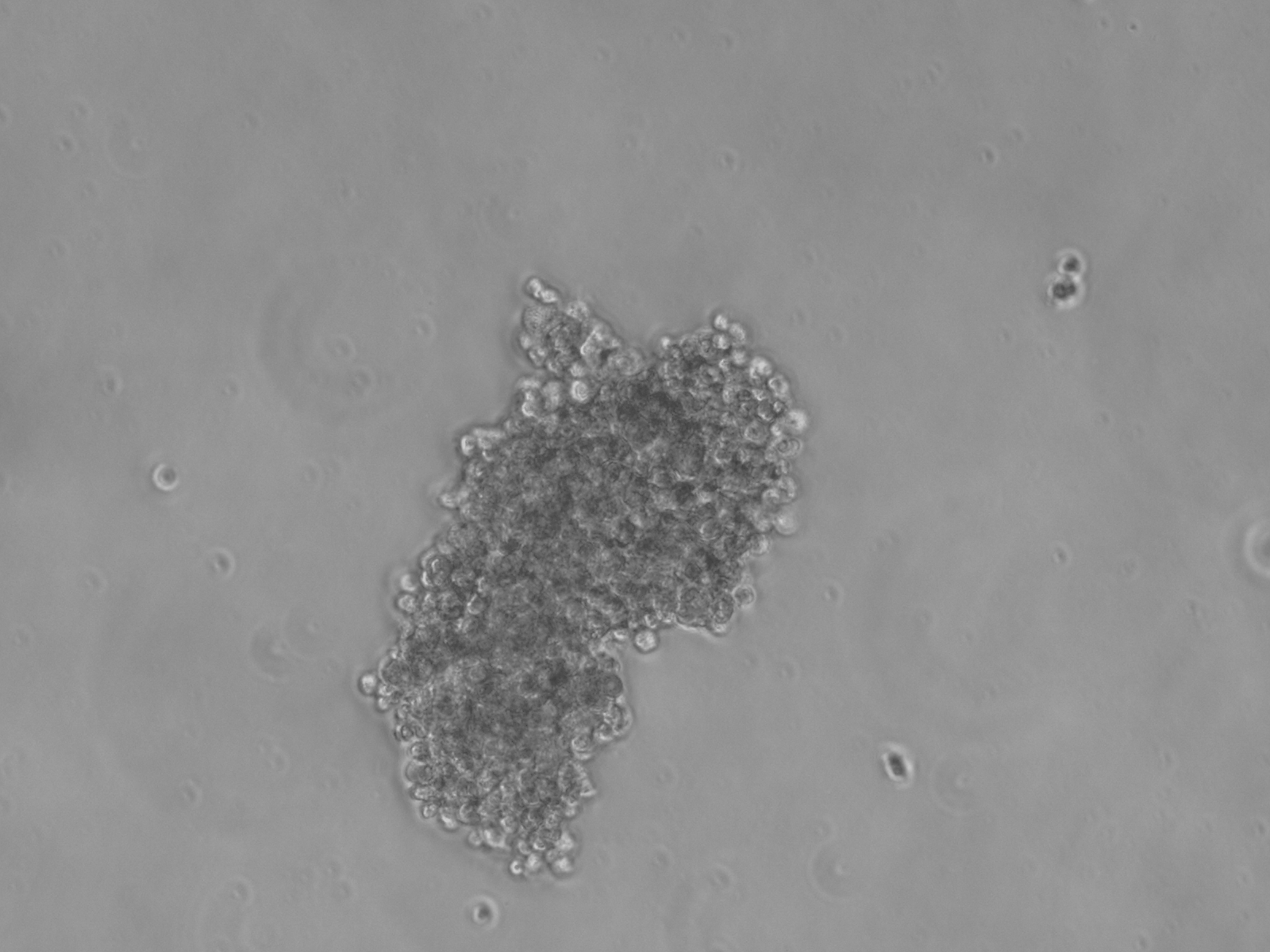

Supplement: Supplementary file 3 — Source data Fig. 2 [file 44321_2025_349_MOESM3_ESM.zip › Source data Figure 2/Fig2D/LUNB1/PIT_Fig2D.tif]

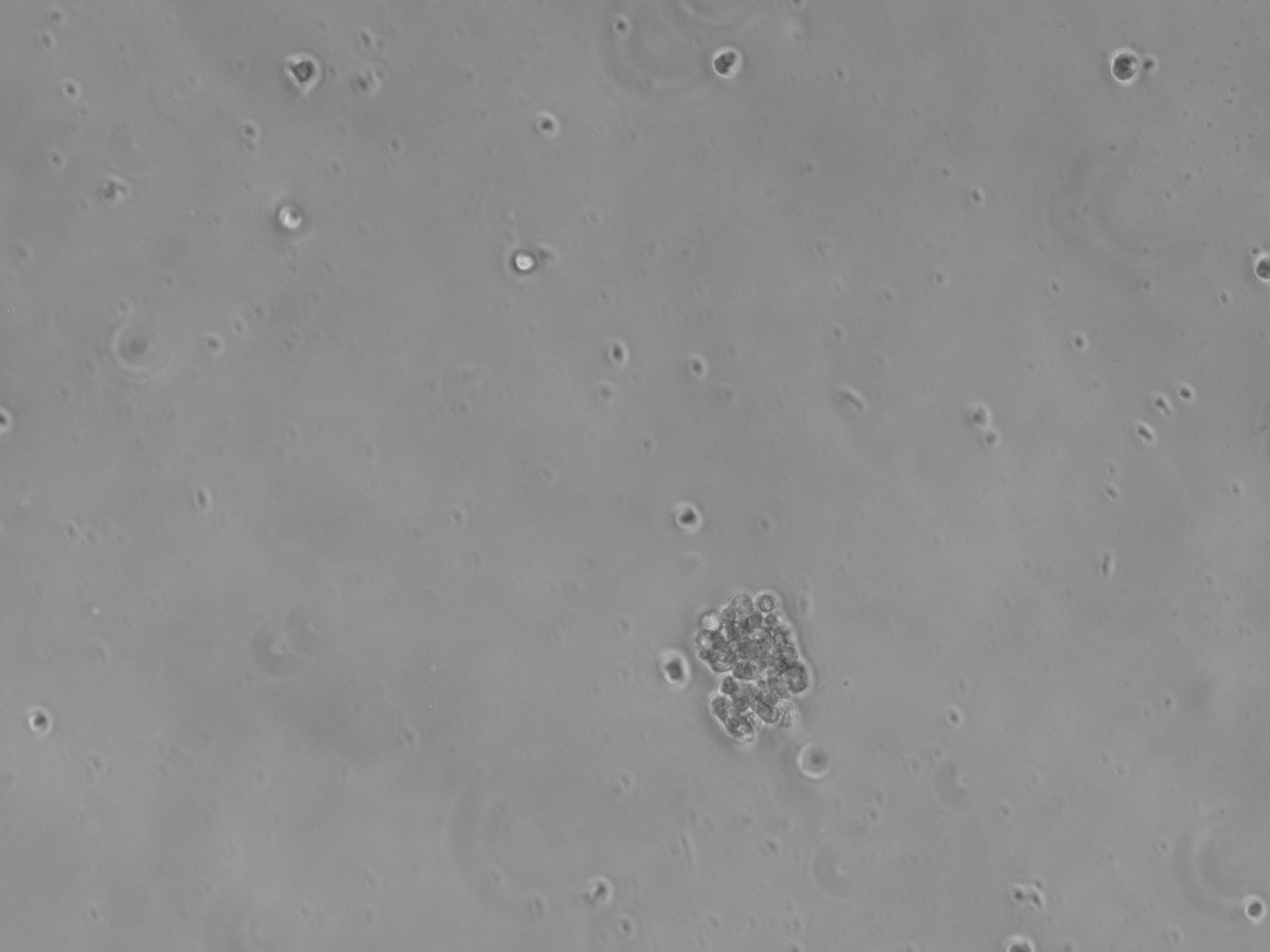

Supplement: Supplementary file 3 — Source data Fig. 2 [file 44321_2025_349_MOESM3_ESM.zip › Source data Figure 2/Fig2D/LUNB2/Combination (PIT+PCZ)_Fig2D.tif]

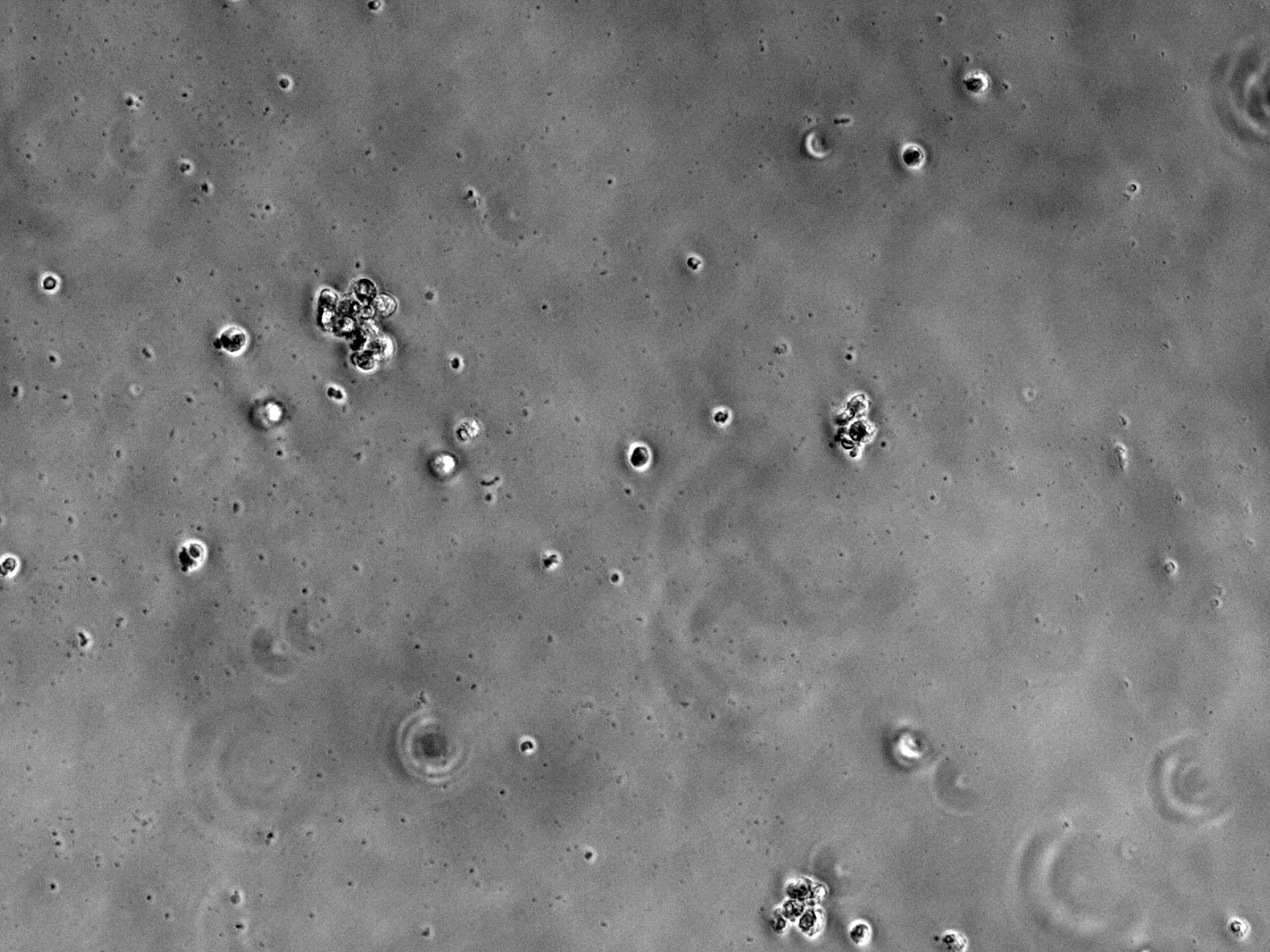

Supplement: Supplementary file 3 — Source data Fig. 2 [file 44321_2025_349_MOESM3_ESM.zip › Source data Figure 2/Fig2D/LUNB2/Combination(PIT+PCZ).tif]

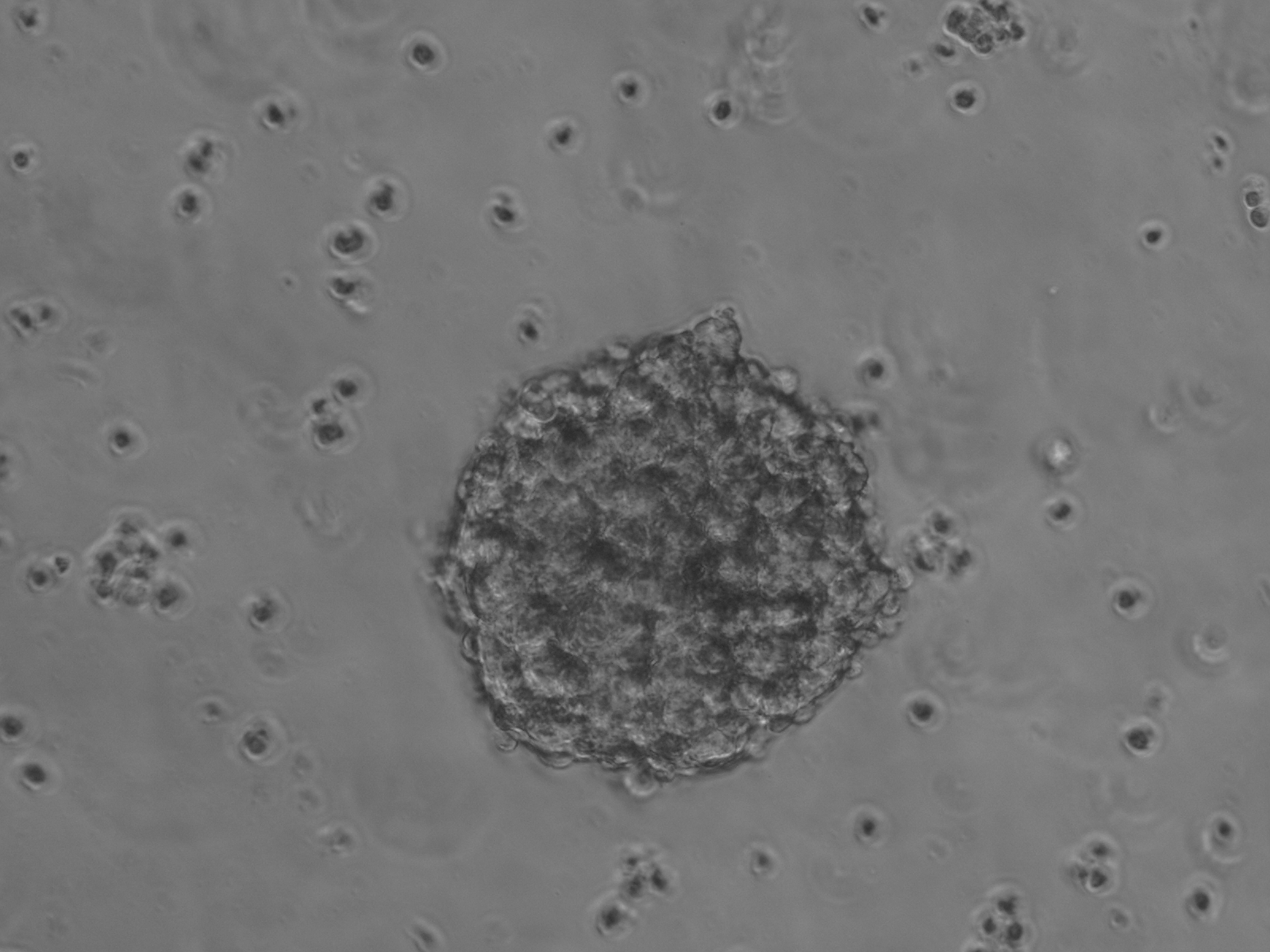

Supplement: Supplementary file 3 — Source data Fig. 2 [file 44321_2025_349_MOESM3_ESM.zip › Source data Figure 2/Fig2D/LUNB2/Control (DMSO).tif]

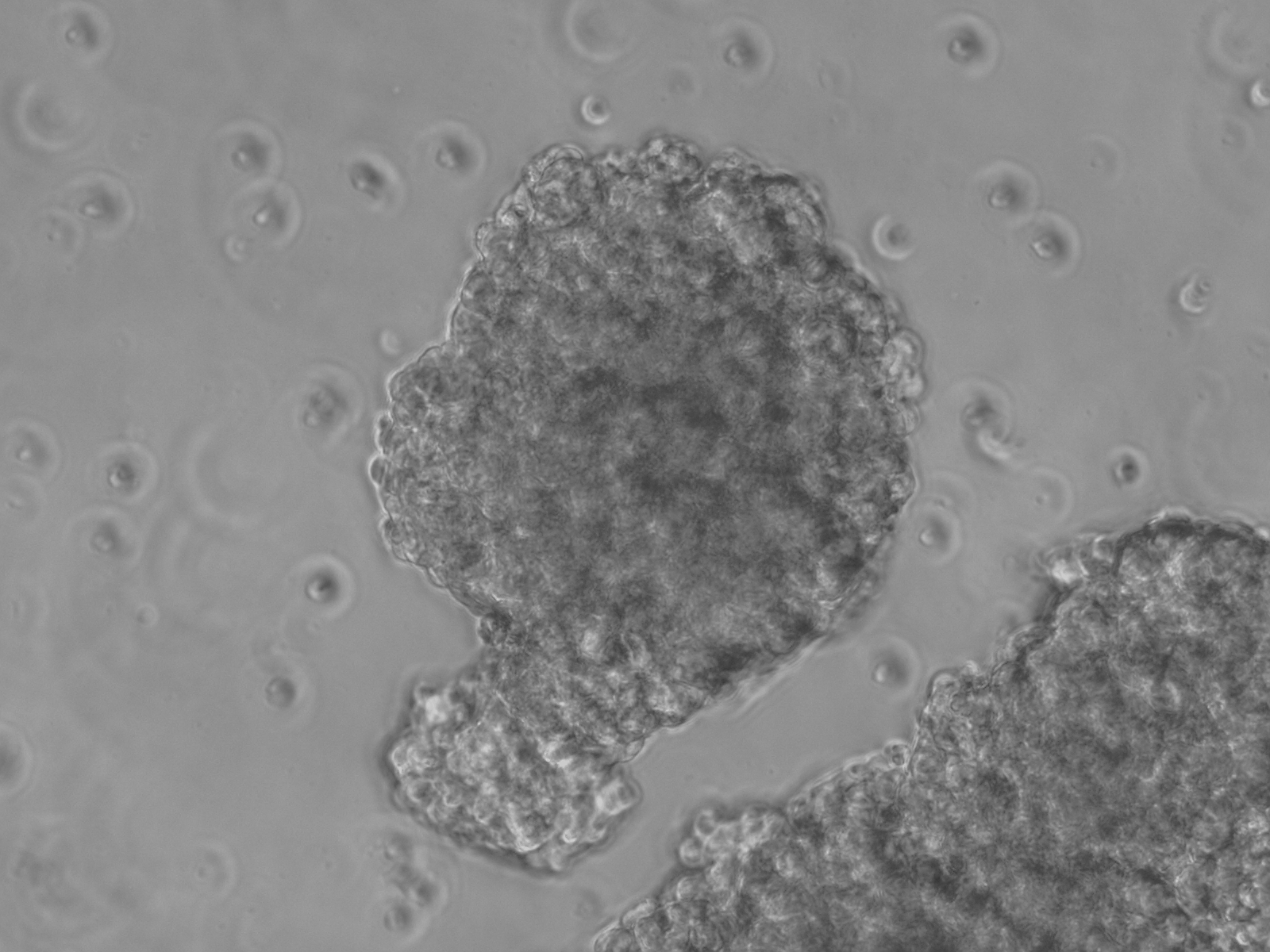

Supplement: Supplementary file 3 — Source data Fig. 2 [file 44321_2025_349_MOESM3_ESM.zip › Source data Figure 2/Fig2D/LUNB2/Control (DMSO)_Fig2D.tif]

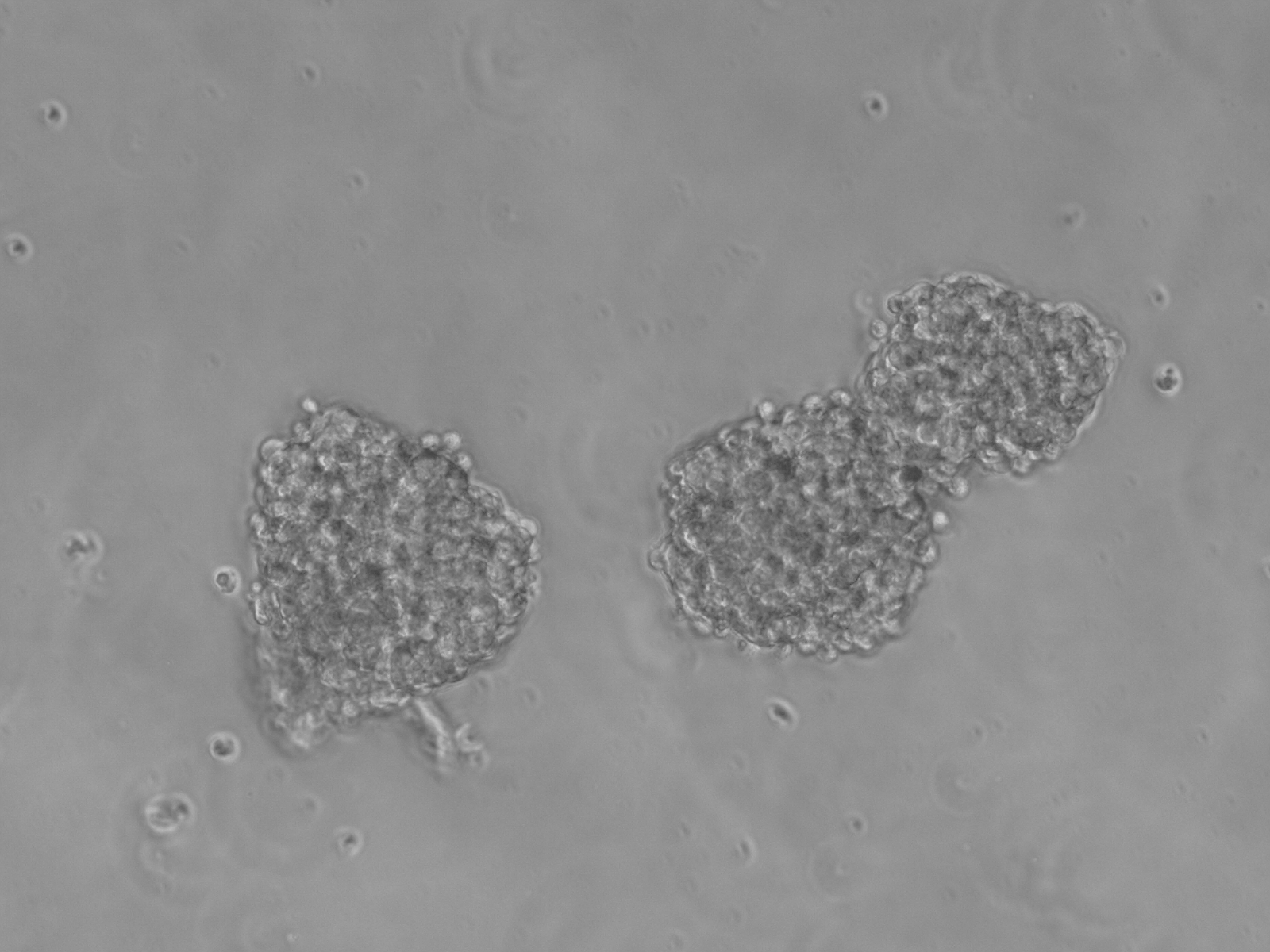

Supplement: Supplementary file 3 — Source data Fig. 2 [file 44321_2025_349_MOESM3_ESM.zip › Source data Figure 2/Fig2D/LUNB2/PCZ.tif]

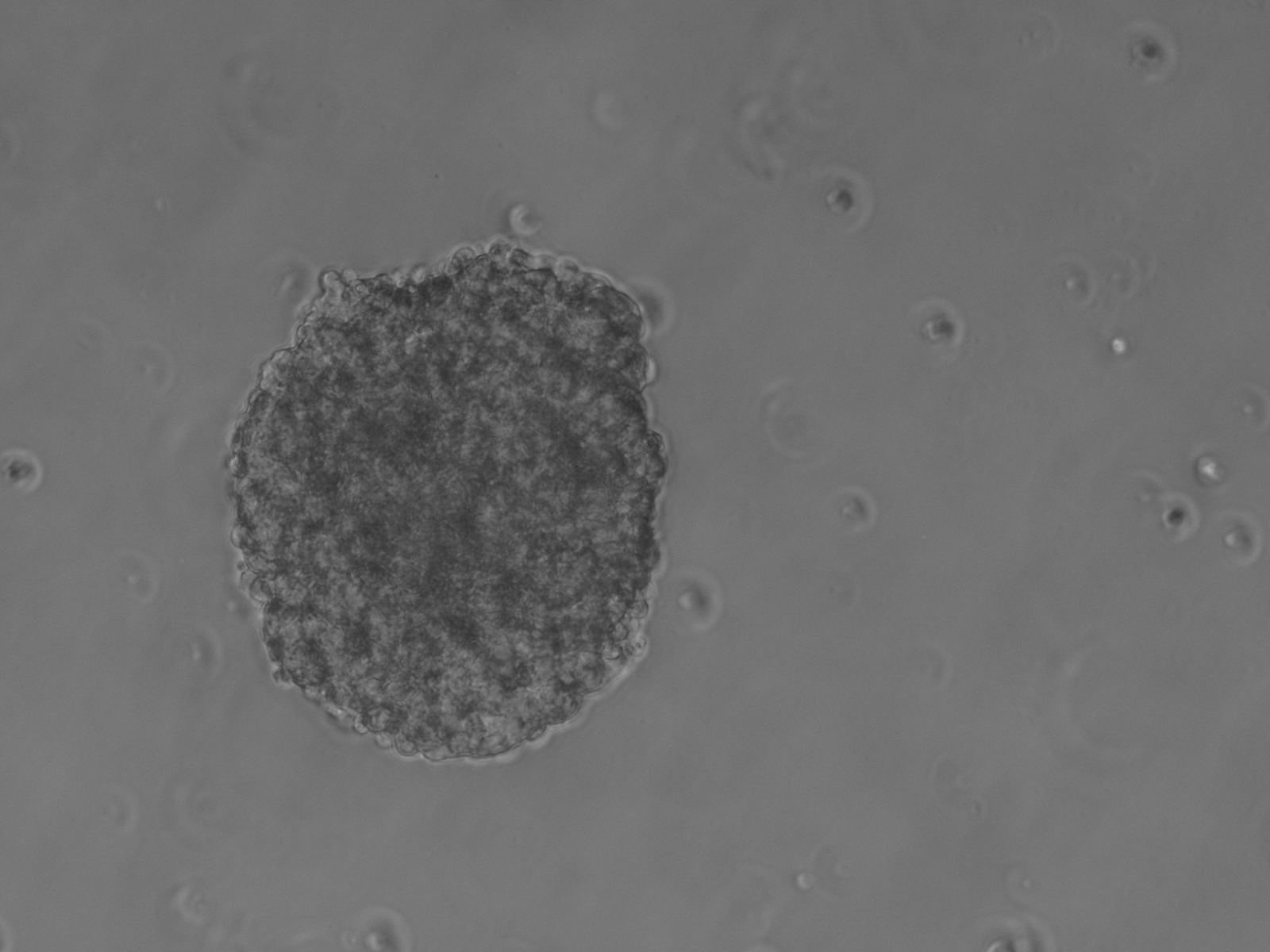

Supplement: Supplementary file 3 — Source data Fig. 2 [file 44321_2025_349_MOESM3_ESM.zip › Source data Figure 2/Fig2D/LUNB2/PCZ_Fig2D.tif]

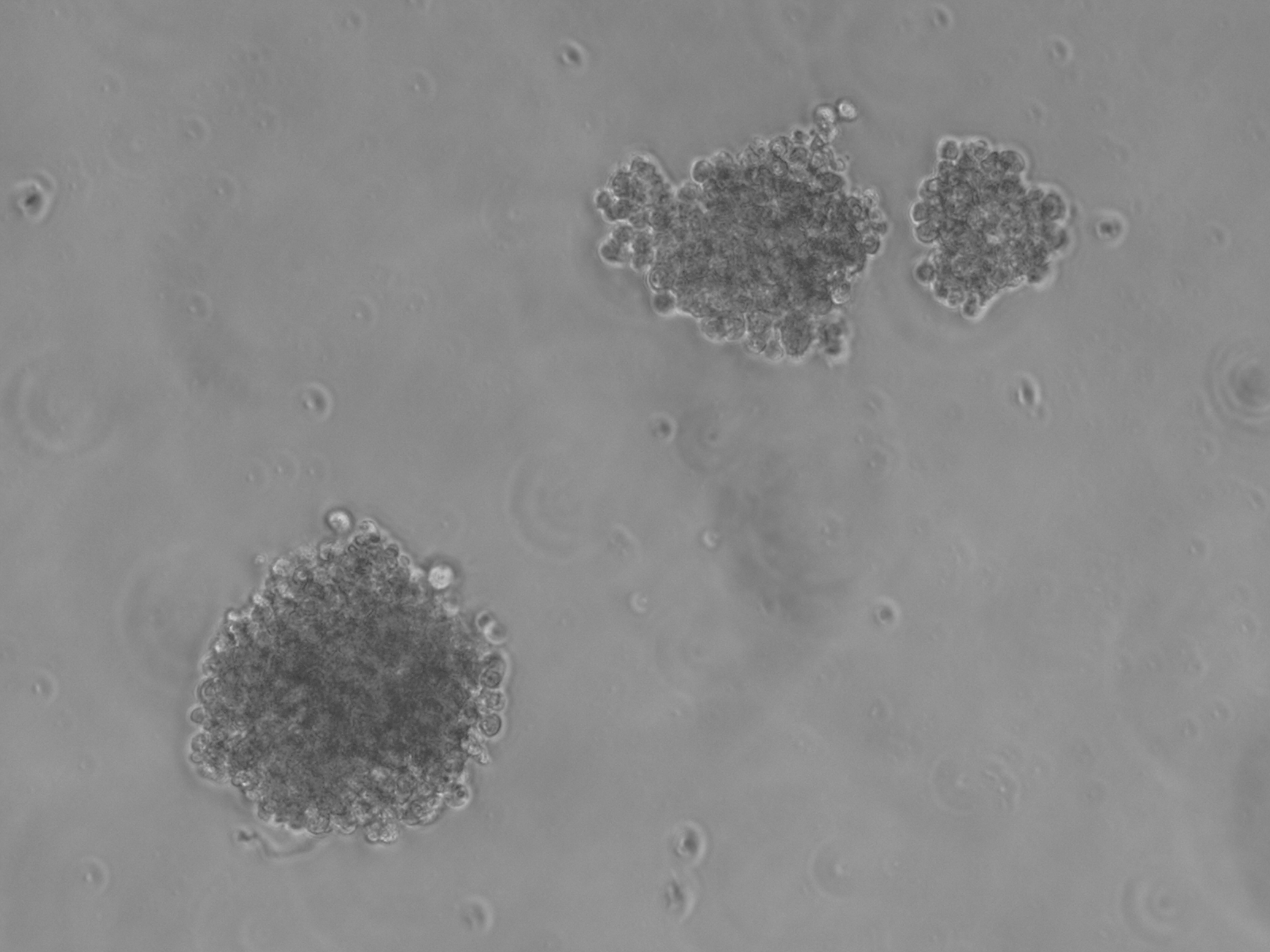

Supplement: Supplementary file 3 — Source data Fig. 2 [file 44321_2025_349_MOESM3_ESM.zip › Source data Figure 2/Fig2D/LUNB2/PIT.tif]

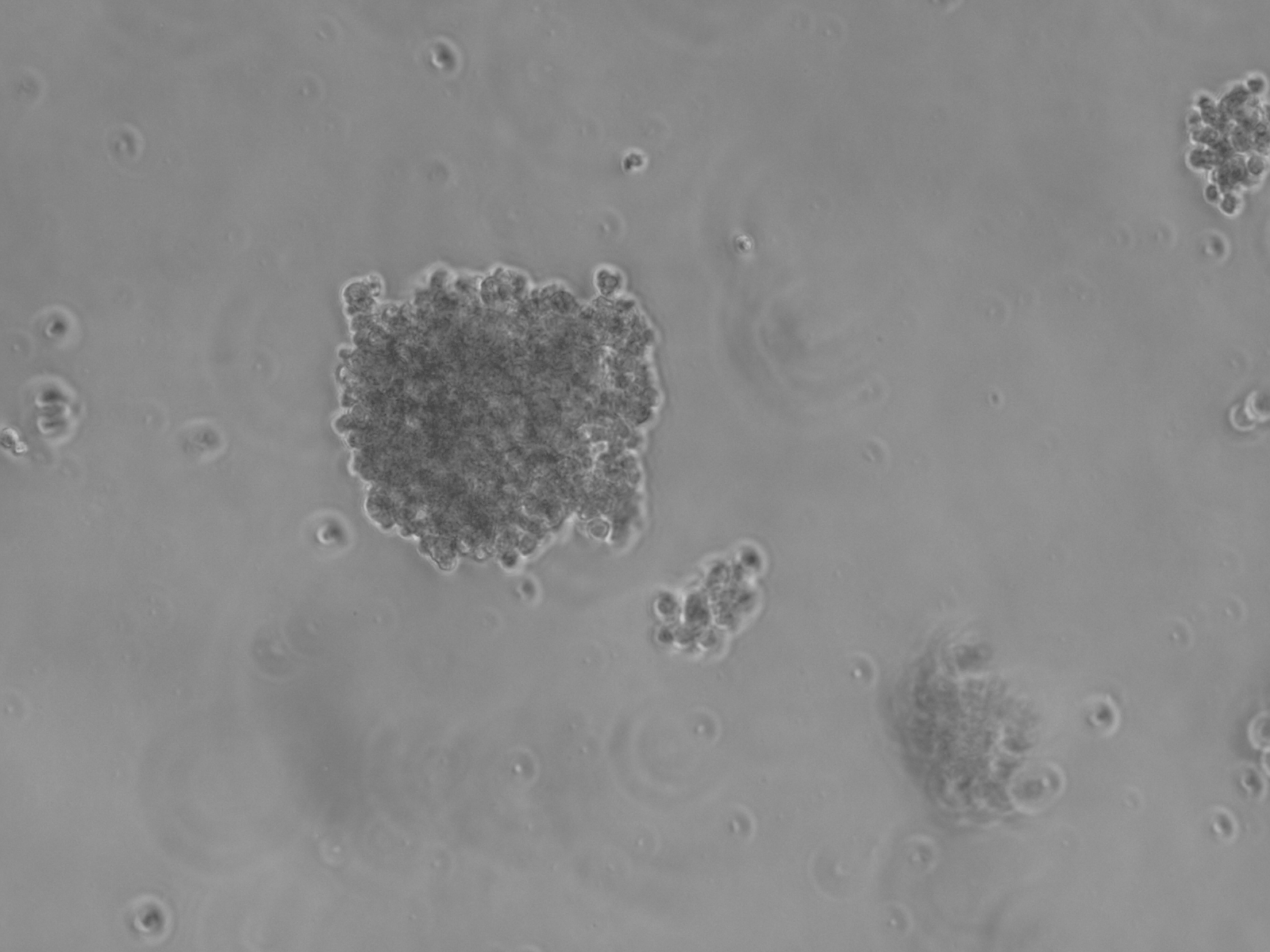

Supplement: Supplementary file 3 — Source data Fig. 2 [file 44321_2025_349_MOESM3_ESM.zip › Source data Figure 2/Fig2D/LUNB2/PIT_Fig2D.tif]

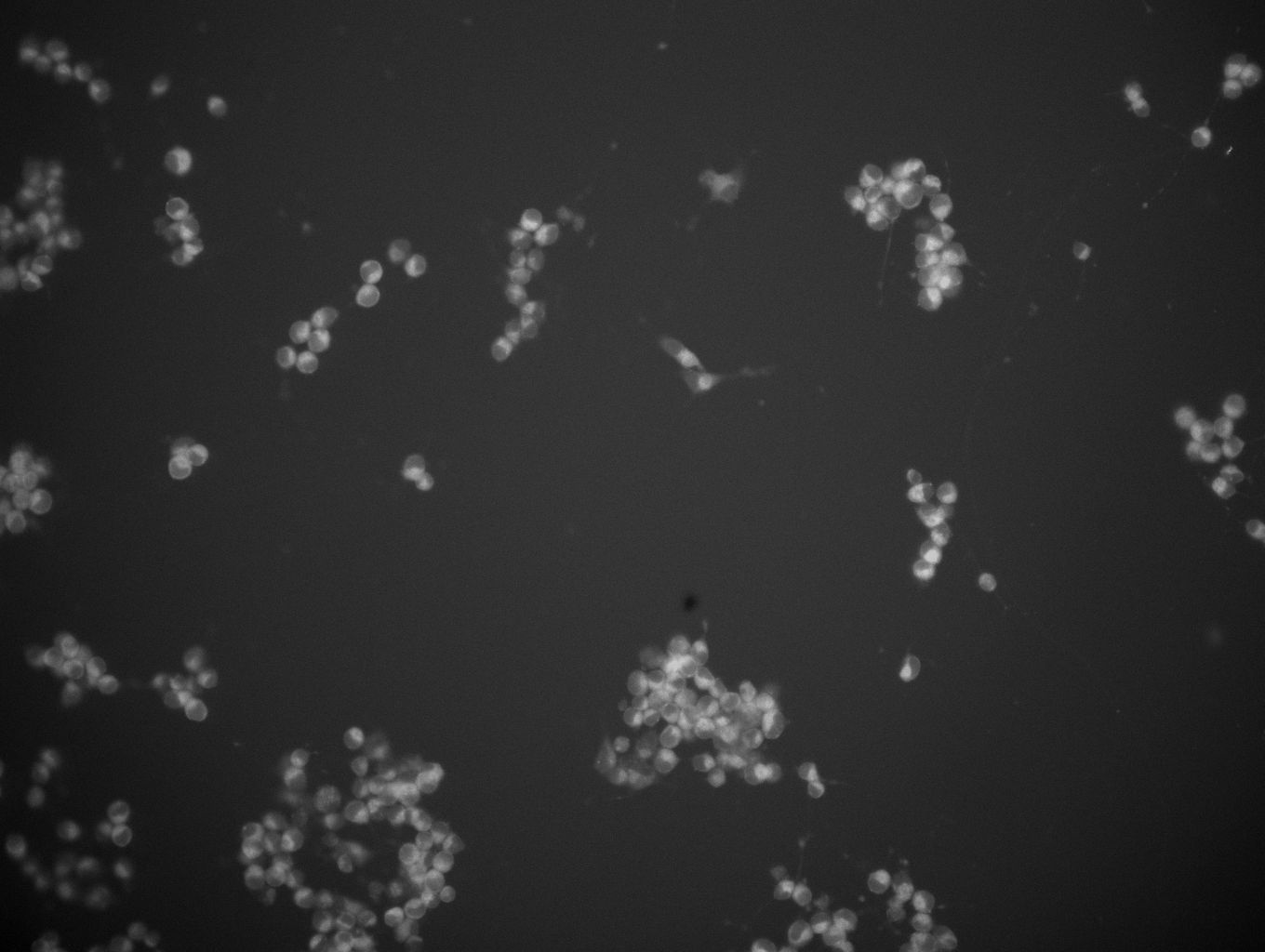

Supplement: Supplementary file 5 — Source data Fig. 4 [file 44321_2025_349_MOESM5_ESM.zip › Source data Figure 4/Fig4E/R1/Combination_PIT_PCZ_Representative photos/10x/PCPPIT_FL20240112104.tif]

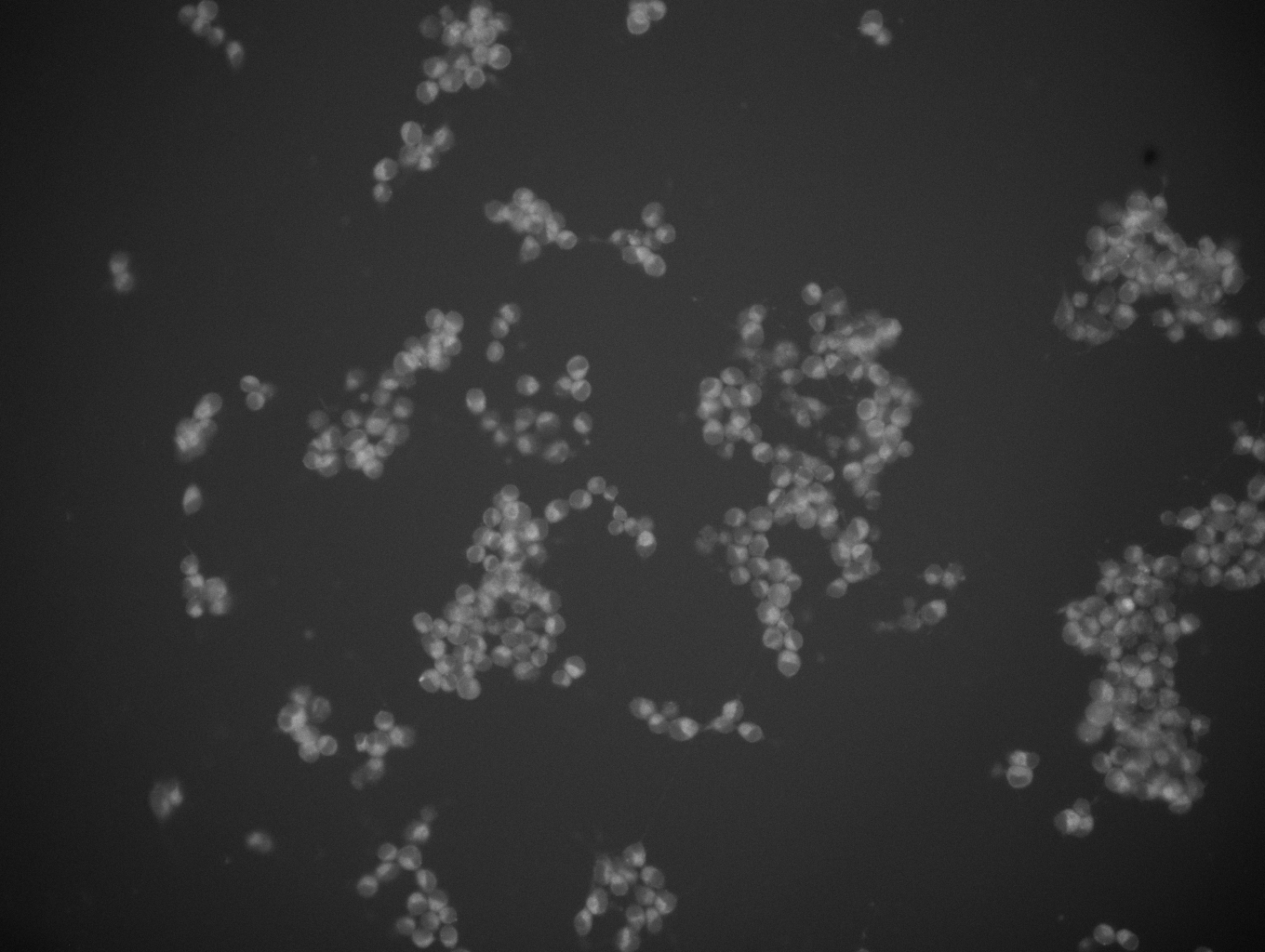

Supplement: Supplementary file 5 — Source data Fig. 4 [file 44321_2025_349_MOESM5_ESM.zip › Source data Figure 4/Fig4E/R1/Combination_PIT_PCZ_Representative photos/10x/PCPPIT_FL20240112105.tif]

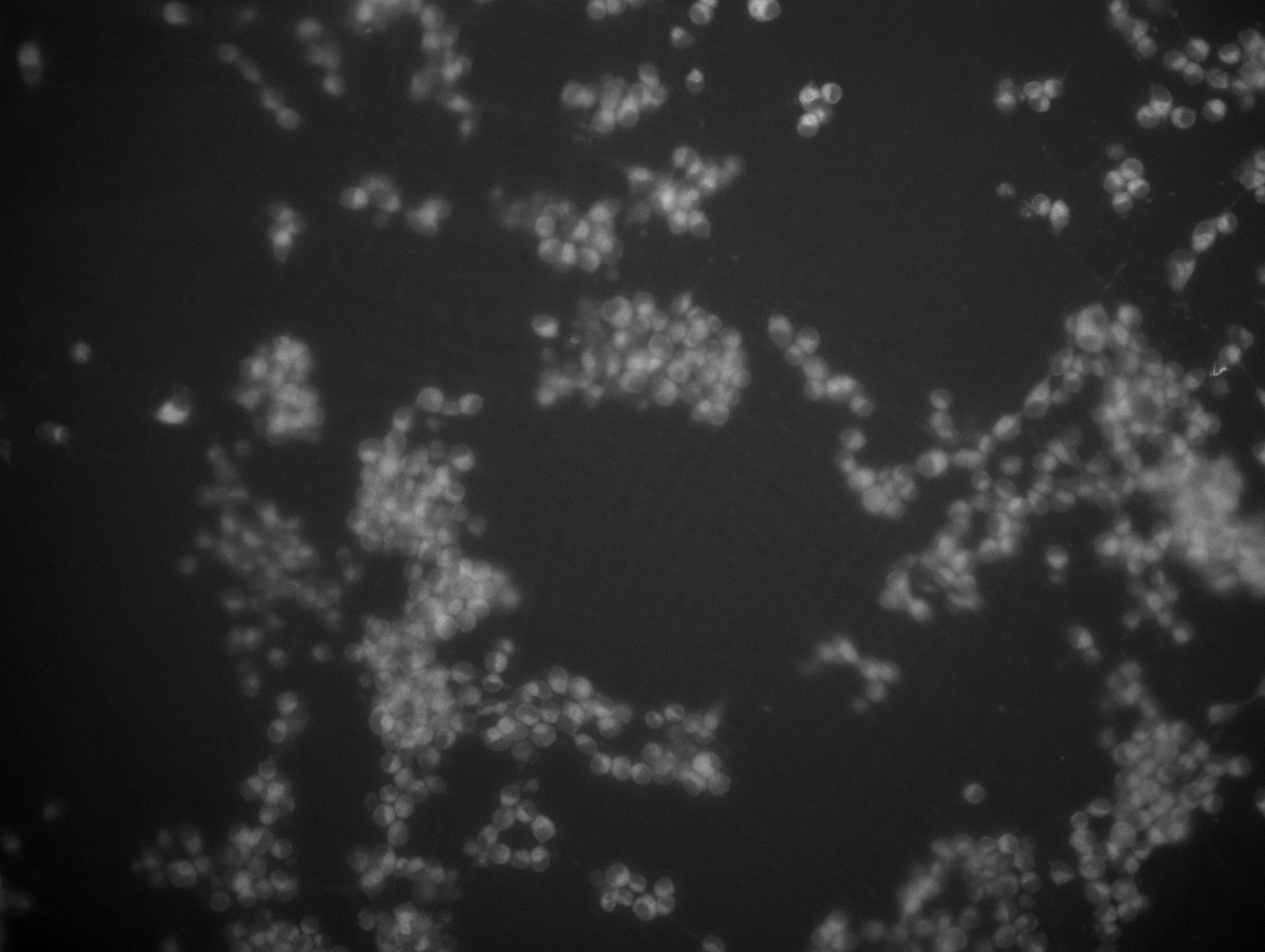

Supplement: Supplementary file 5 — Source data Fig. 4 [file 44321_2025_349_MOESM5_ESM.zip › Source data Figure 4/Fig4E/R1/Combination_PIT_PCZ_Representative photos/10x/PCPPIT_FL20240112113.tif]

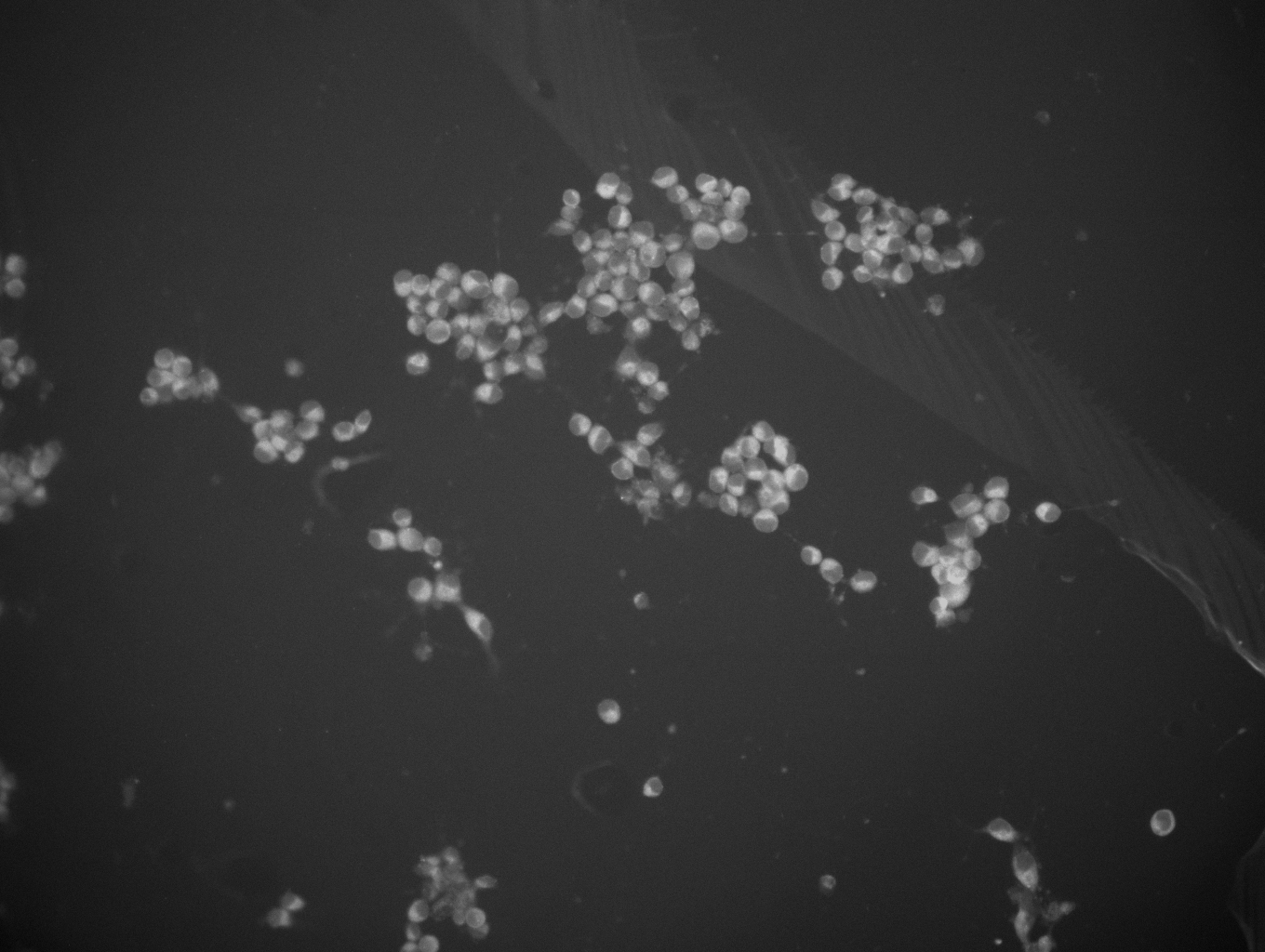

Supplement: Supplementary file 5 — Source data Fig. 4 [file 44321_2025_349_MOESM5_ESM.zip › Source data Figure 4/Fig4E/R1/Combination_PIT_PCZ_Representative photos/10x/PCPPIT_FL20240112130.tif]

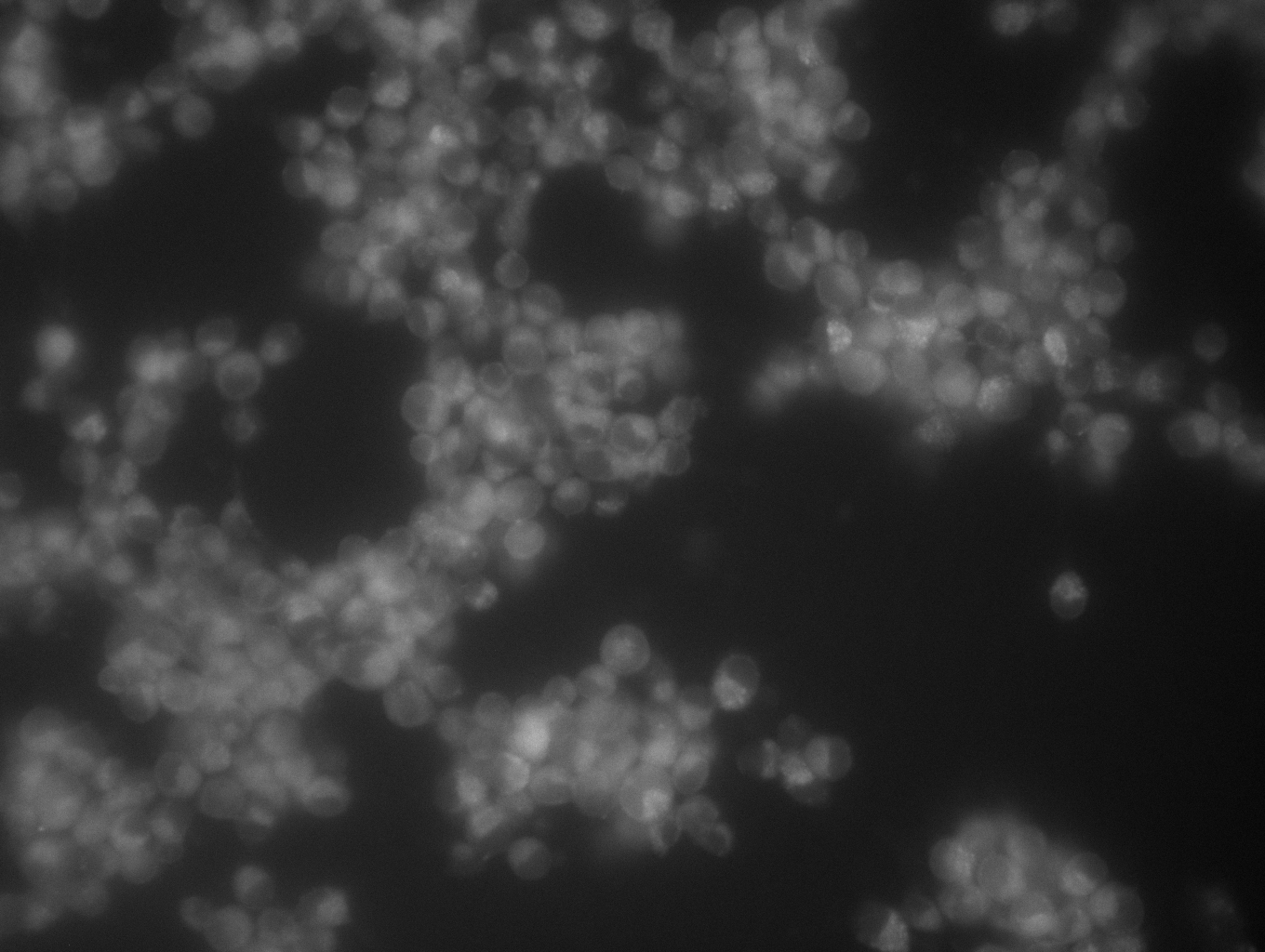

Supplement: Supplementary file 5 — Source data Fig. 4 [file 44321_2025_349_MOESM5_ESM.zip › Source data Figure 4/Fig4E/R1/Combination_PIT_PCZ_Representative photos/20x/PCPPIT_FL20240112117.tif]

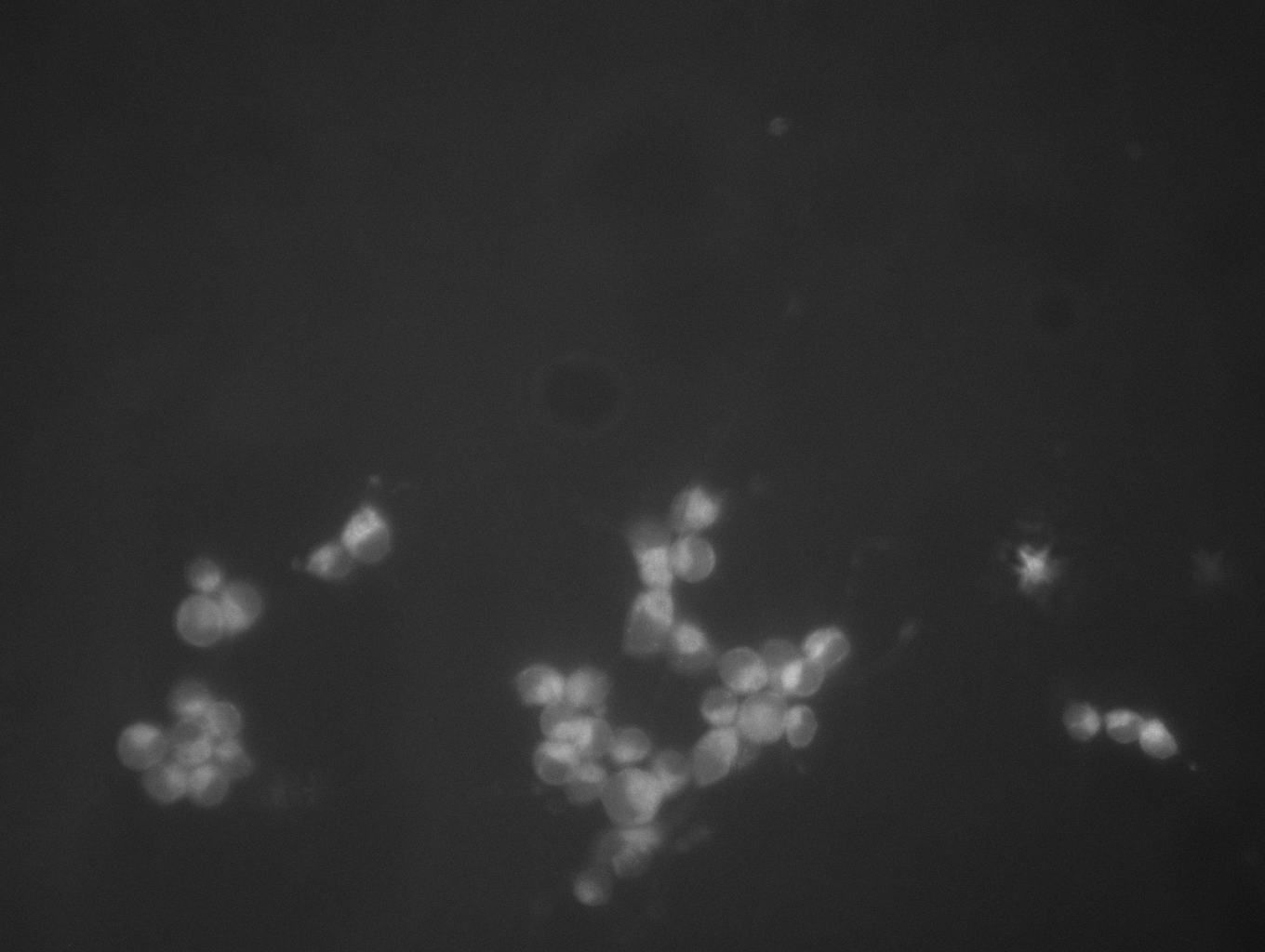

Supplement: Supplementary file 5 — Source data Fig. 4 [file 44321_2025_349_MOESM5_ESM.zip › Source data Figure 4/Fig4E/R1/Combination_PIT_PCZ_Representative photos/20x/PCPPIT_FL20240112119.tif]

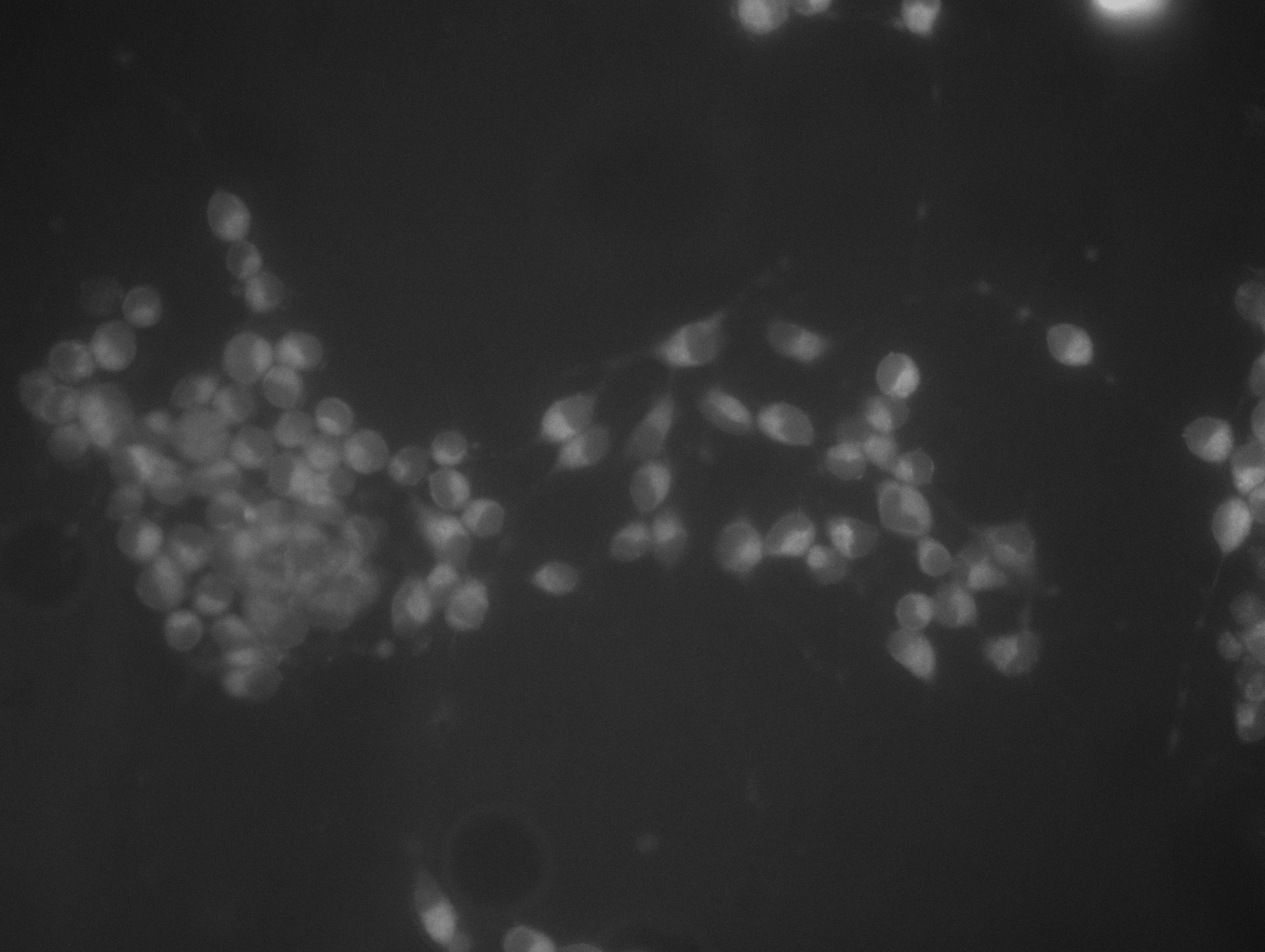

Supplement: Supplementary file 5 — Source data Fig. 4 [file 44321_2025_349_MOESM5_ESM.zip › Source data Figure 4/Fig4E/R1/Combination_PIT_PCZ_Representative photos/20x/PCPPIT_FL20240112120.tif]

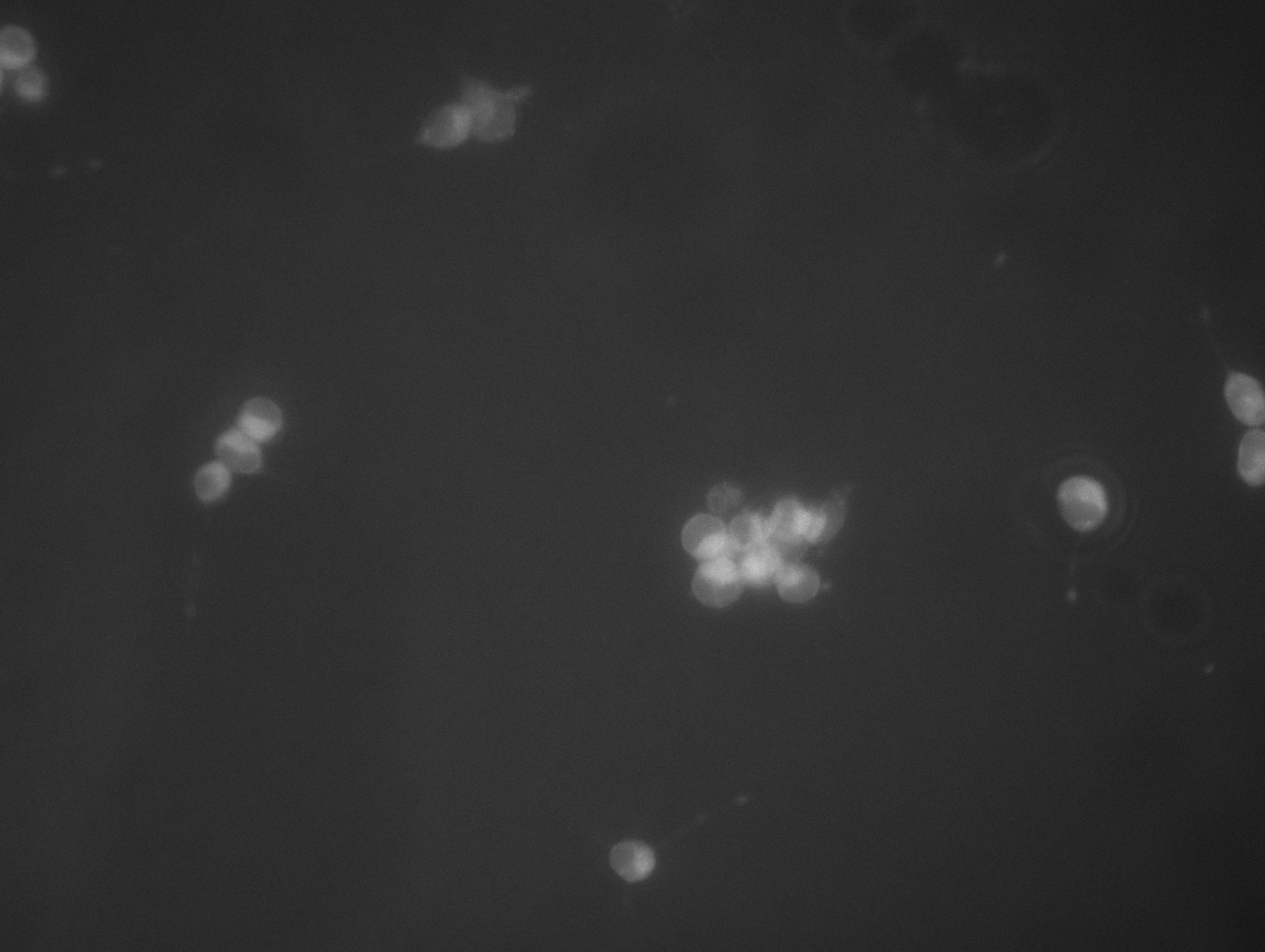

Supplement: Supplementary file 5 — Source data Fig. 4 [file 44321_2025_349_MOESM5_ESM.zip › Source data Figure 4/Fig4E/R1/Combination_PIT_PCZ_Representative photos/20x/PCPPIT_FL20240112121.tif]

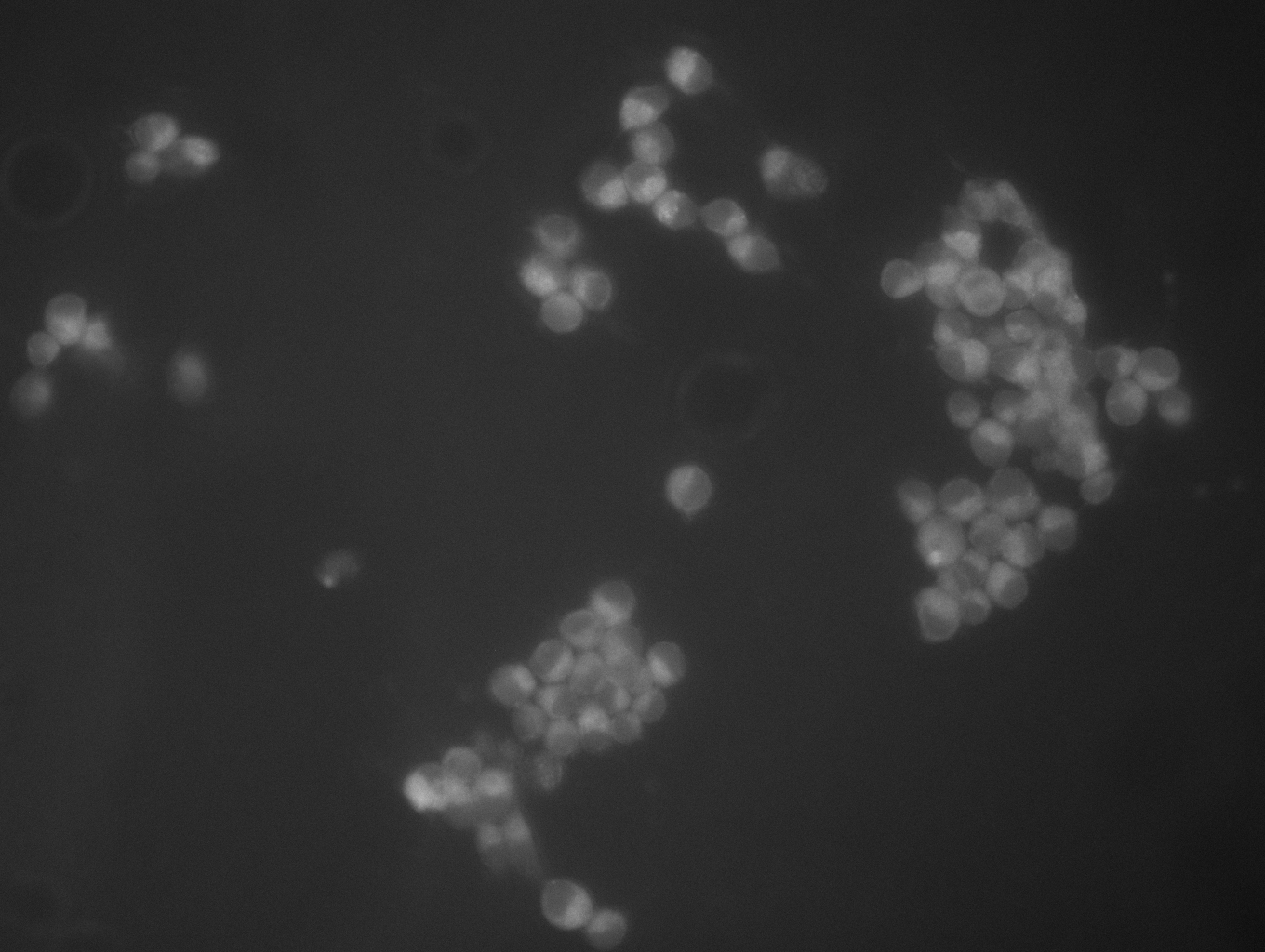

Supplement: Supplementary file 5 — Source data Fig. 4 [file 44321_2025_349_MOESM5_ESM.zip › Source data Figure 4/Fig4E/R1/Combination_PIT_PCZ_Representative photos/20x/PCPPIT_FL20240112122.tif]

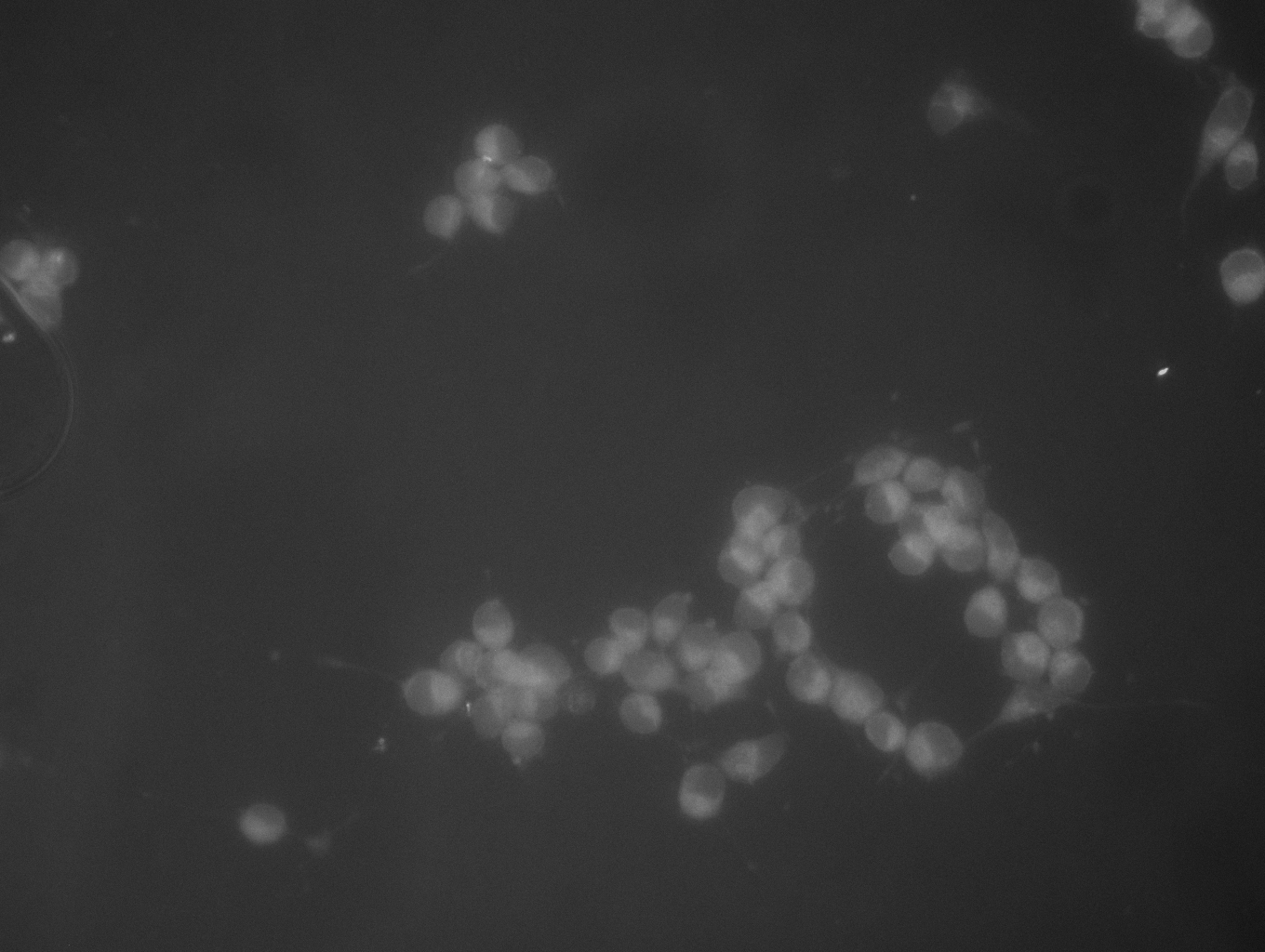

Supplement: Supplementary file 5 — Source data Fig. 4 [file 44321_2025_349_MOESM5_ESM.zip › Source data Figure 4/Fig4E/R1/Combination_PIT_PCZ_Representative photos/20x/PCPPIT_FL20240112133.tif]

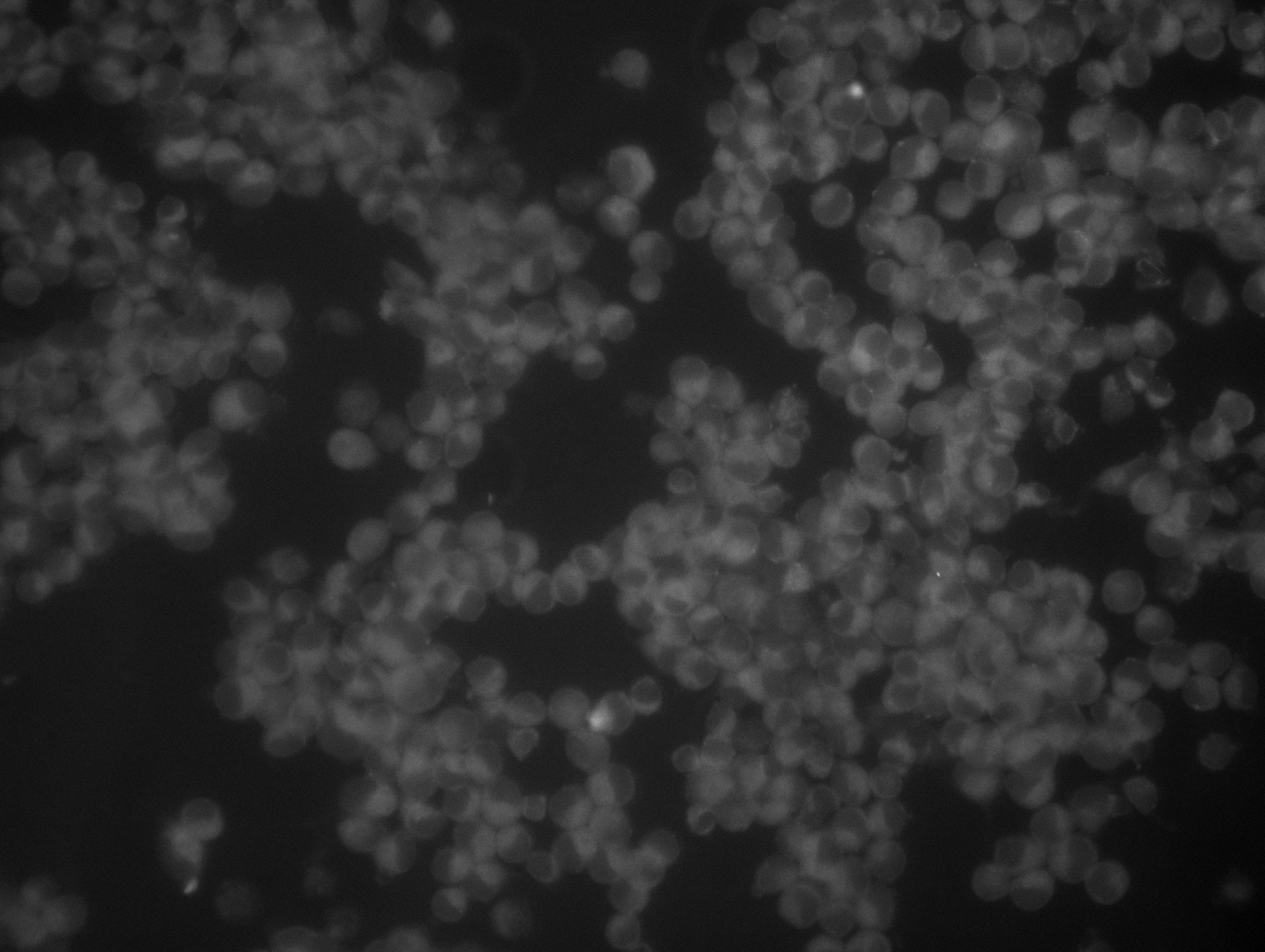

Supplement: Supplementary file 5 — Source data Fig. 4 [file 44321_2025_349_MOESM5_ESM.zip › Source data Figure 4/Fig4E/R1/Combination_PIT_PCZ_Representative photos/20x/PCPPIT_FL20240112134.tif]

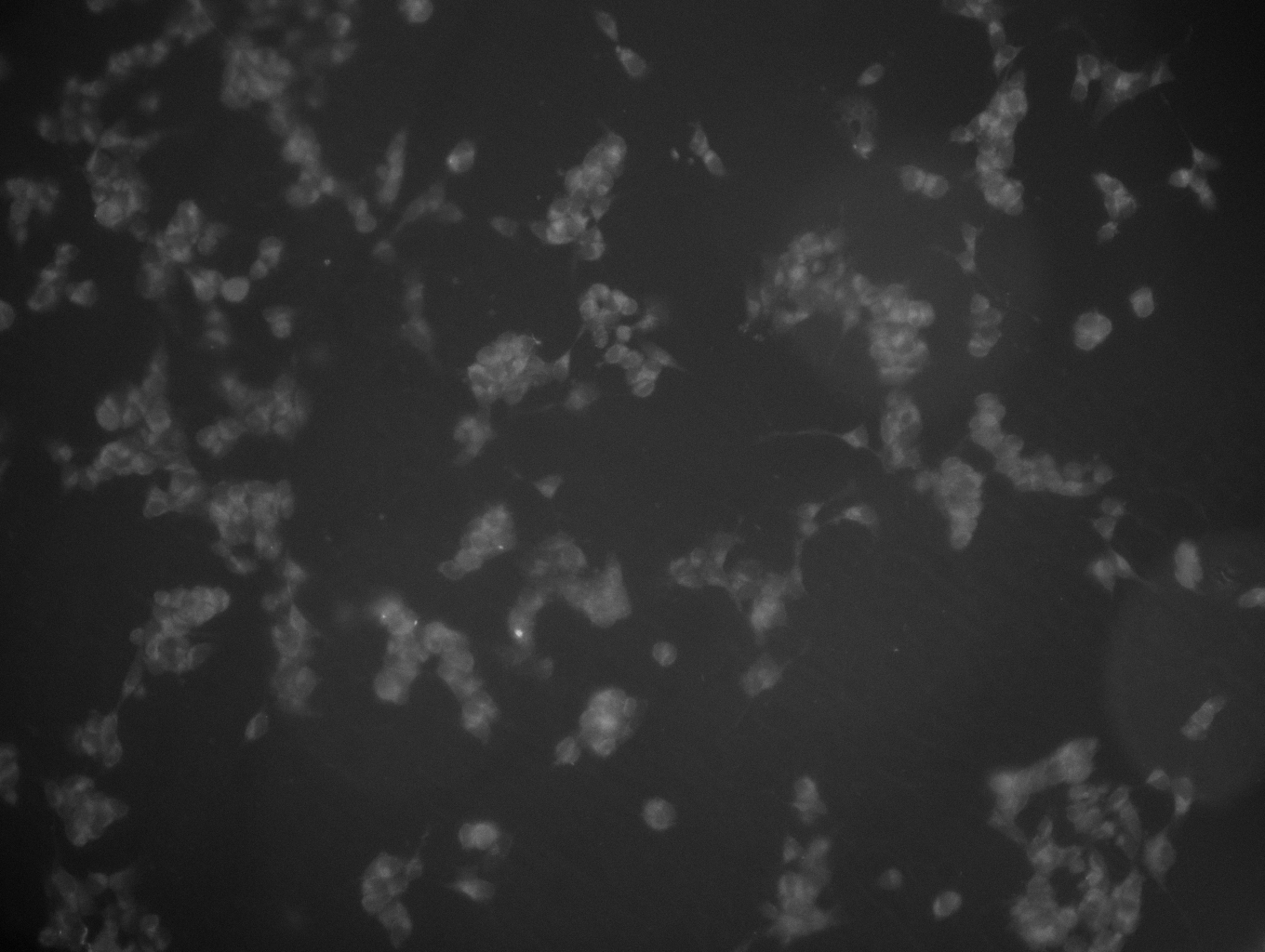

Supplement: Supplementary file 5 — Source data Fig. 4 [file 44321_2025_349_MOESM5_ESM.zip › Source data Figure 4/Fig4E/R1/DMSO Representative photos/10x/_FL20240112142DMSO.tif]

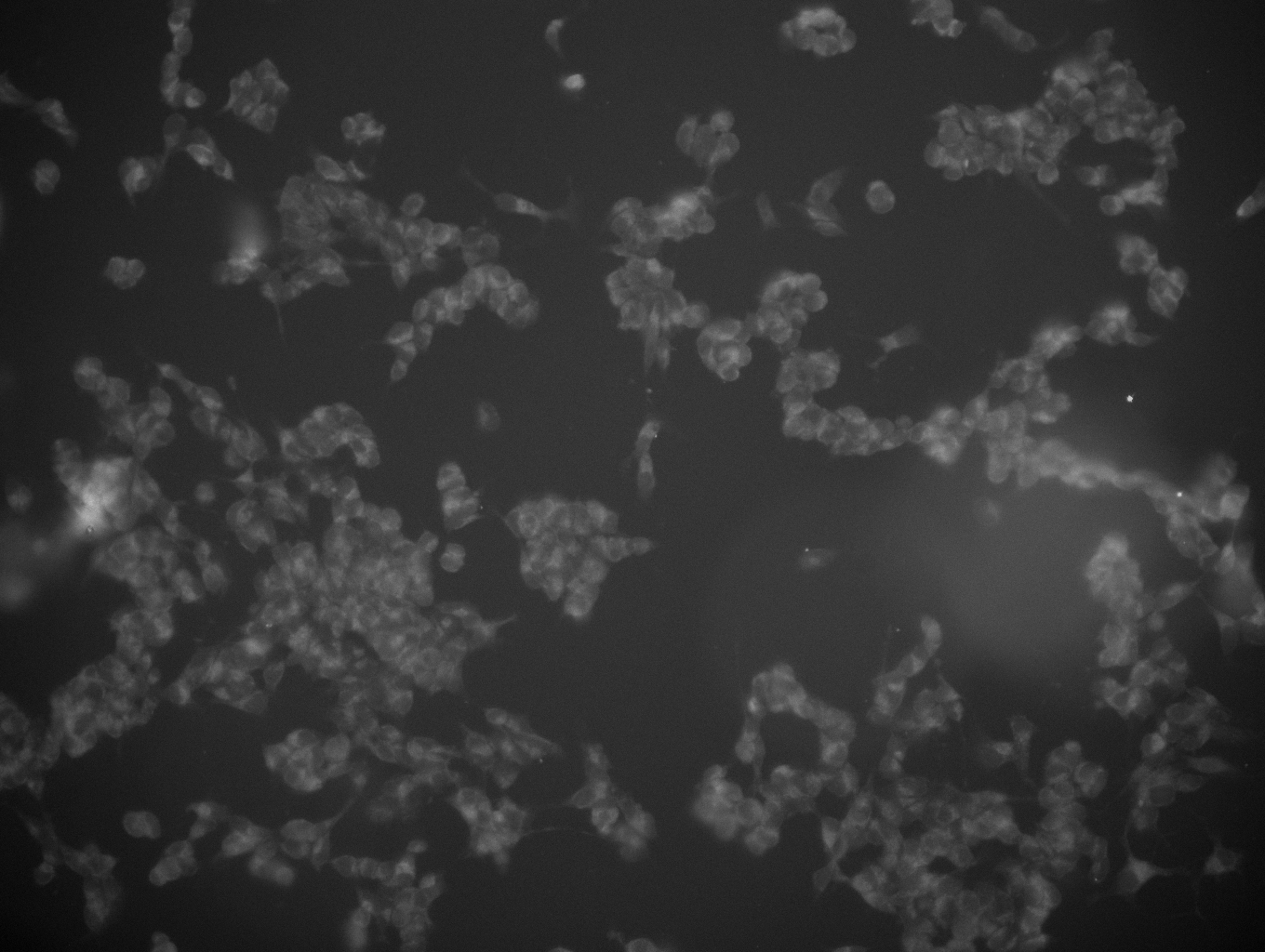

Supplement: Supplementary file 5 — Source data Fig. 4 [file 44321_2025_349_MOESM5_ESM.zip › Source data Figure 4/Fig4E/R1/DMSO Representative photos/10x/_FL20240112143DMSO.tif]

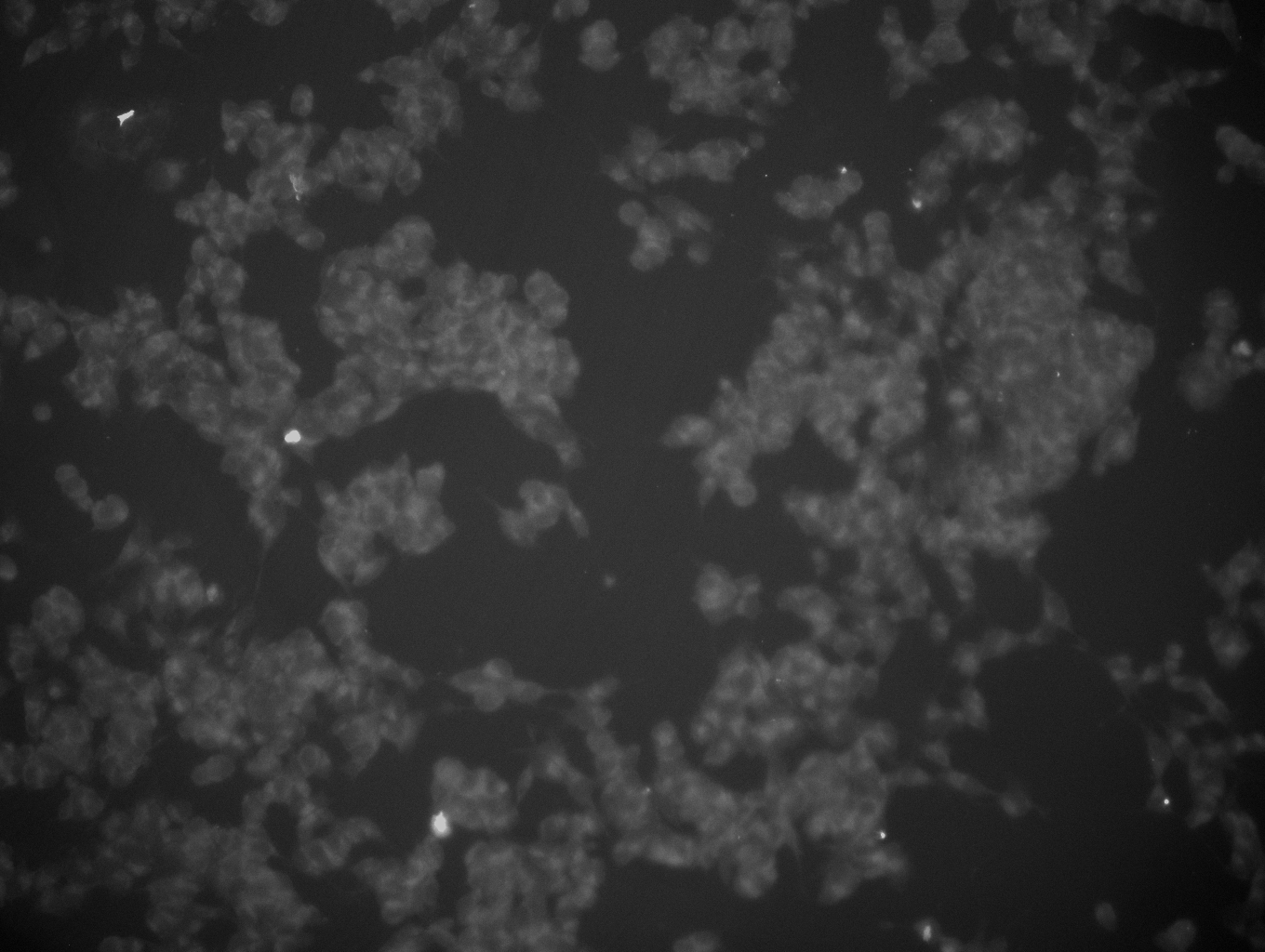

Supplement: Supplementary file 5 — Source data Fig. 4 [file 44321_2025_349_MOESM5_ESM.zip › Source data Figure 4/Fig4E/R1/DMSO Representative photos/10x/_FL20240112150DMSO.tif]

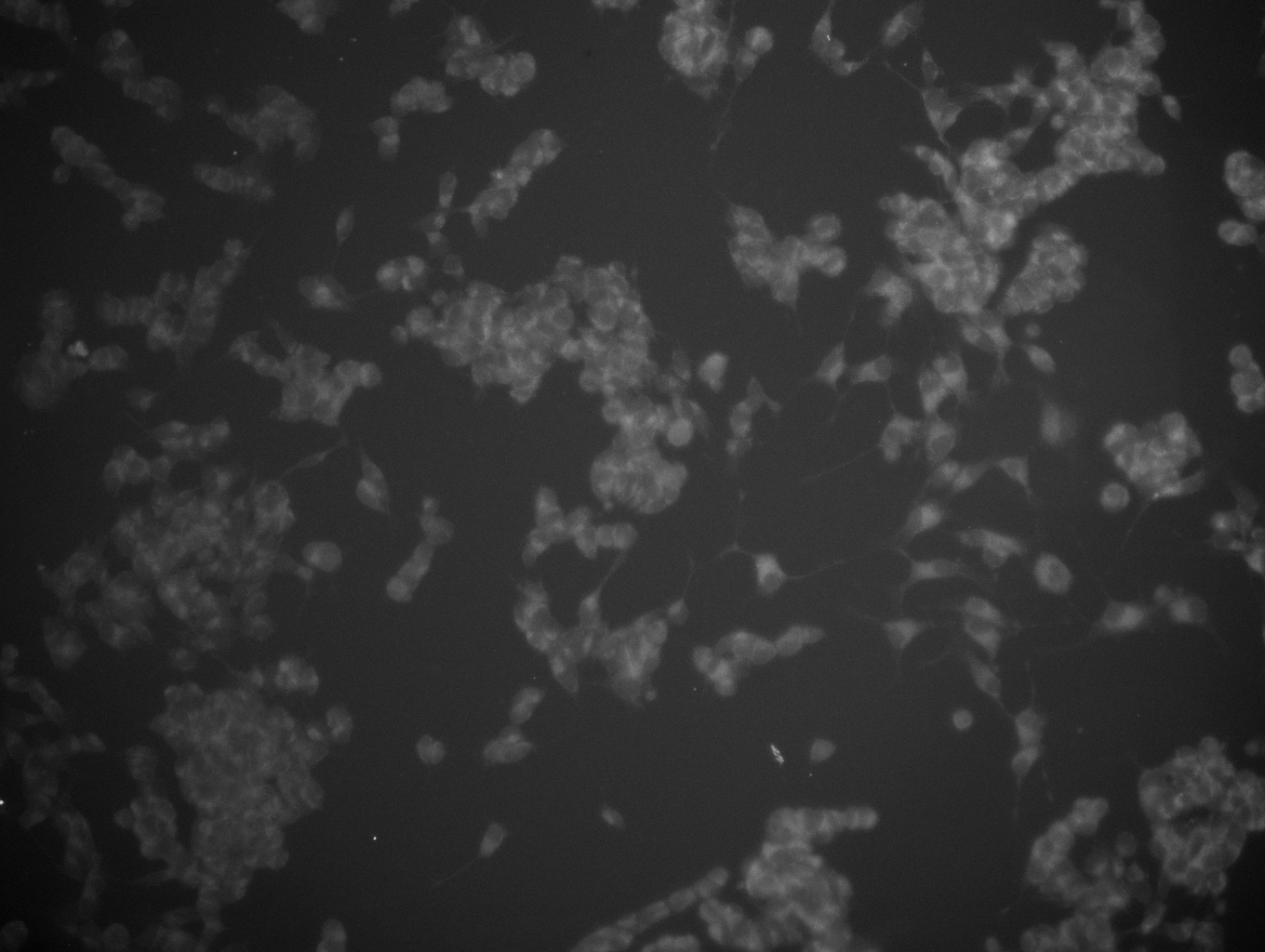

Supplement: Supplementary file 5 — Source data Fig. 4 [file 44321_2025_349_MOESM5_ESM.zip › Source data Figure 4/Fig4E/R1/DMSO Representative photos/10x/_FL20240112151DMSO.tif]

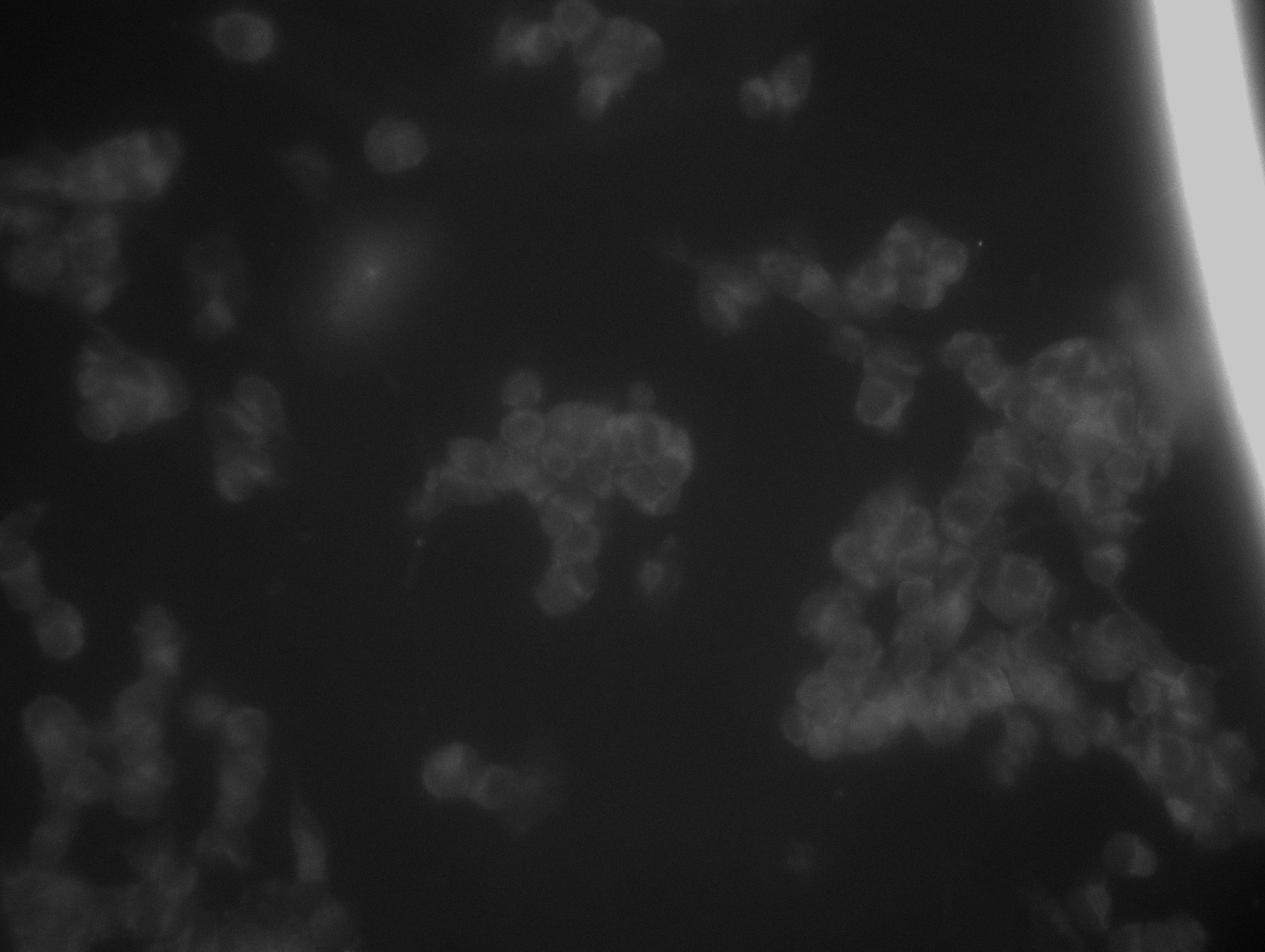

Supplement: Supplementary file 5 — Source data Fig. 4 [file 44321_2025_349_MOESM5_ESM.zip › Source data Figure 4/Fig4E/R1/DMSO Representative photos/20x/_FL20240112146DMSO.tif]

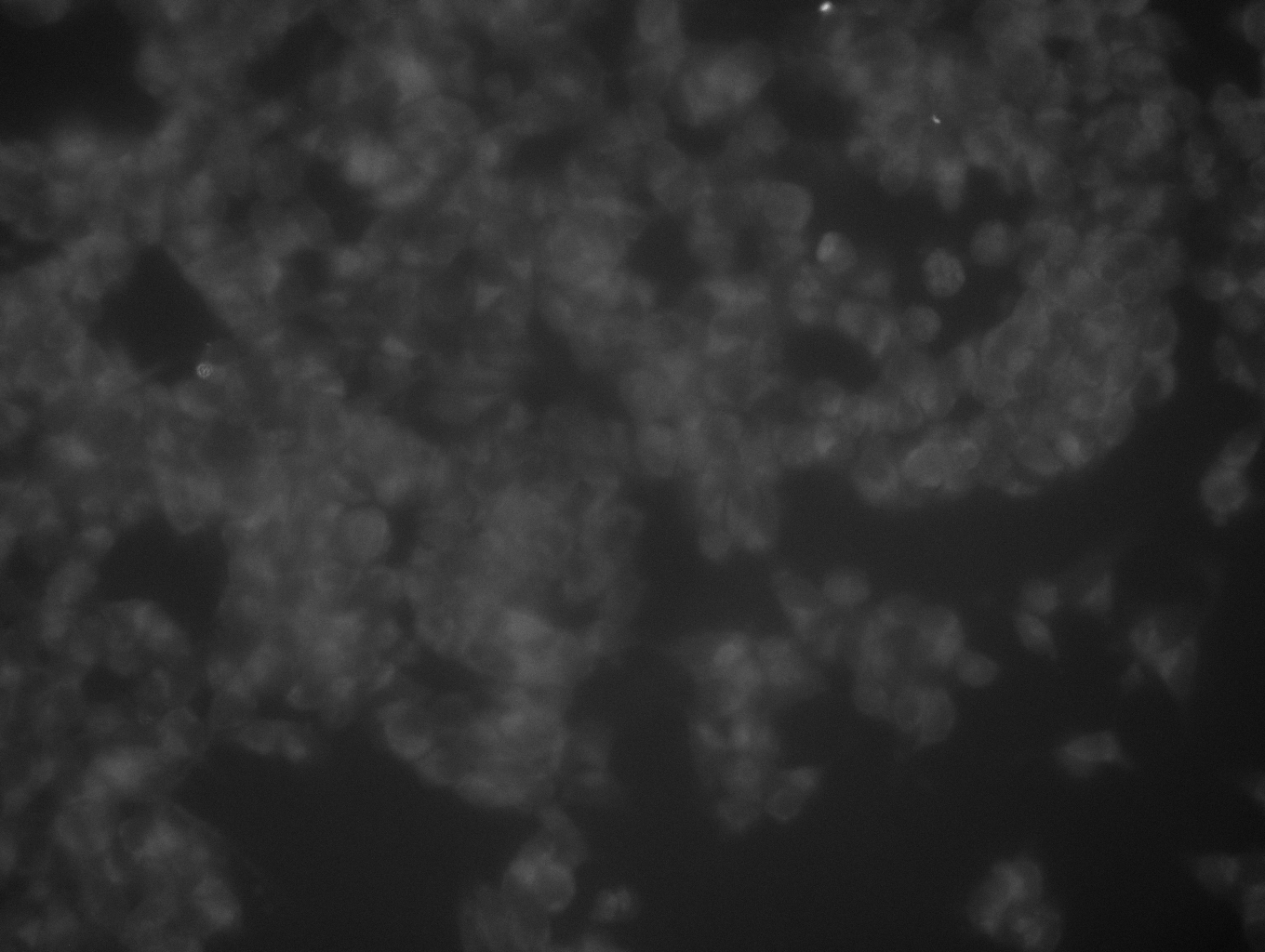

Supplement: Supplementary file 5 — Source data Fig. 4 [file 44321_2025_349_MOESM5_ESM.zip › Source data Figure 4/Fig4E/R1/DMSO Representative photos/20x/_FL20240112147DMSO.tif]

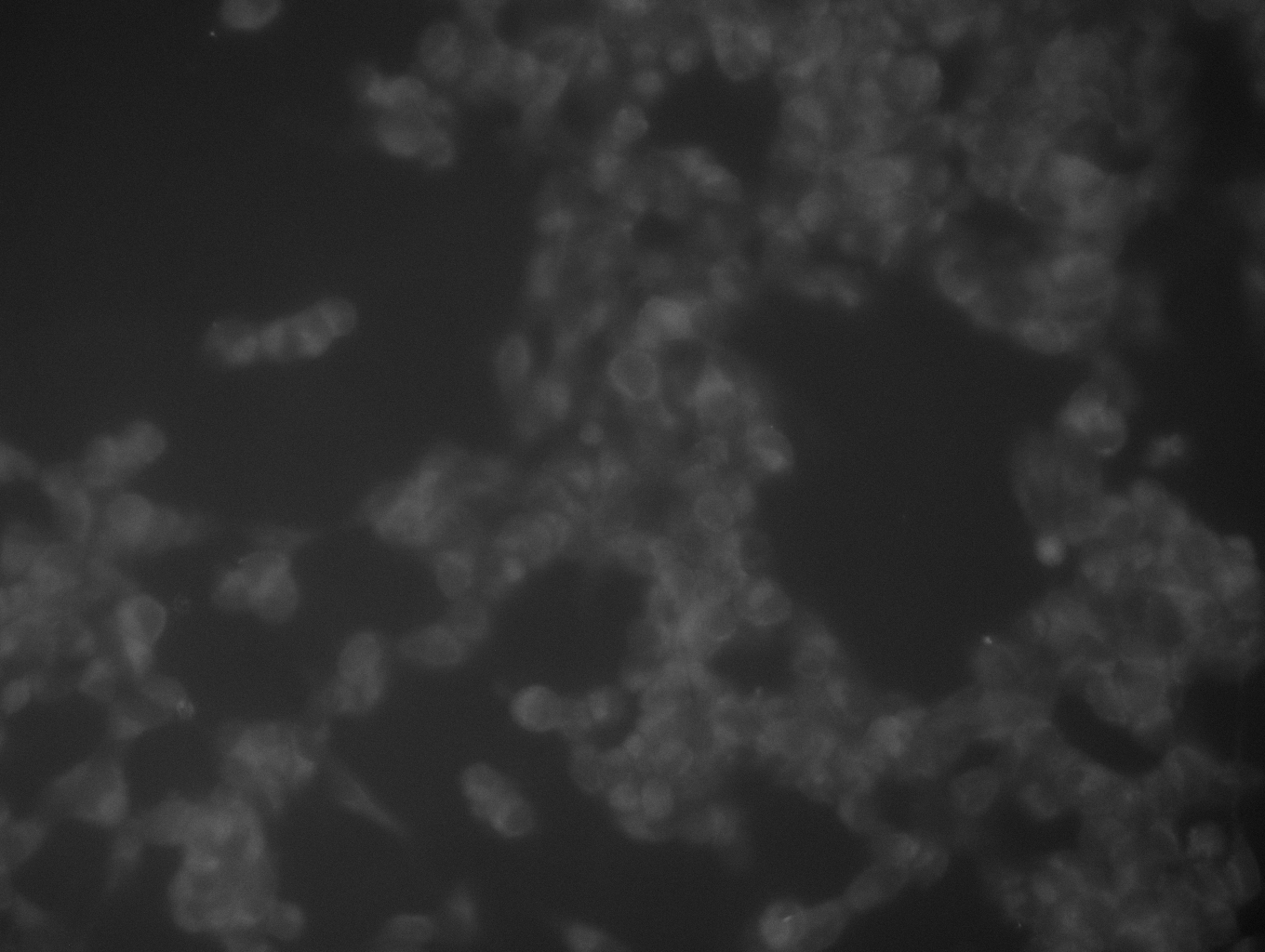

Supplement: Supplementary file 5 — Source data Fig. 4 [file 44321_2025_349_MOESM5_ESM.zip › Source data Figure 4/Fig4E/R1/DMSO Representative photos/20x/_FL20240112148DMSO.tif]

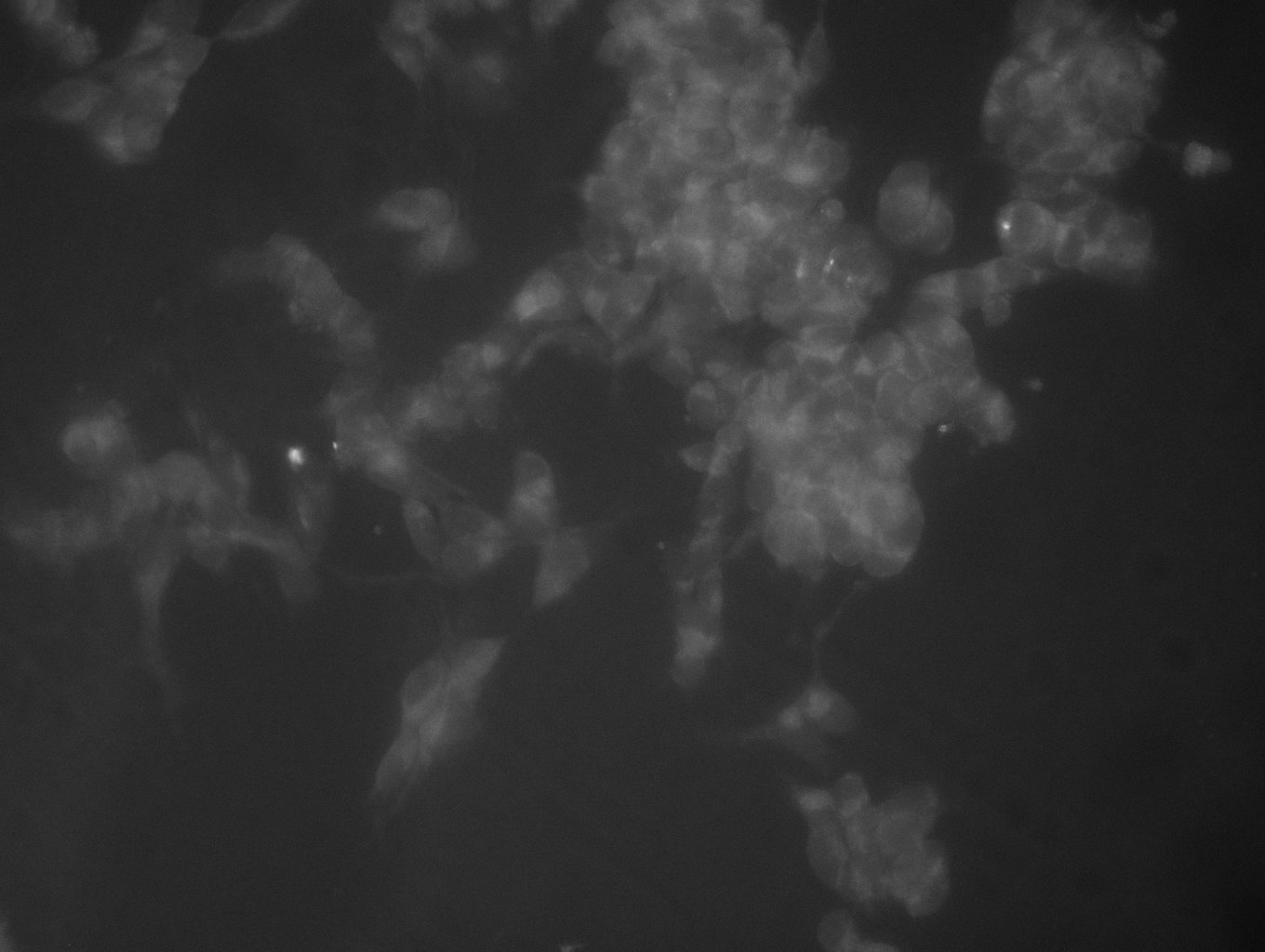

Supplement: Supplementary file 5 — Source data Fig. 4 [file 44321_2025_349_MOESM5_ESM.zip › Source data Figure 4/Fig4E/R1/DMSO Representative photos/20x/_FL20240112152DMSO.tif]

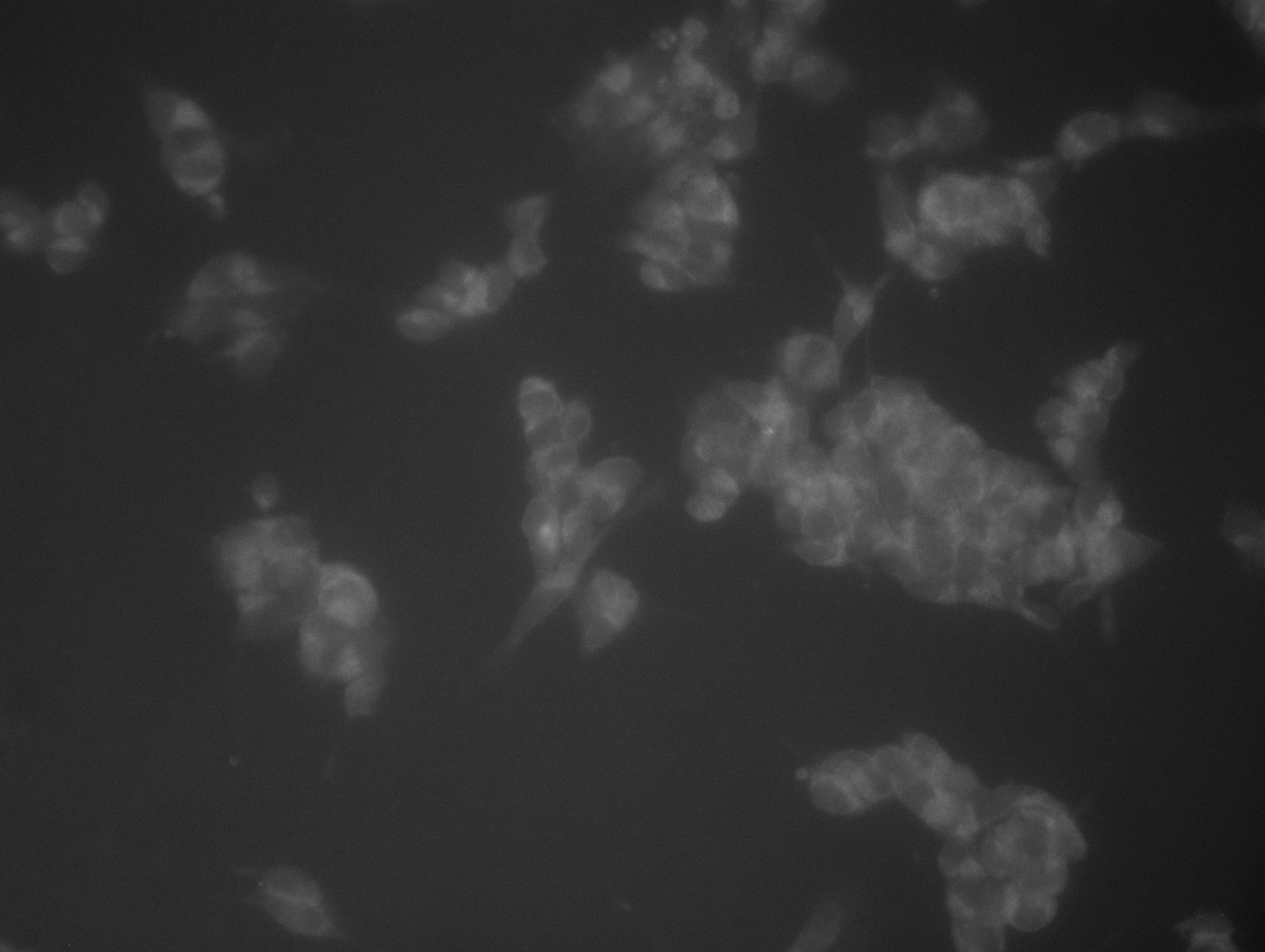

Supplement: Supplementary file 5 — Source data Fig. 4 [file 44321_2025_349_MOESM5_ESM.zip › Source data Figure 4/Fig4E/R1/DMSO Representative photos/20x/_FL20240112157DMSO.tif]

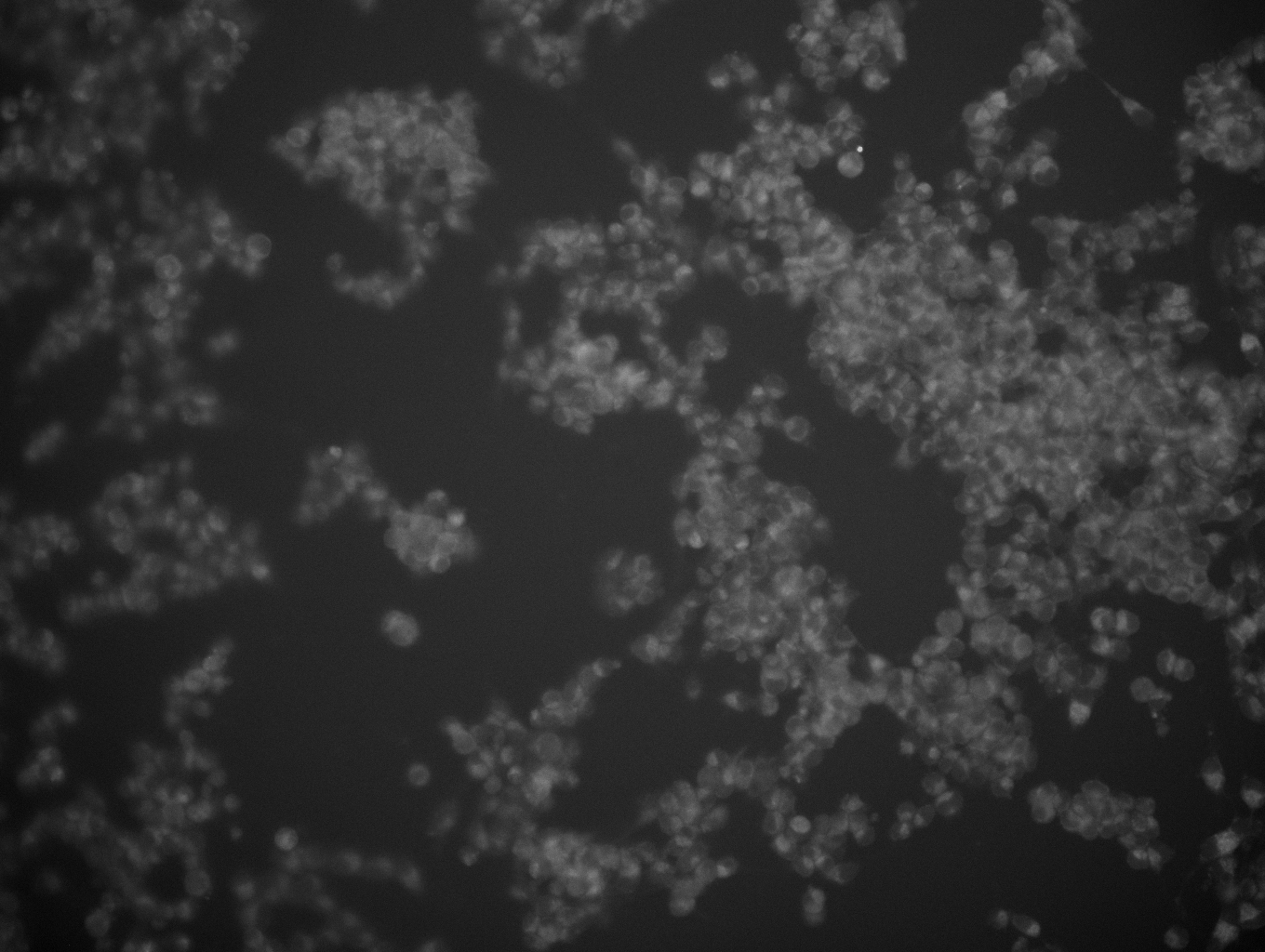

Supplement: Supplementary file 5 — Source data Fig. 4 [file 44321_2025_349_MOESM5_ESM.zip › Source data Figure 4/Fig4E/R1/PCZ Representative photos/10x/PCP_FL20240112059.tif]

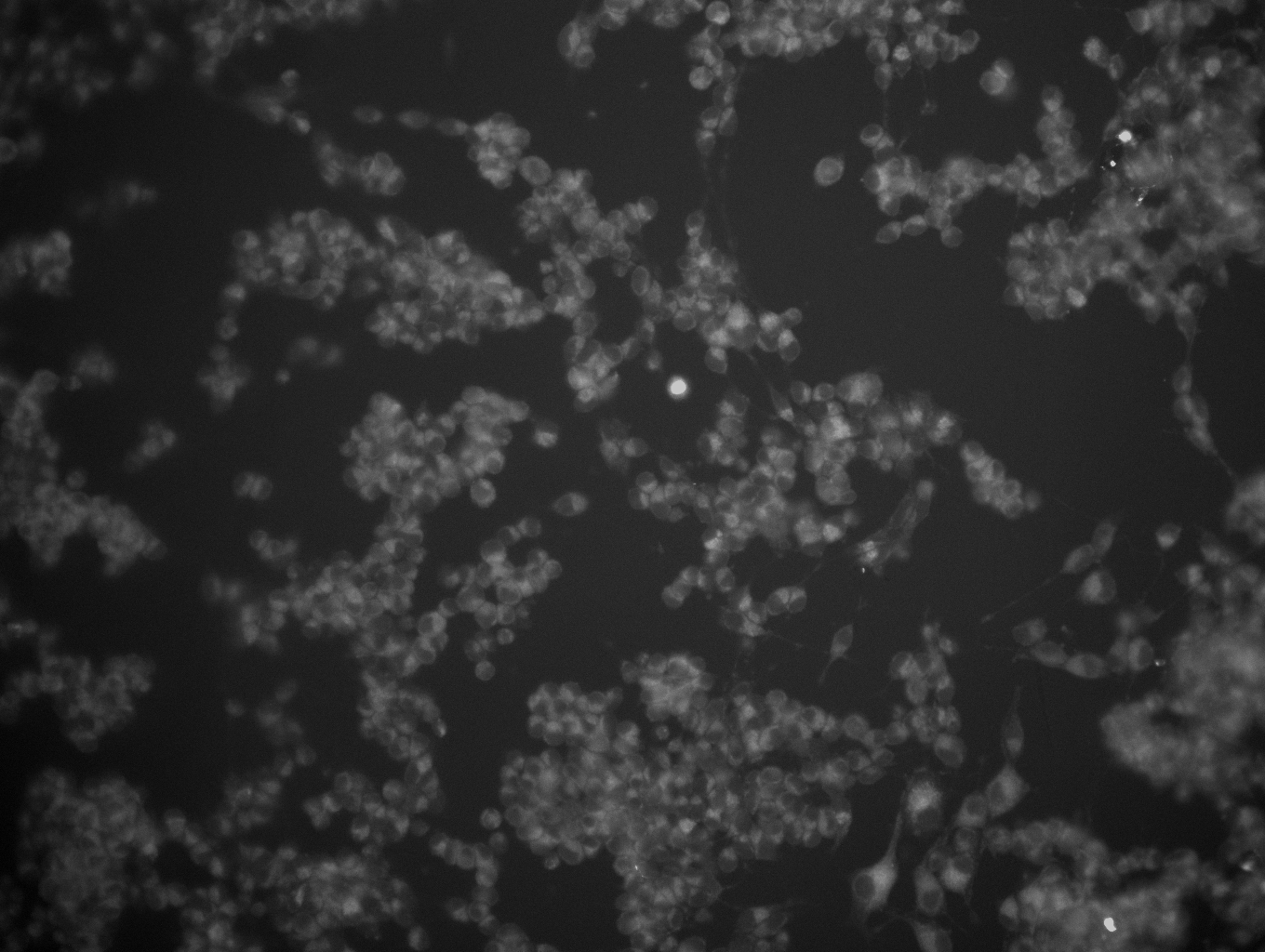

Supplement: Supplementary file 5 — Source data Fig. 4 [file 44321_2025_349_MOESM5_ESM.zip › Source data Figure 4/Fig4E/R1/PCZ Representative photos/10x/PCP_FL20240112060.tif]

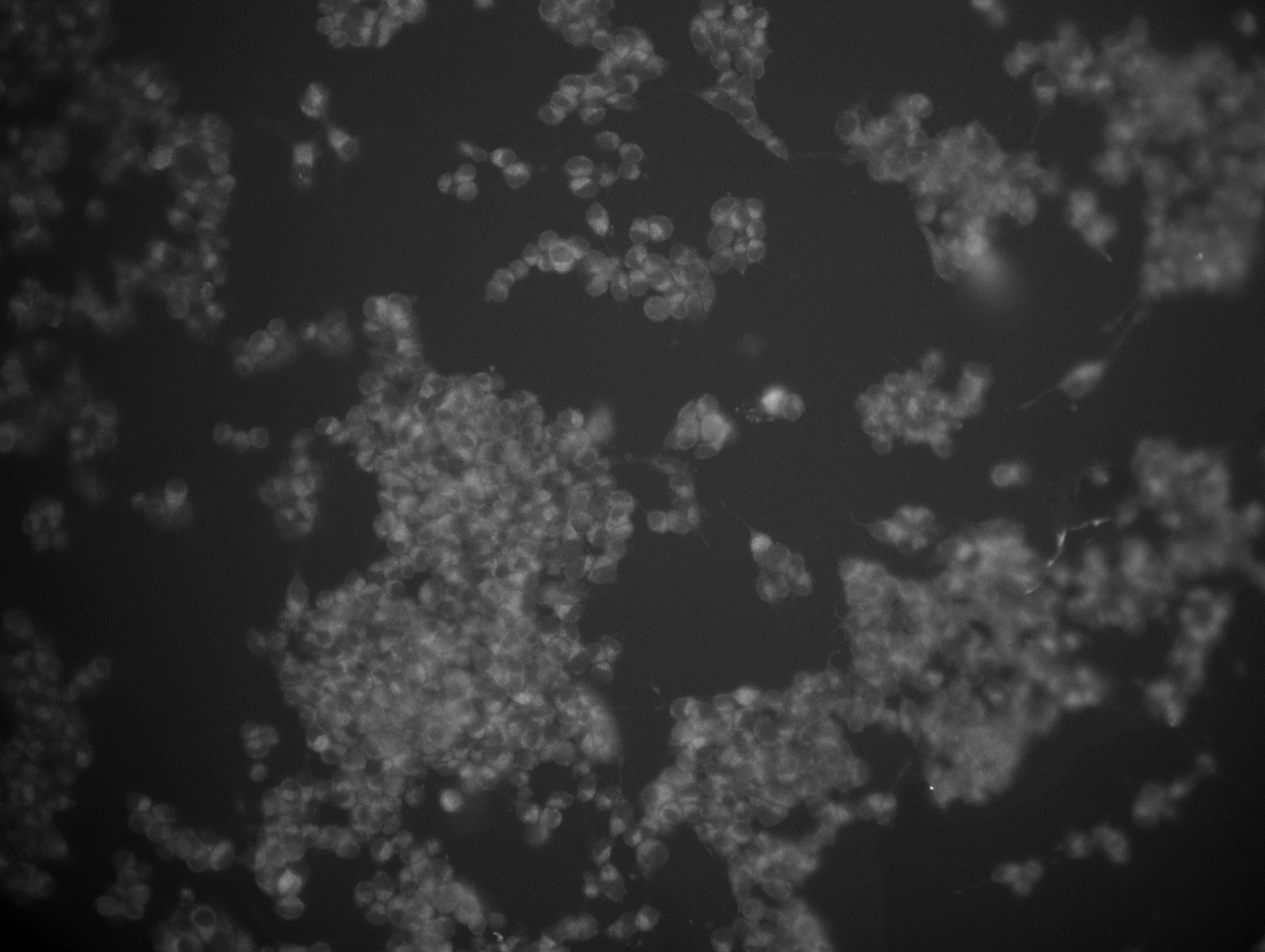

Supplement: Supplementary file 5 — Source data Fig. 4 [file 44321_2025_349_MOESM5_ESM.zip › Source data Figure 4/Fig4E/R1/PCZ Representative photos/10x/PCP_FL20240112061.tif]

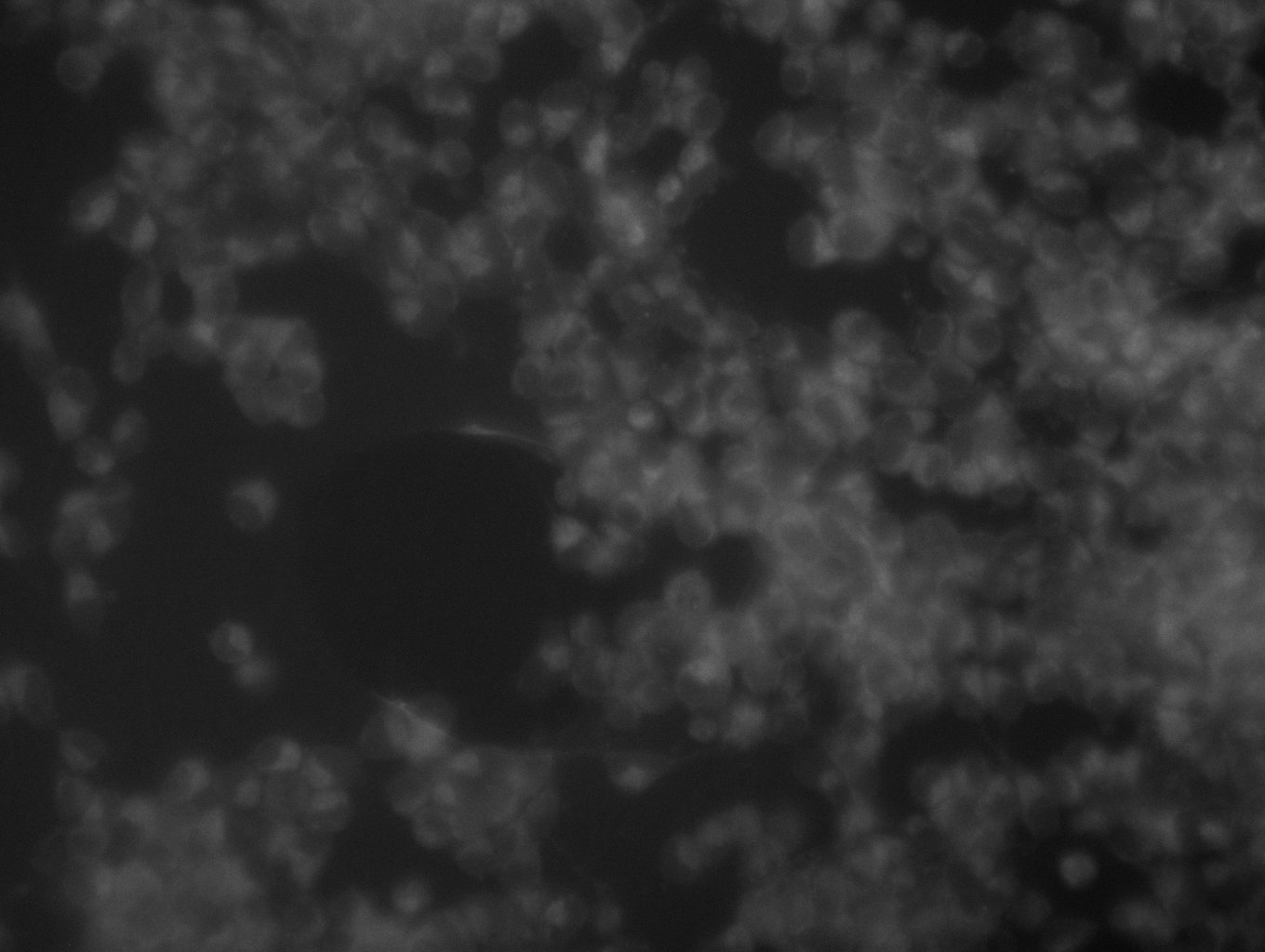

Supplement: Supplementary file 5 — Source data Fig. 4 [file 44321_2025_349_MOESM5_ESM.zip › Source data Figure 4/Fig4E/R1/PCZ Representative photos/20x/PCP_FL20240112065.tif]

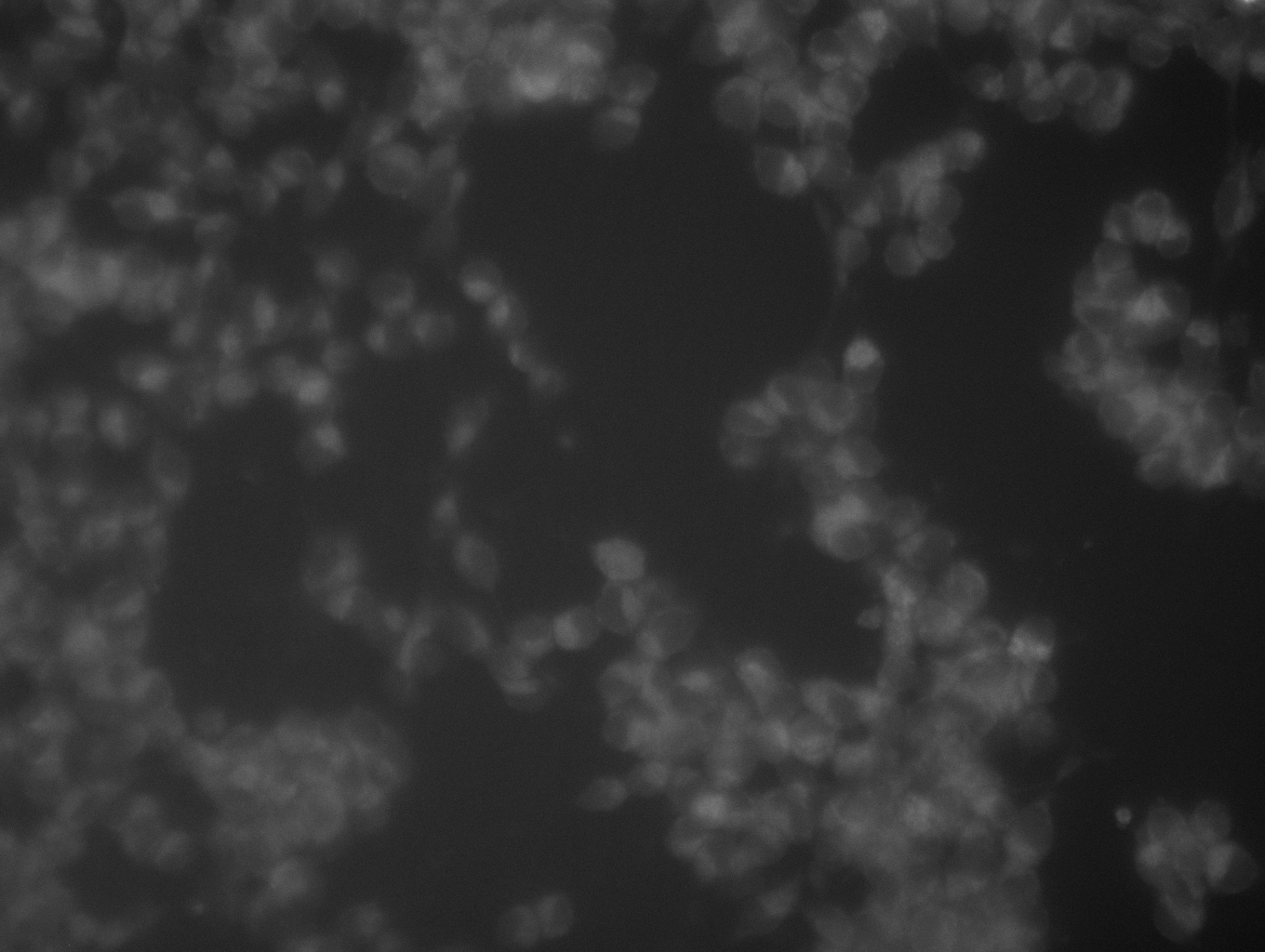

Supplement: Supplementary file 5 — Source data Fig. 4 [file 44321_2025_349_MOESM5_ESM.zip › Source data Figure 4/Fig4E/R1/PCZ Representative photos/20x/PCP_FL20240112067.tif]

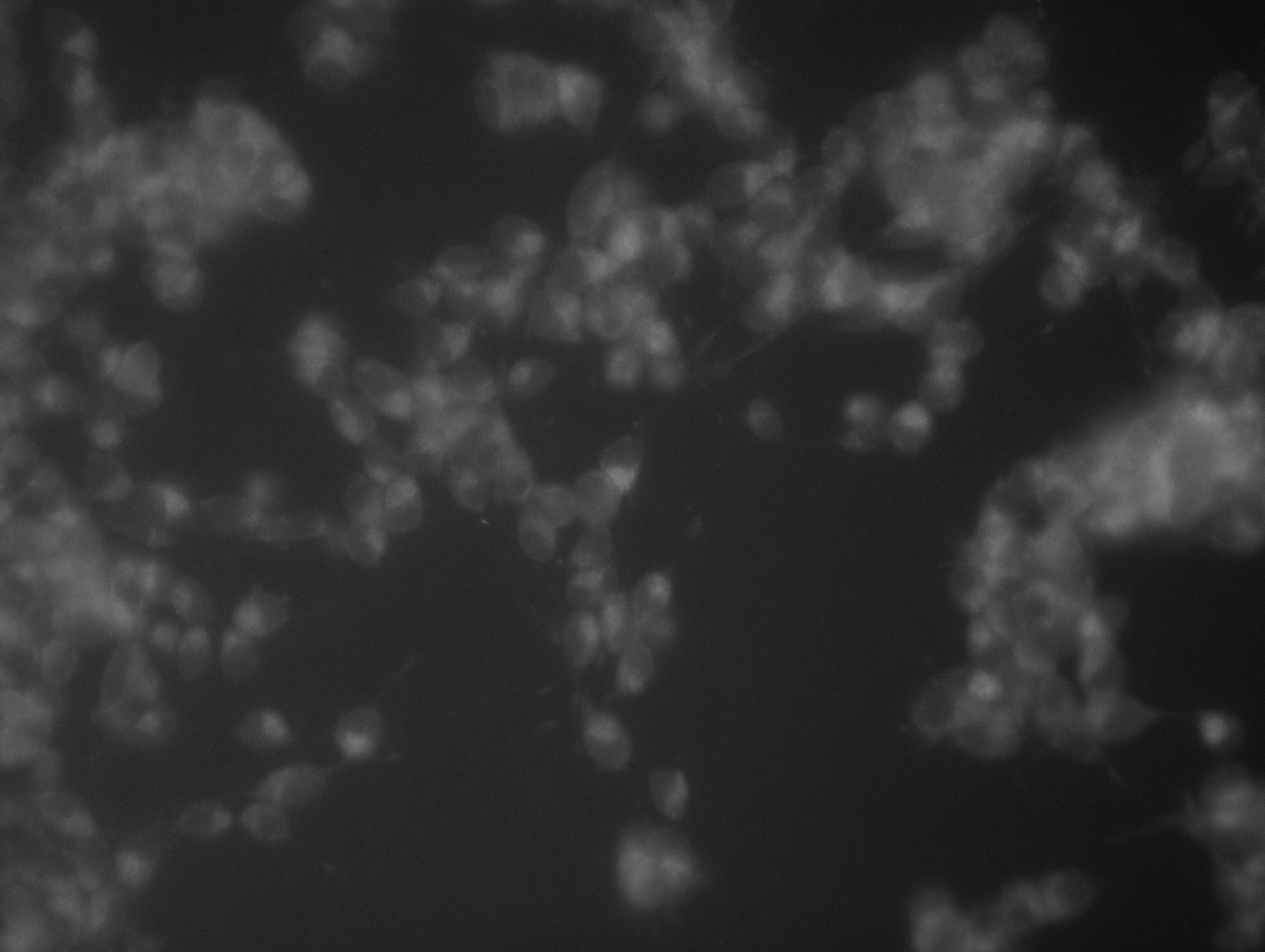

Supplement: Supplementary file 5 — Source data Fig. 4 [file 44321_2025_349_MOESM5_ESM.zip › Source data Figure 4/Fig4E/R1/PCZ Representative photos/20x/PCP_FL20240112069.tif]

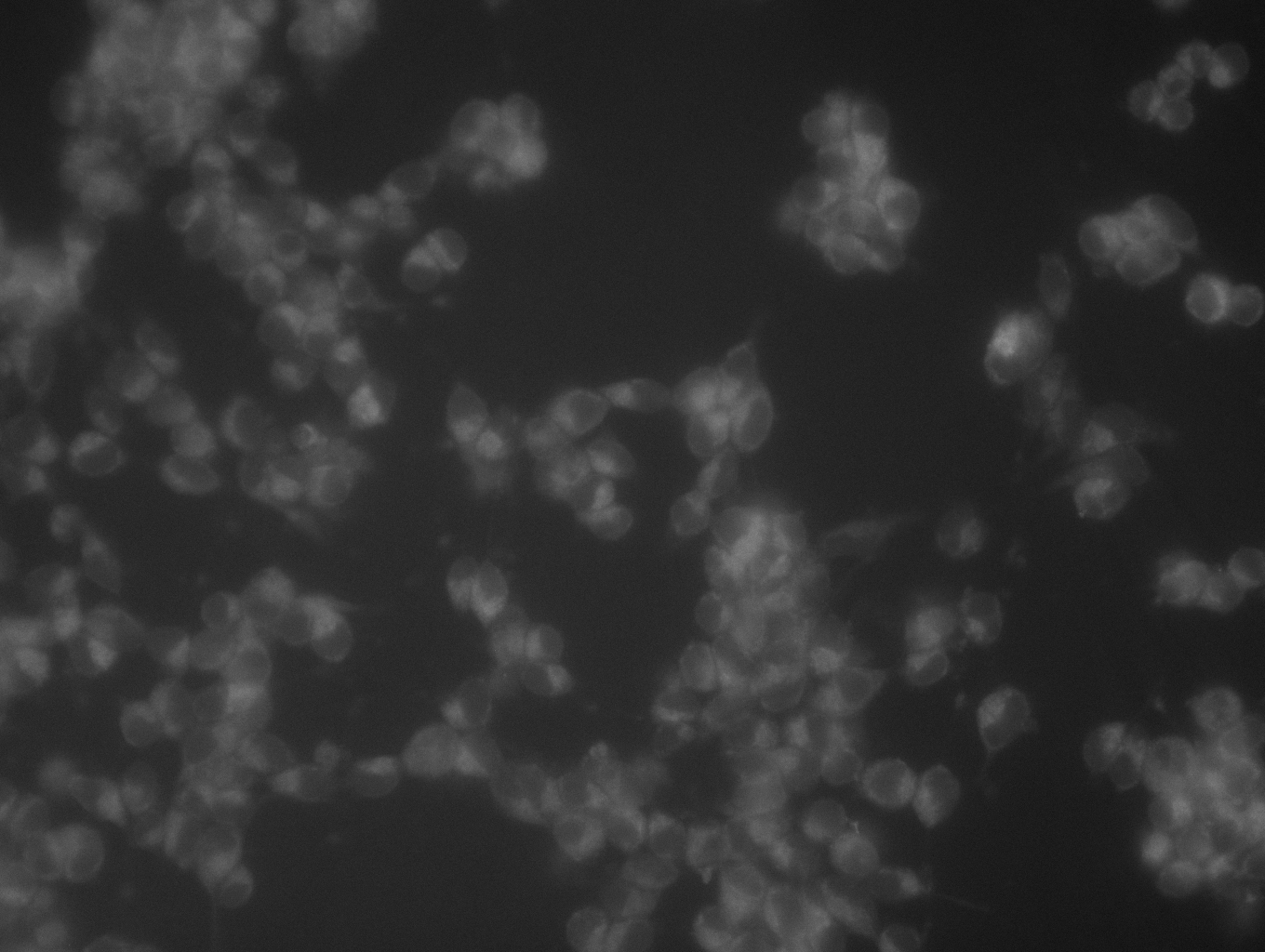

Supplement: Supplementary file 5 — Source data Fig. 4 [file 44321_2025_349_MOESM5_ESM.zip › Source data Figure 4/Fig4E/R1/PCZ Representative photos/20x/PCP_FL20240112074.tif]

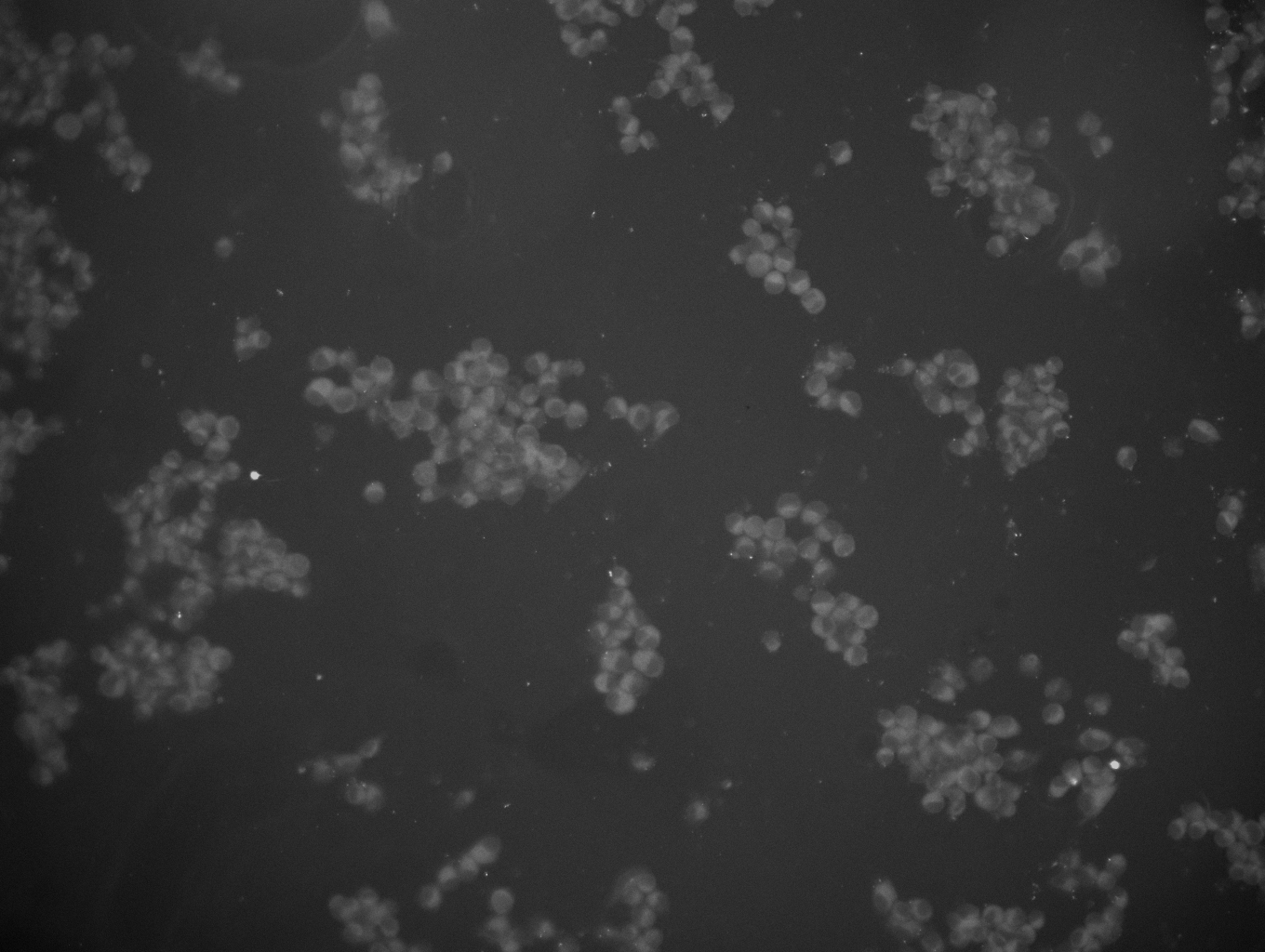

Supplement: Supplementary file 5 — Source data Fig. 4 [file 44321_2025_349_MOESM5_ESM.zip › Source data Figure 4/Fig4E/R1/PIT Representative photos/10x/PIT_FL20240112082.tif]

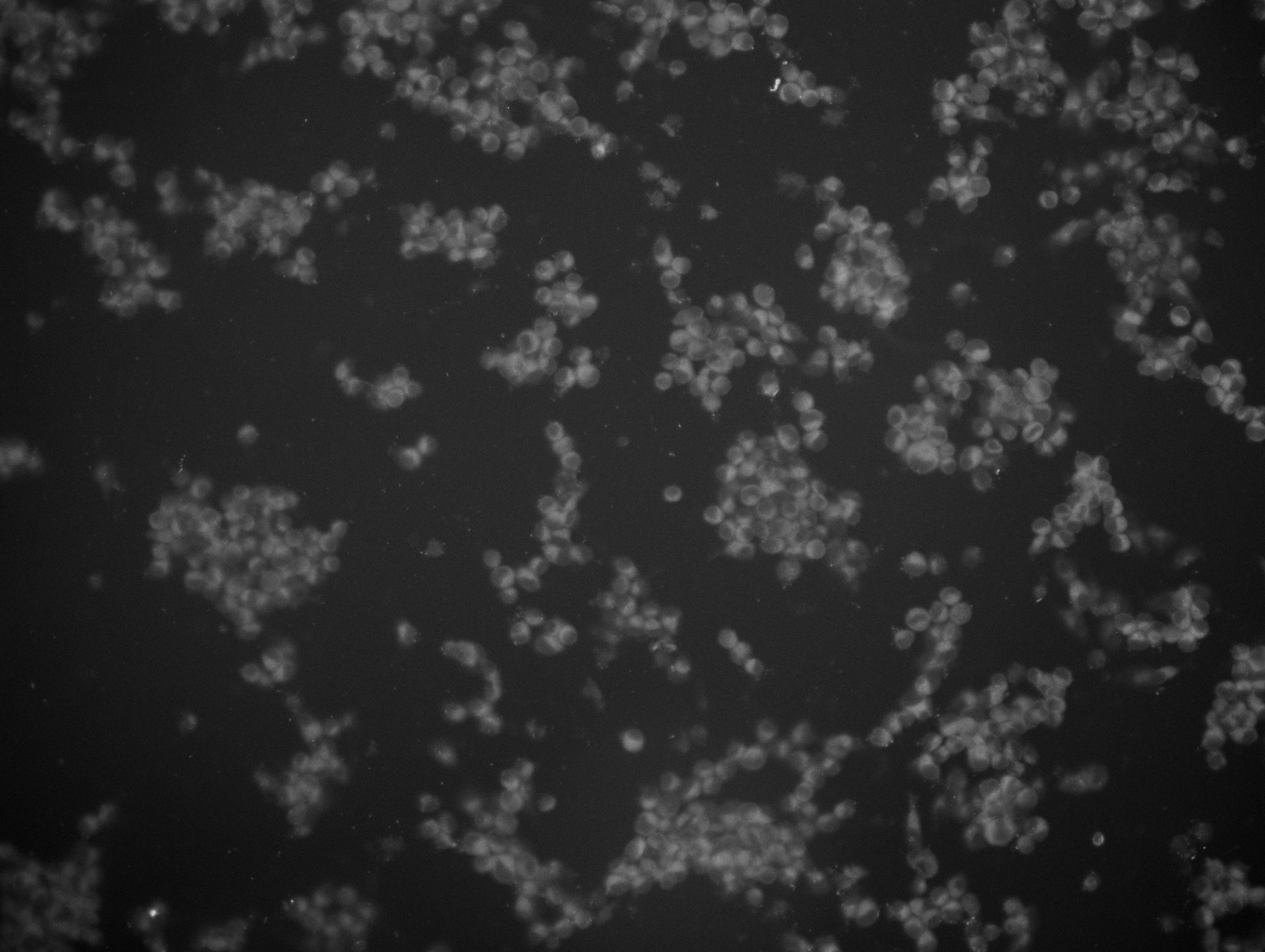

Supplement: Supplementary file 5 — Source data Fig. 4 [file 44321_2025_349_MOESM5_ESM.zip › Source data Figure 4/Fig4E/R1/PIT Representative photos/10x/PIT_FL20240112083.tif]

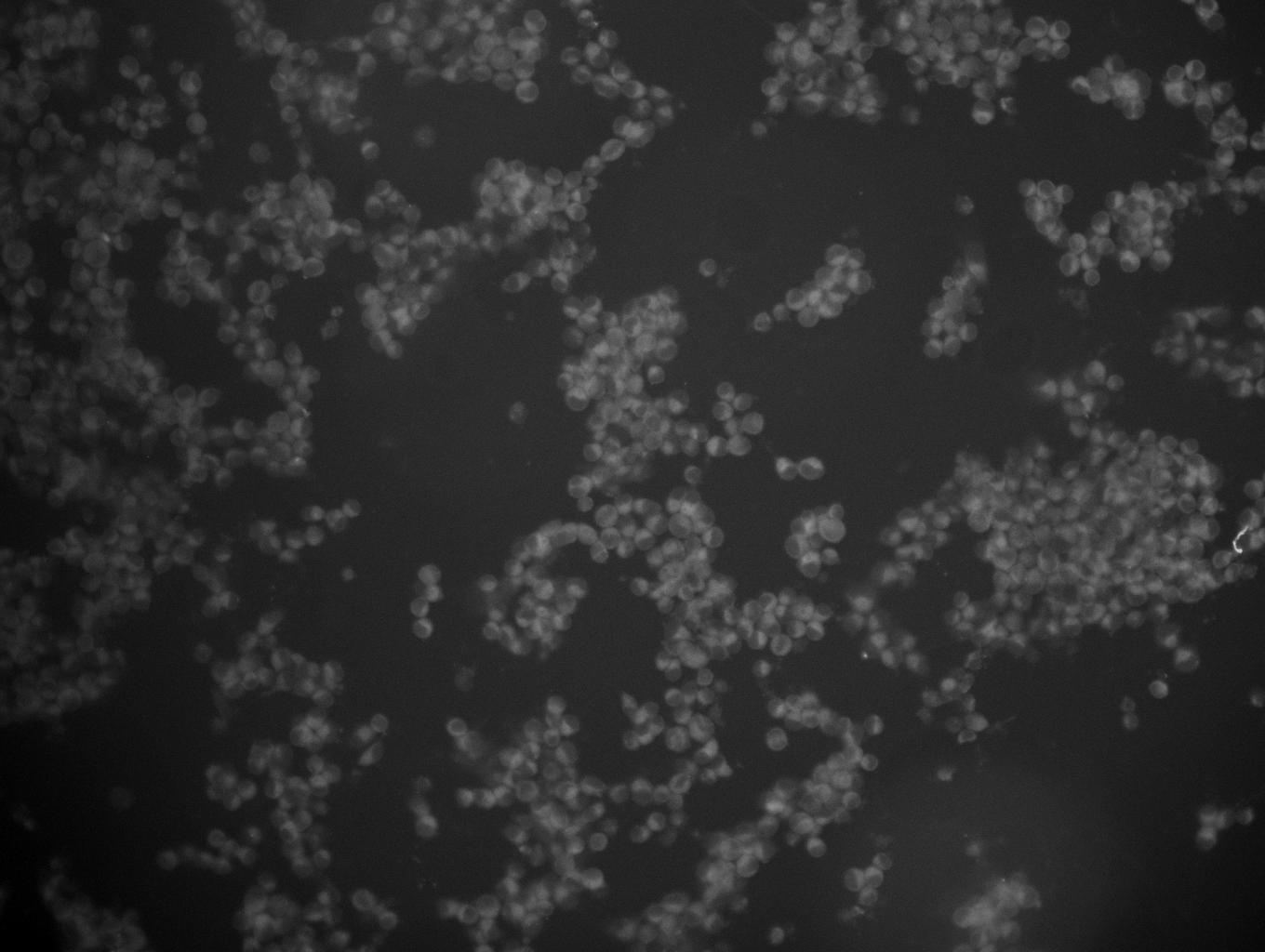

Supplement: Supplementary file 5 — Source data Fig. 4 [file 44321_2025_349_MOESM5_ESM.zip › Source data Figure 4/Fig4E/R1/PIT Representative photos/10x/PIT_FL20240112085.tif]

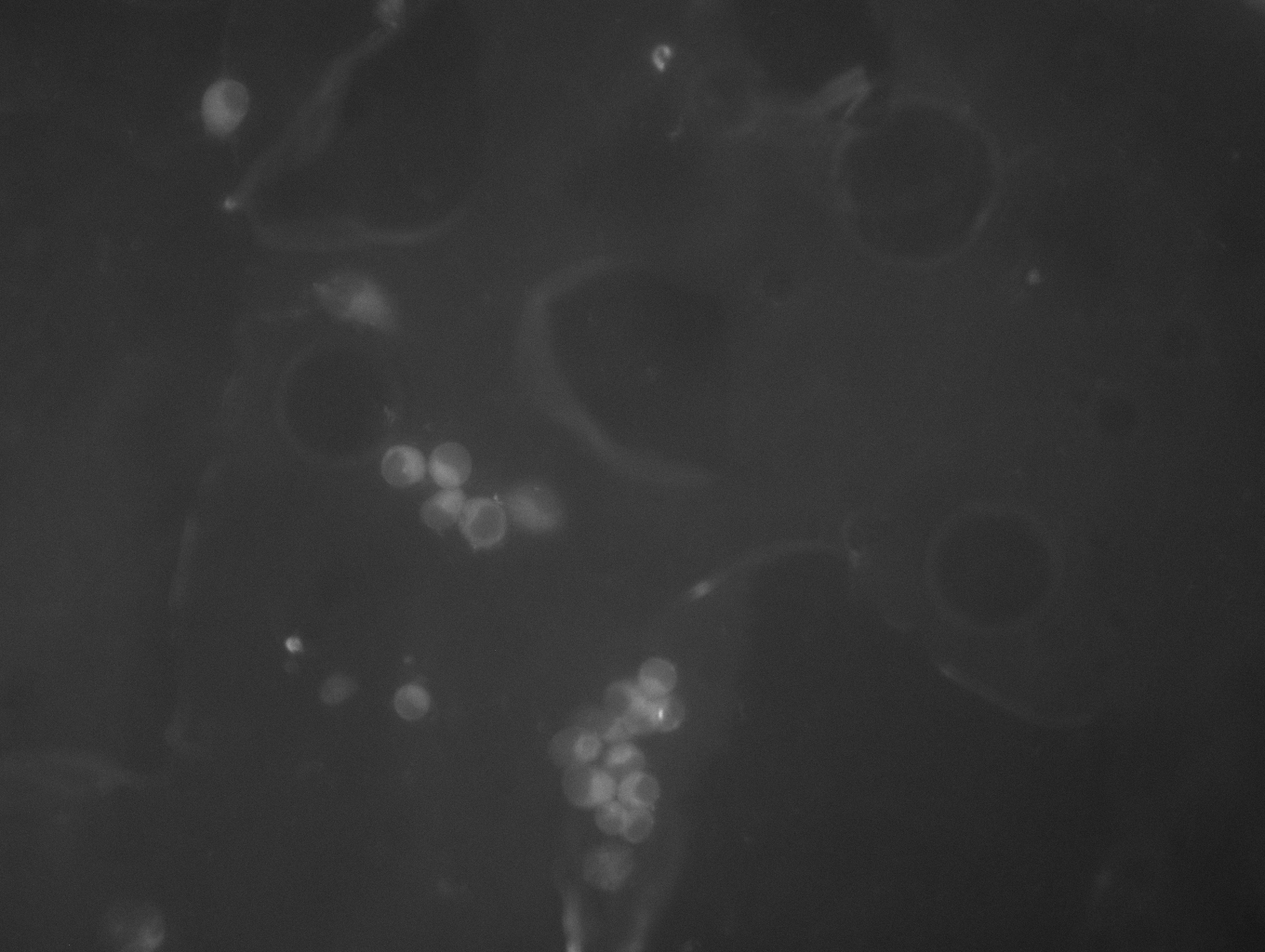

Supplement: Supplementary file 5 — Source data Fig. 4 [file 44321_2025_349_MOESM5_ESM.zip › Source data Figure 4/Fig4E/R1/PIT Representative photos/20x/PIT_FL20240112090.tif]

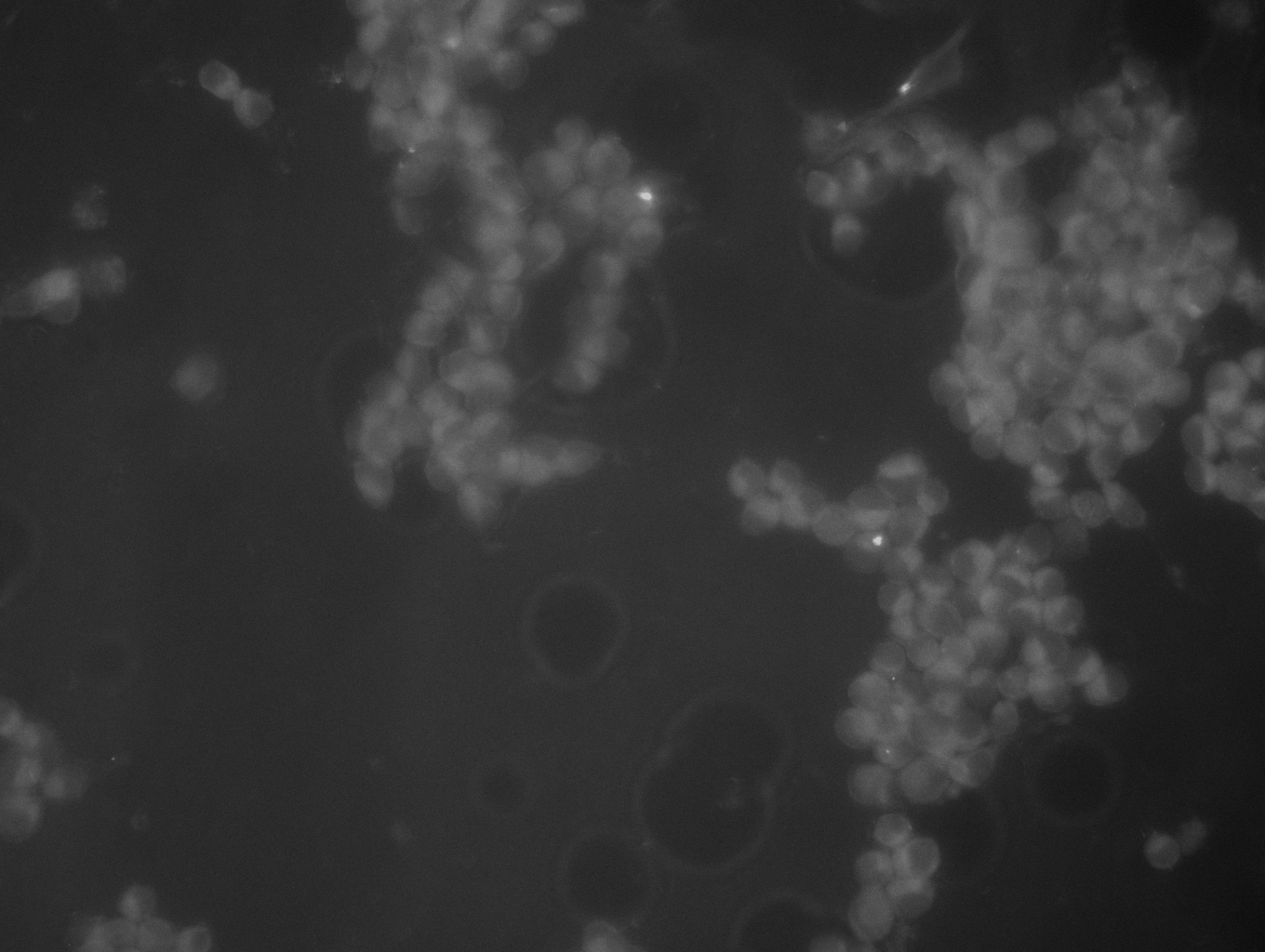

Supplement: Supplementary file 5 — Source data Fig. 4 [file 44321_2025_349_MOESM5_ESM.zip › Source data Figure 4/Fig4E/R1/PIT Representative photos/20x/PIT_FL20240112093.tif]

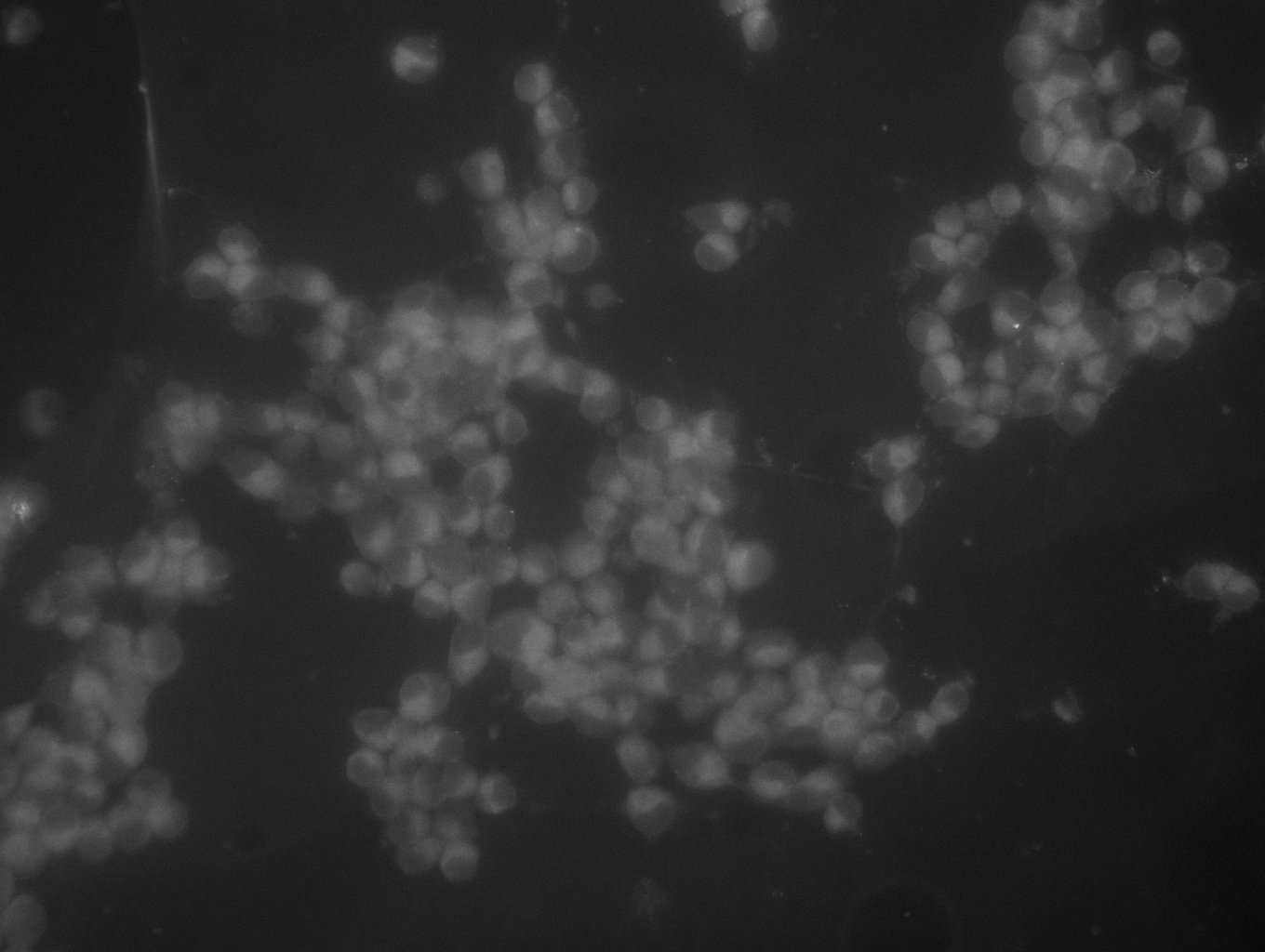

Supplement: Supplementary file 5 — Source data Fig. 4 [file 44321_2025_349_MOESM5_ESM.zip › Source data Figure 4/Fig4E/R1/PIT Representative photos/20x/PIT_FL20240112096.tif]

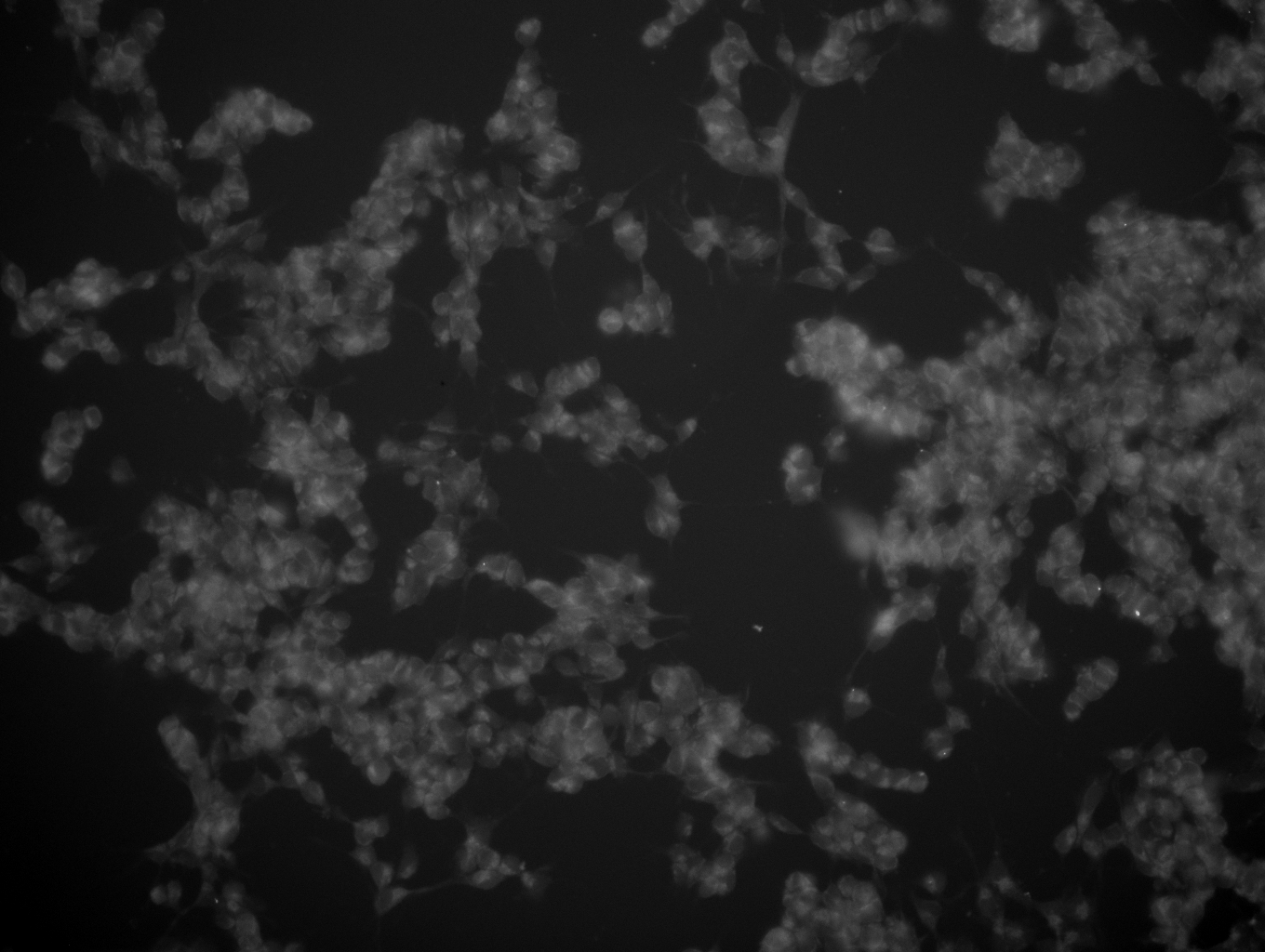

Supplement: Supplementary file 5 — Source data Fig. 4 [file 44321_2025_349_MOESM5_ESM.zip › Source data Figure 4/Fig4E/R2/DMSO Representative photos R2/R2/10x/super_FL20240126254DMSO.tif]

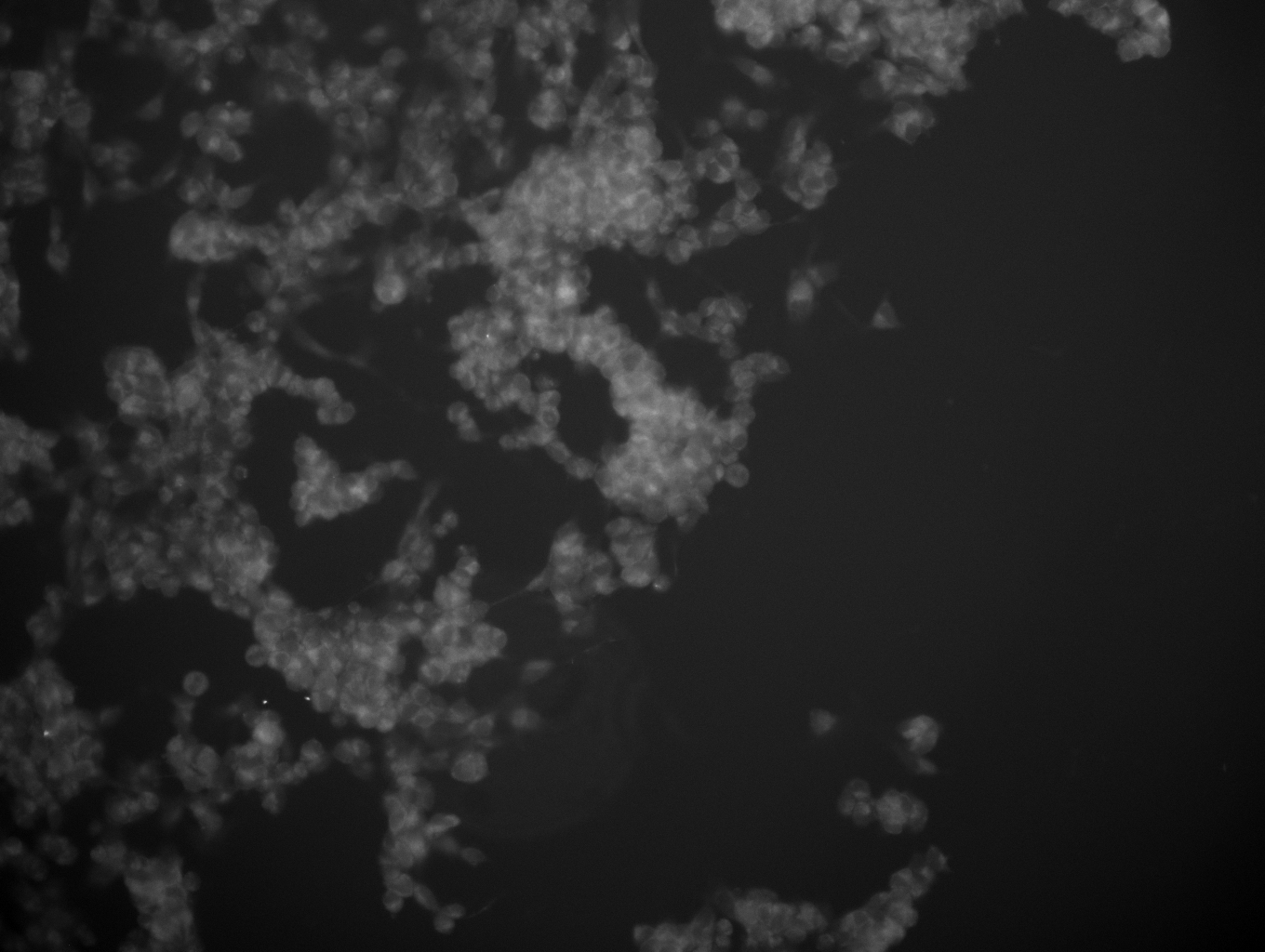

Supplement: Supplementary file 5 — Source data Fig. 4 [file 44321_2025_349_MOESM5_ESM.zip › Source data Figure 4/Fig4E/R2/DMSO Representative photos R2/R2/10x/super_FL20240126262DMSO.tif]

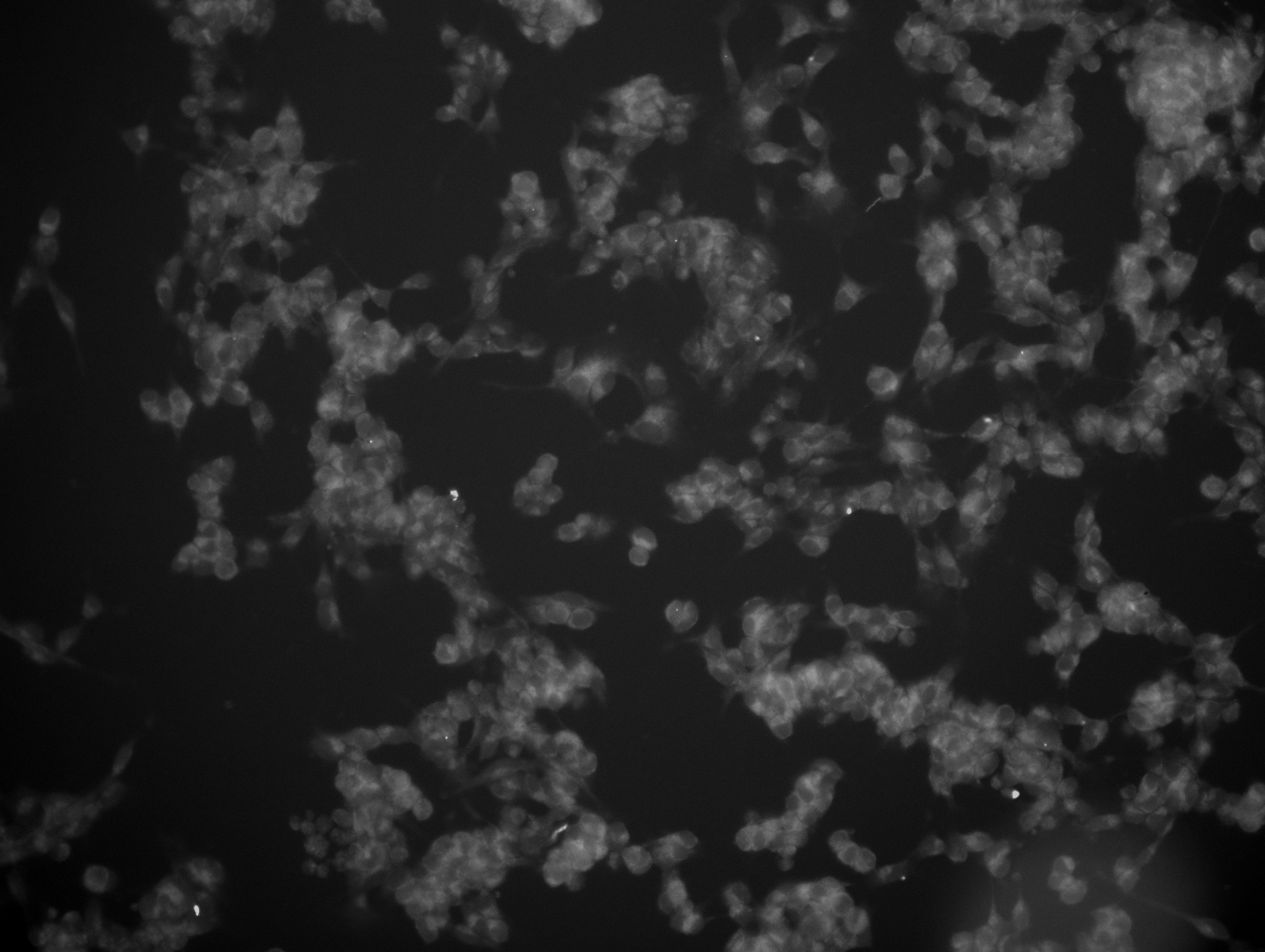

Supplement: Supplementary file 5 — Source data Fig. 4 [file 44321_2025_349_MOESM5_ESM.zip › Source data Figure 4/Fig4E/R2/DMSO Representative photos R2/R2/10x/super_FL20240126266DMSO.tif]

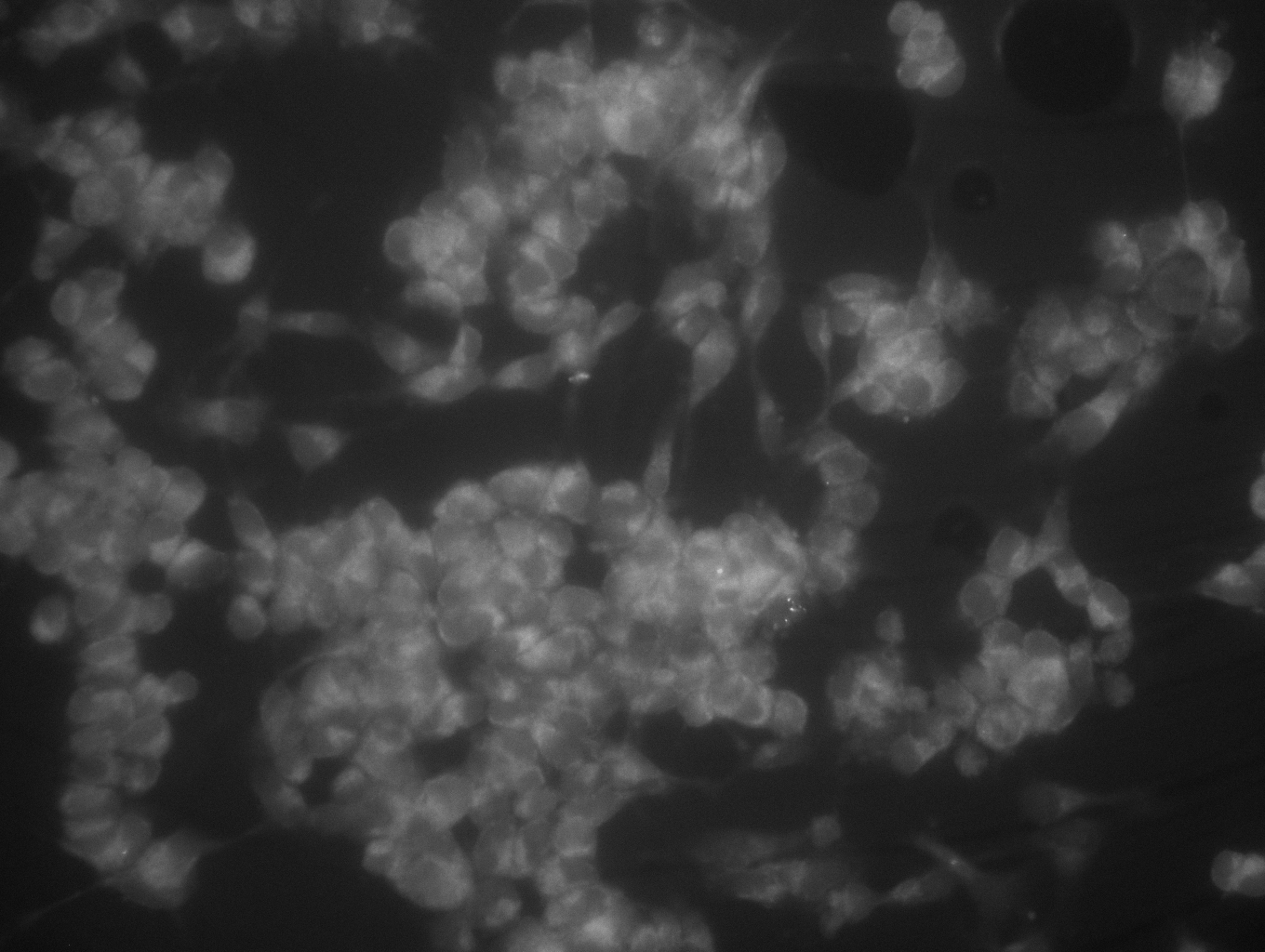

Supplement: Supplementary file 5 — Source data Fig. 4 [file 44321_2025_349_MOESM5_ESM.zip › Source data Figure 4/Fig4E/R2/DMSO Representative photos R2/R2/20x/super_FL20240126263DMSO.tif]

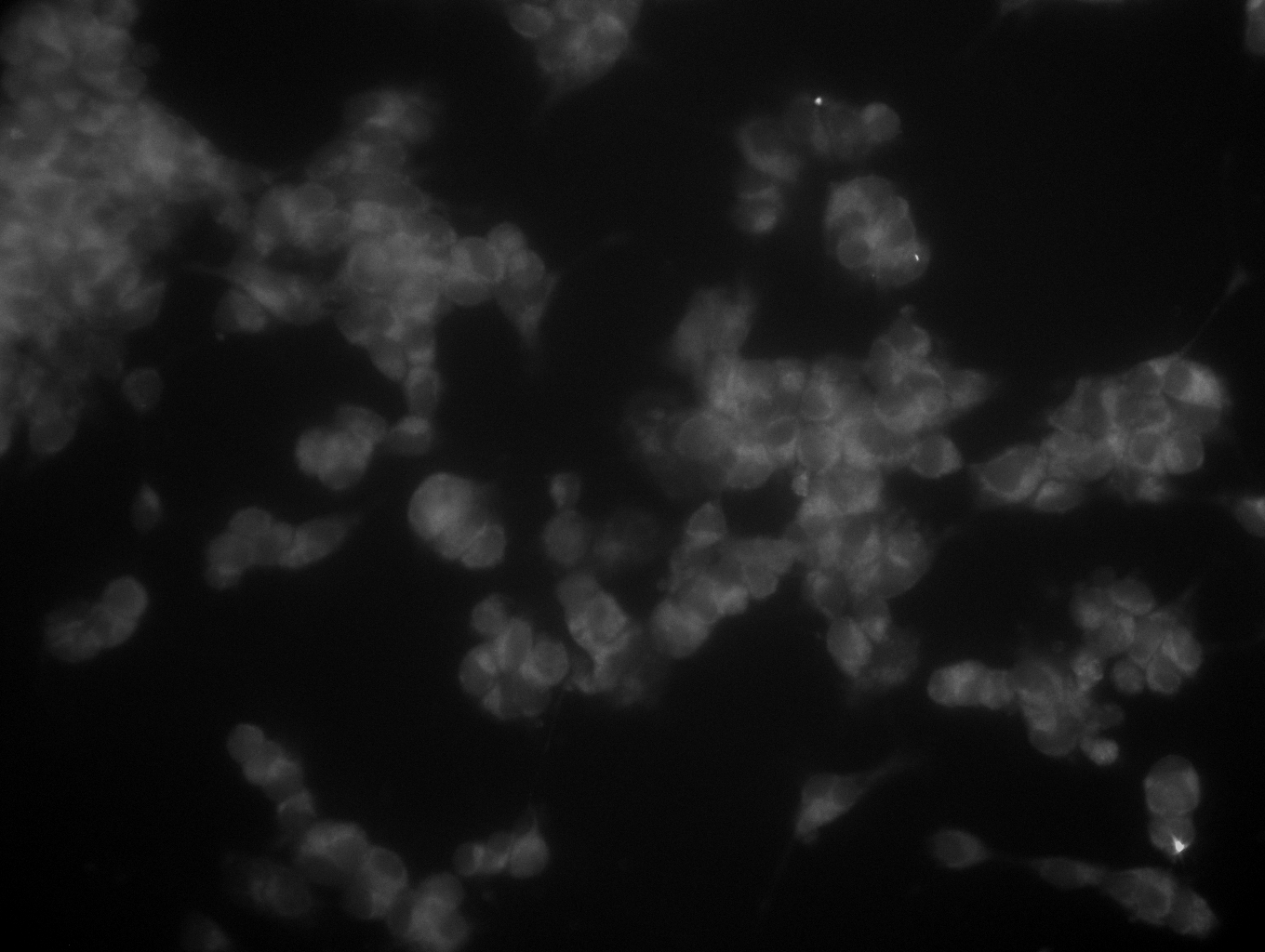

Supplement: Supplementary file 5 — Source data Fig. 4 [file 44321_2025_349_MOESM5_ESM.zip › Source data Figure 4/Fig4E/R2/DMSO Representative photos R2/R2/20x/super_FL20240126265DMSO.tif]

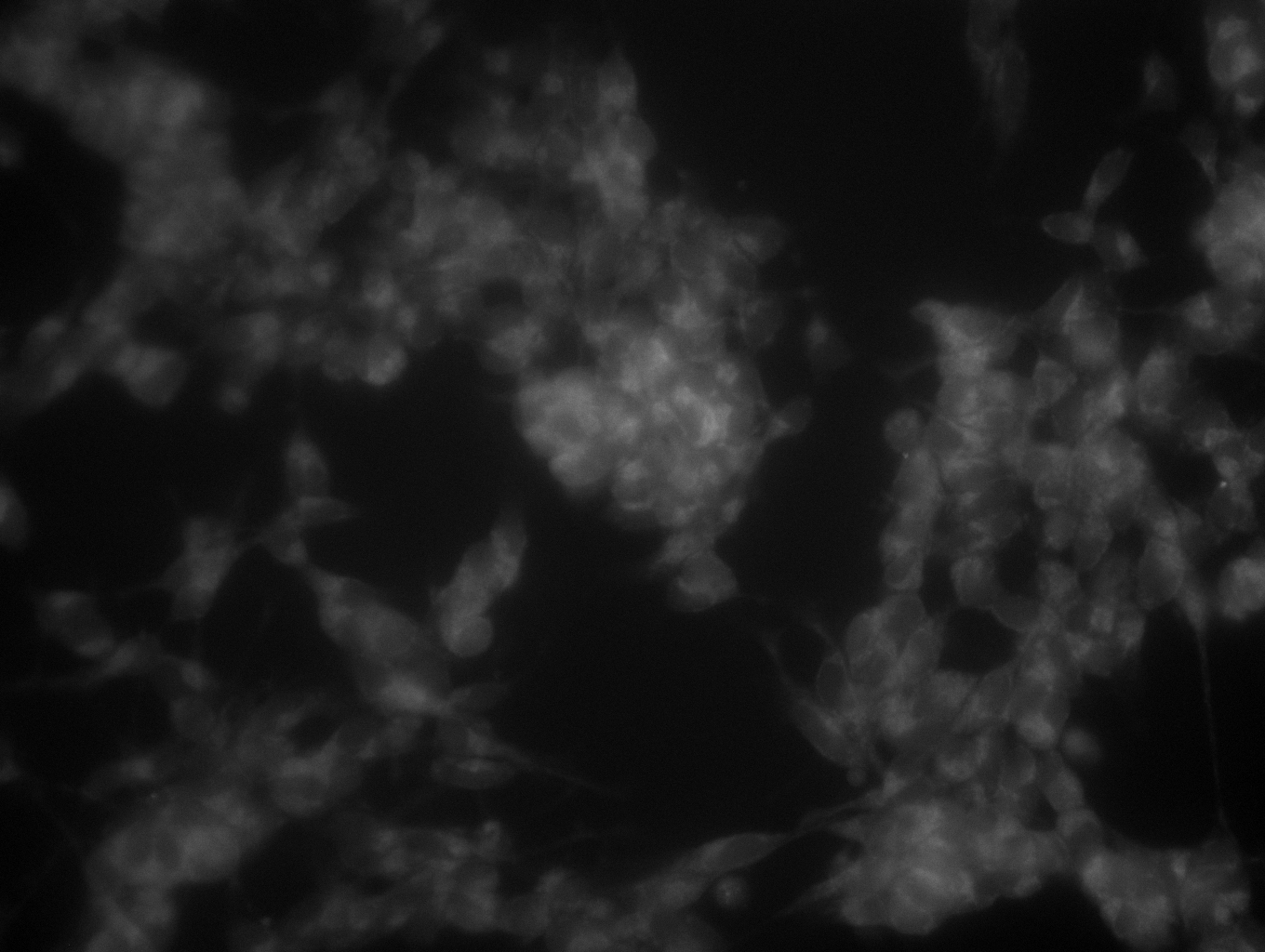

Supplement: Supplementary file 5 — Source data Fig. 4 [file 44321_2025_349_MOESM5_ESM.zip › Source data Figure 4/Fig4E/R2/DMSO Representative photos R2/R2/20x/super_FL20240126273DMSO.tif]

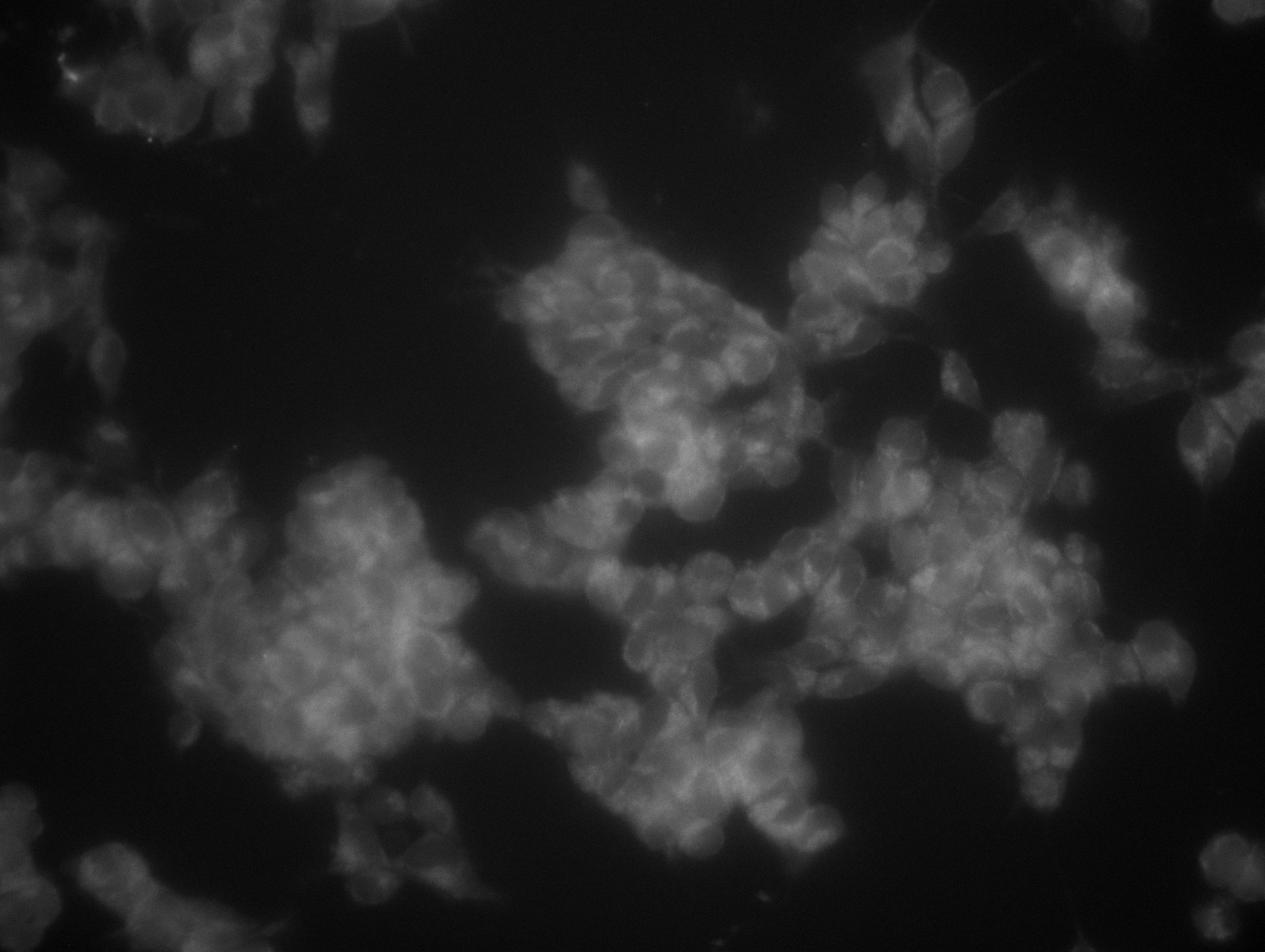

Supplement: Supplementary file 5 — Source data Fig. 4 [file 44321_2025_349_MOESM5_ESM.zip › Source data Figure 4/Fig4E/R2/DMSO Representative photos R2/R2/20x/super_FL20240126275DMSO.tif]

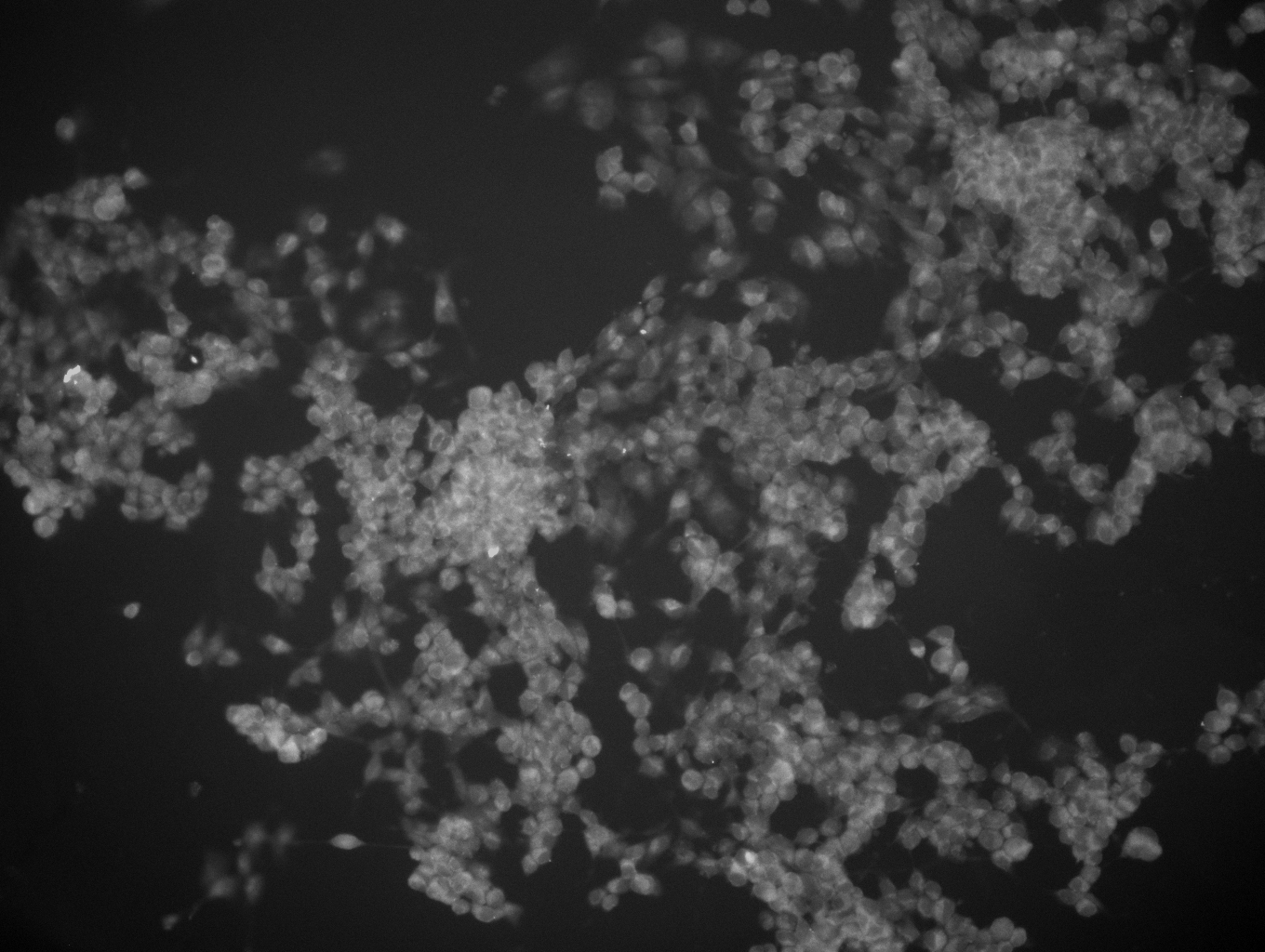

Supplement: Supplementary file 5 — Source data Fig. 4 [file 44321_2025_349_MOESM5_ESM.zip › Source data Figure 4/Fig4E/R2/PCZ Representative photos R2/10x/super_FL20240126PCP333.tif]

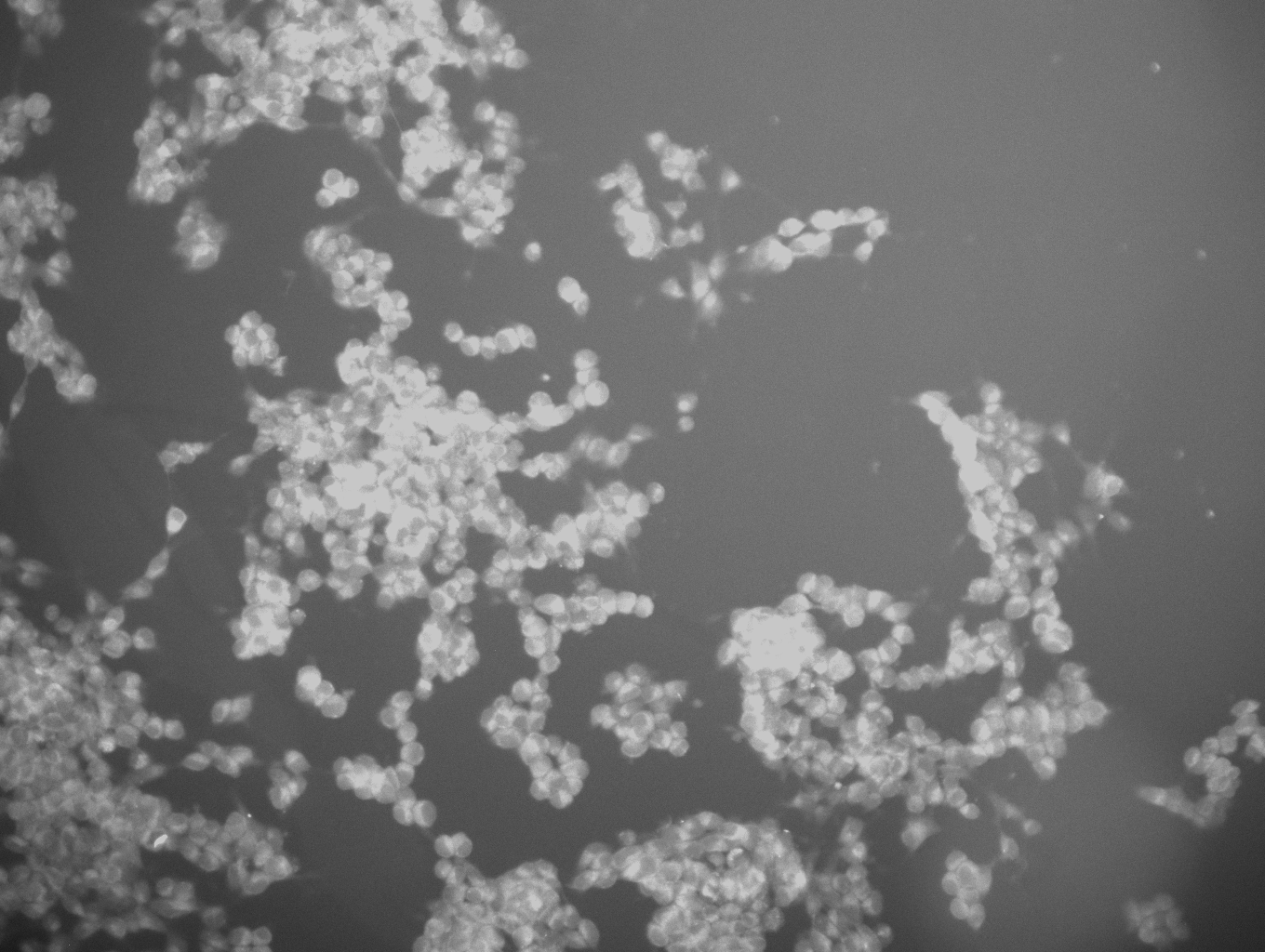

Supplement: Supplementary file 5 — Source data Fig. 4 [file 44321_2025_349_MOESM5_ESM.zip › Source data Figure 4/Fig4E/R2/PCZ Representative photos R2/10x/super_FL20240126PCP335.tif]

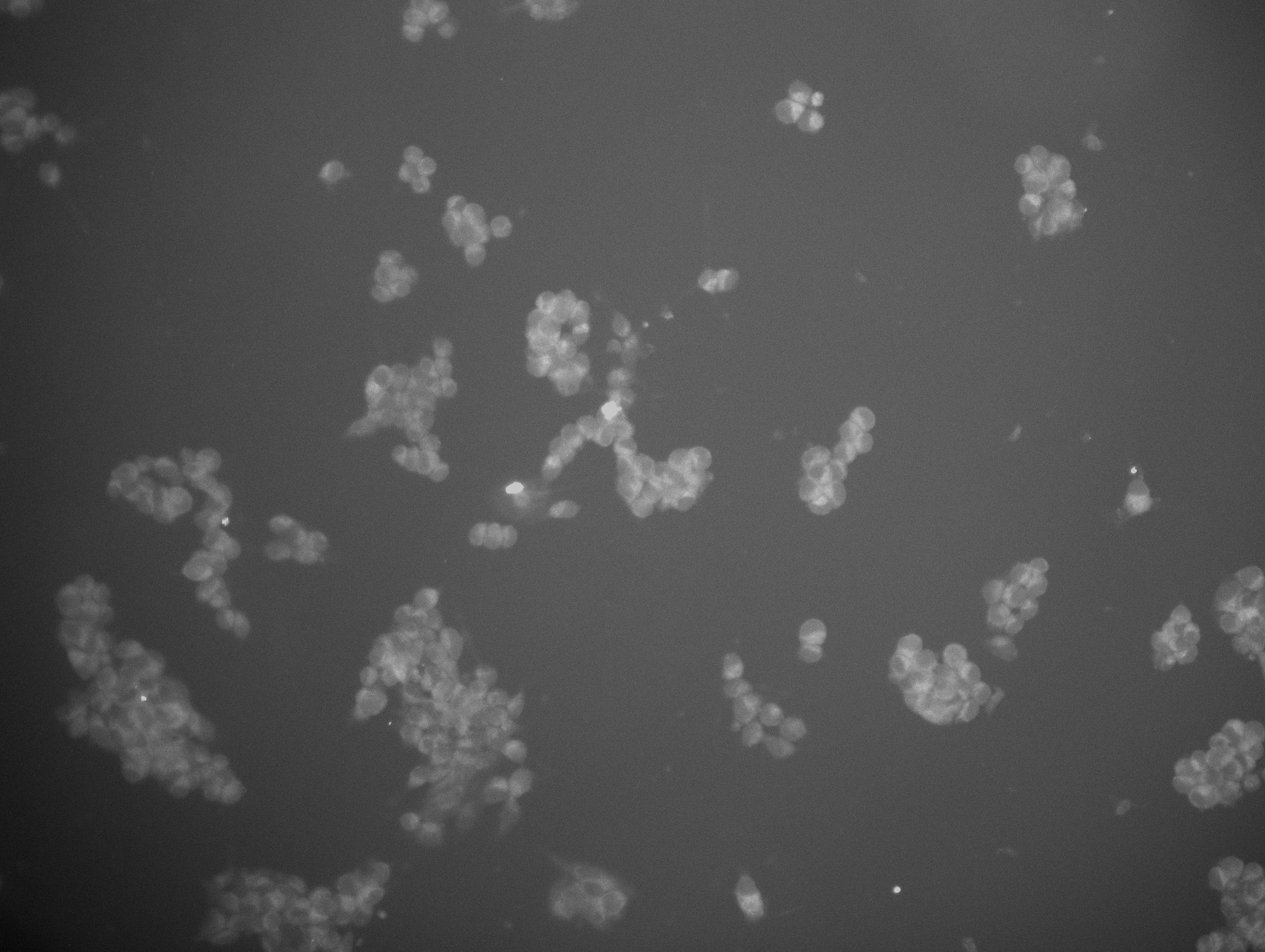

Supplement: Supplementary file 5 — Source data Fig. 4 [file 44321_2025_349_MOESM5_ESM.zip › Source data Figure 4/Fig4E/R2/PCZ Representative photos R2/10x/super_FL20240126PCP347.tif]

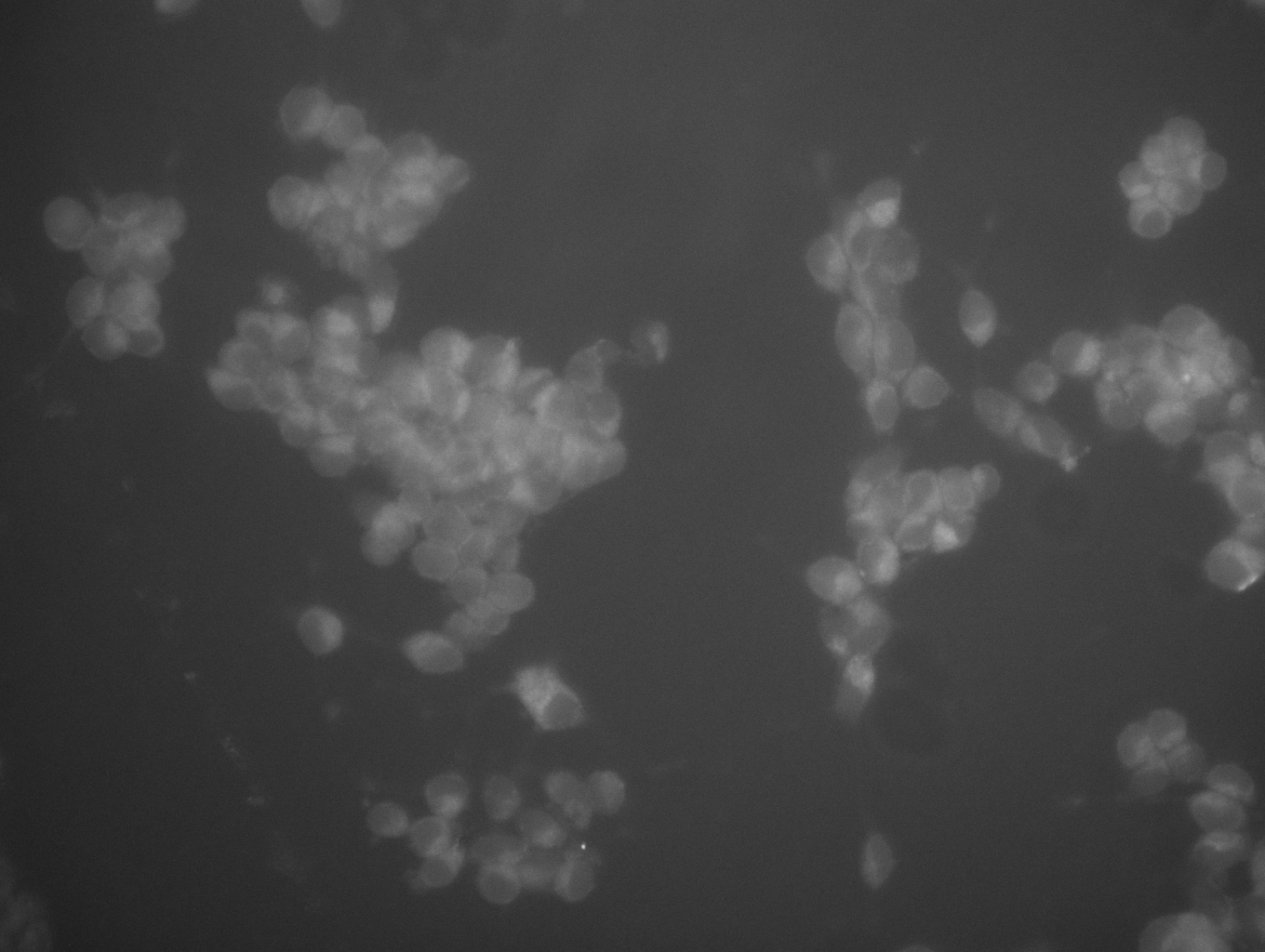

Supplement: Supplementary file 5 — Source data Fig. 4 [file 44321_2025_349_MOESM5_ESM.zip › Source data Figure 4/Fig4E/R2/PCZ Representative photos R2/20x/super_FL20240126PCP348.tif]

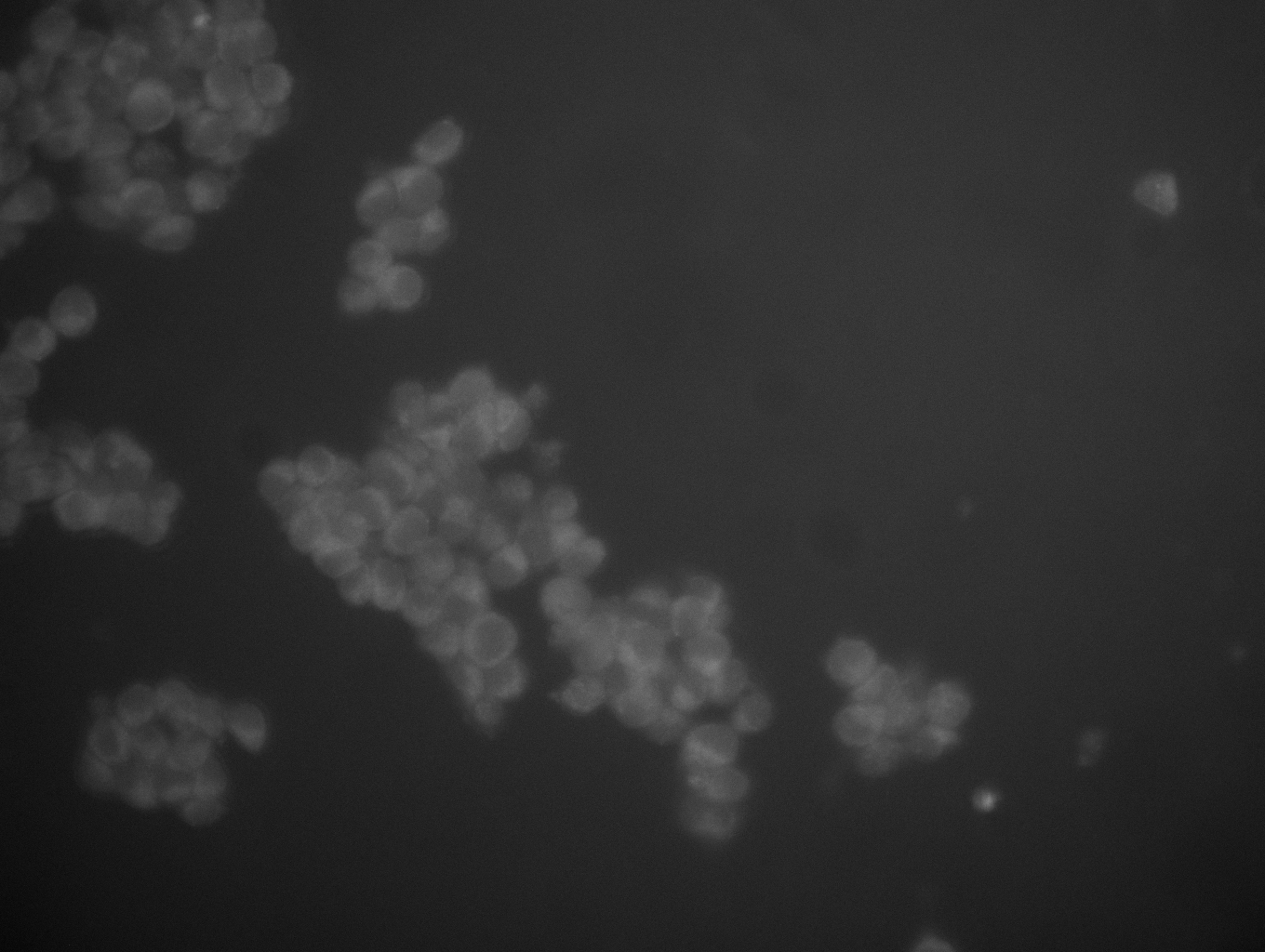

Supplement: Supplementary file 5 — Source data Fig. 4 [file 44321_2025_349_MOESM5_ESM.zip › Source data Figure 4/Fig4E/R2/PCZ Representative photos R2/20x/super_FL20240126PCP352.tif]

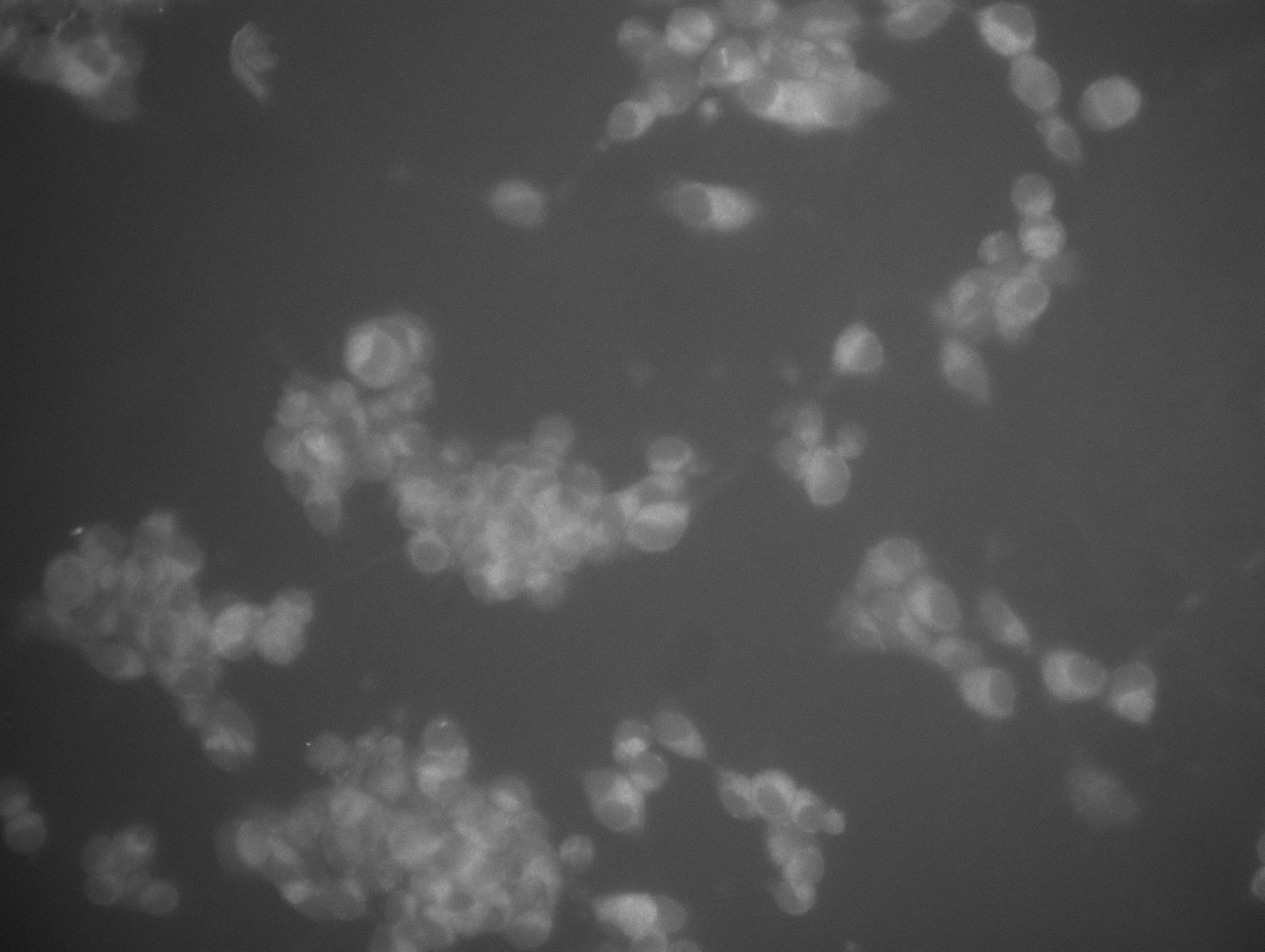

Supplement: Supplementary file 5 — Source data Fig. 4 [file 44321_2025_349_MOESM5_ESM.zip › Source data Figure 4/Fig4E/R2/PCZ Representative photos R2/20x/super_FL20240126PCP360.tif]

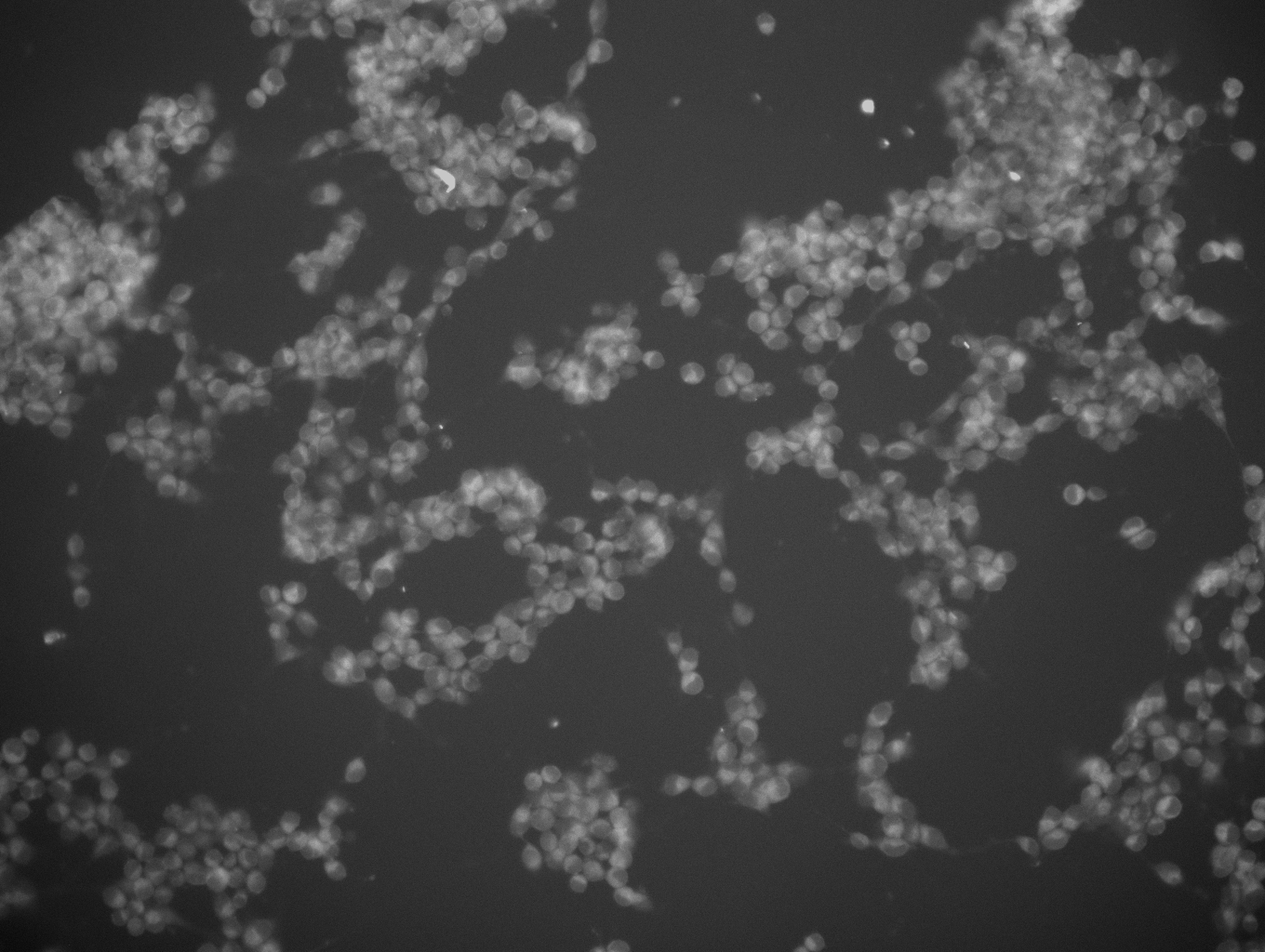

Supplement: Supplementary file 5 — Source data Fig. 4 [file 44321_2025_349_MOESM5_ESM.zip › Source data Figure 4/Fig4E/R2/PIT Representative photos R2/10x/super_FL20240126PIT366.tif]

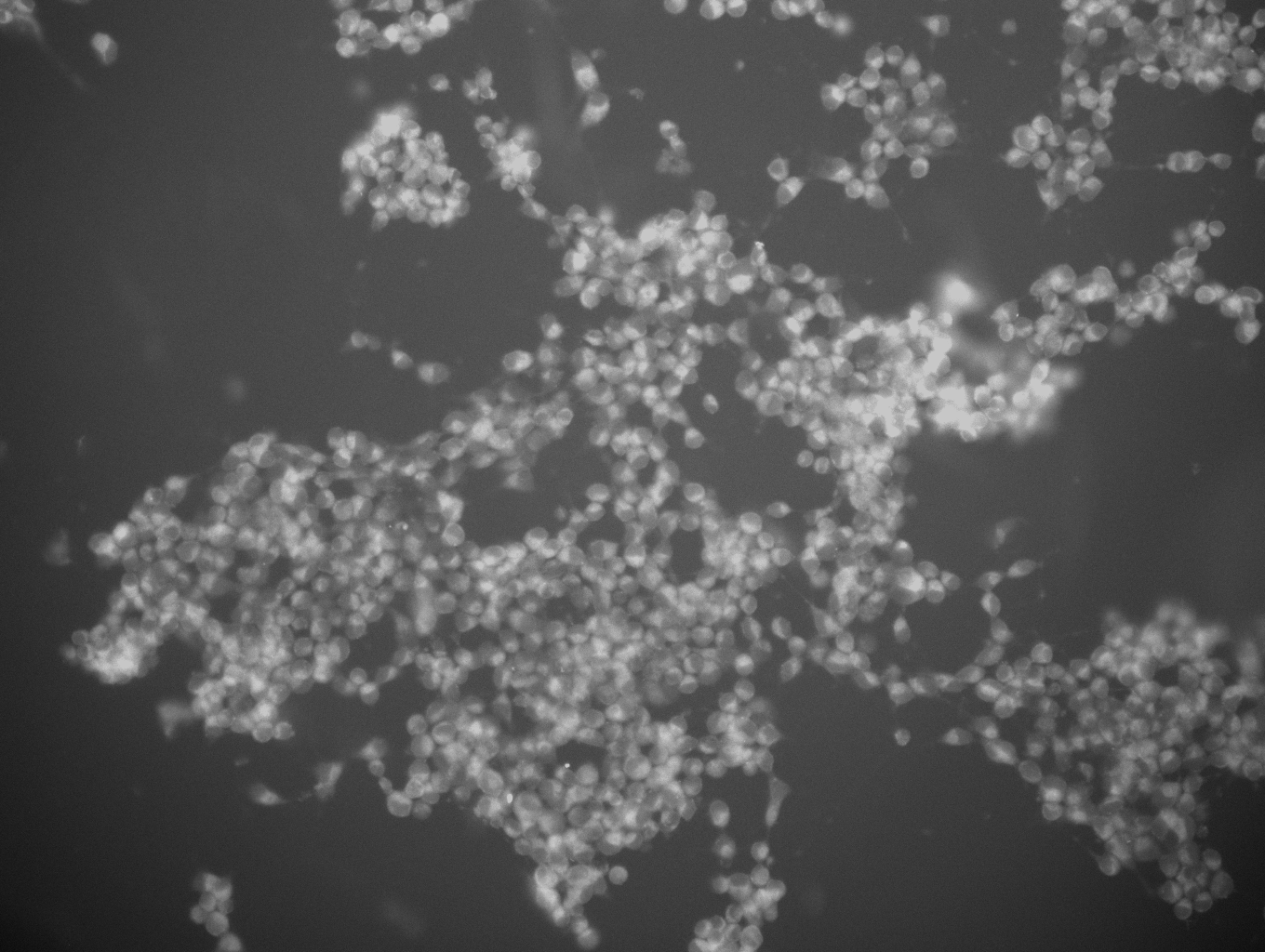

Supplement: Supplementary file 5 — Source data Fig. 4 [file 44321_2025_349_MOESM5_ESM.zip › Source data Figure 4/Fig4E/R2/PIT Representative photos R2/10x/super_FL20240126PIT370.tif]

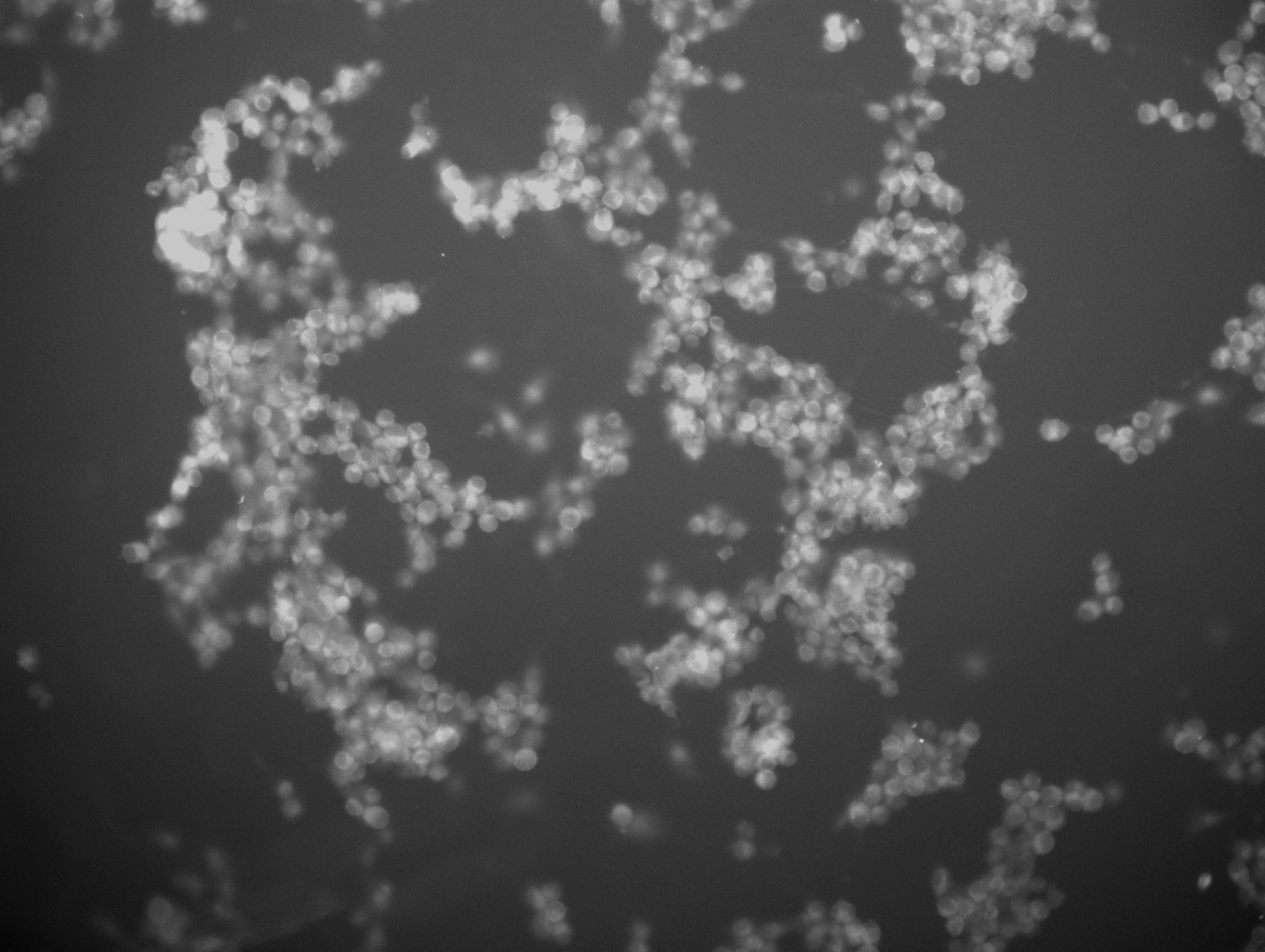

Supplement: Supplementary file 5 — Source data Fig. 4 [file 44321_2025_349_MOESM5_ESM.zip › Source data Figure 4/Fig4E/R2/PIT Representative photos R2/10x/super_FL20240126PIT374.tif]

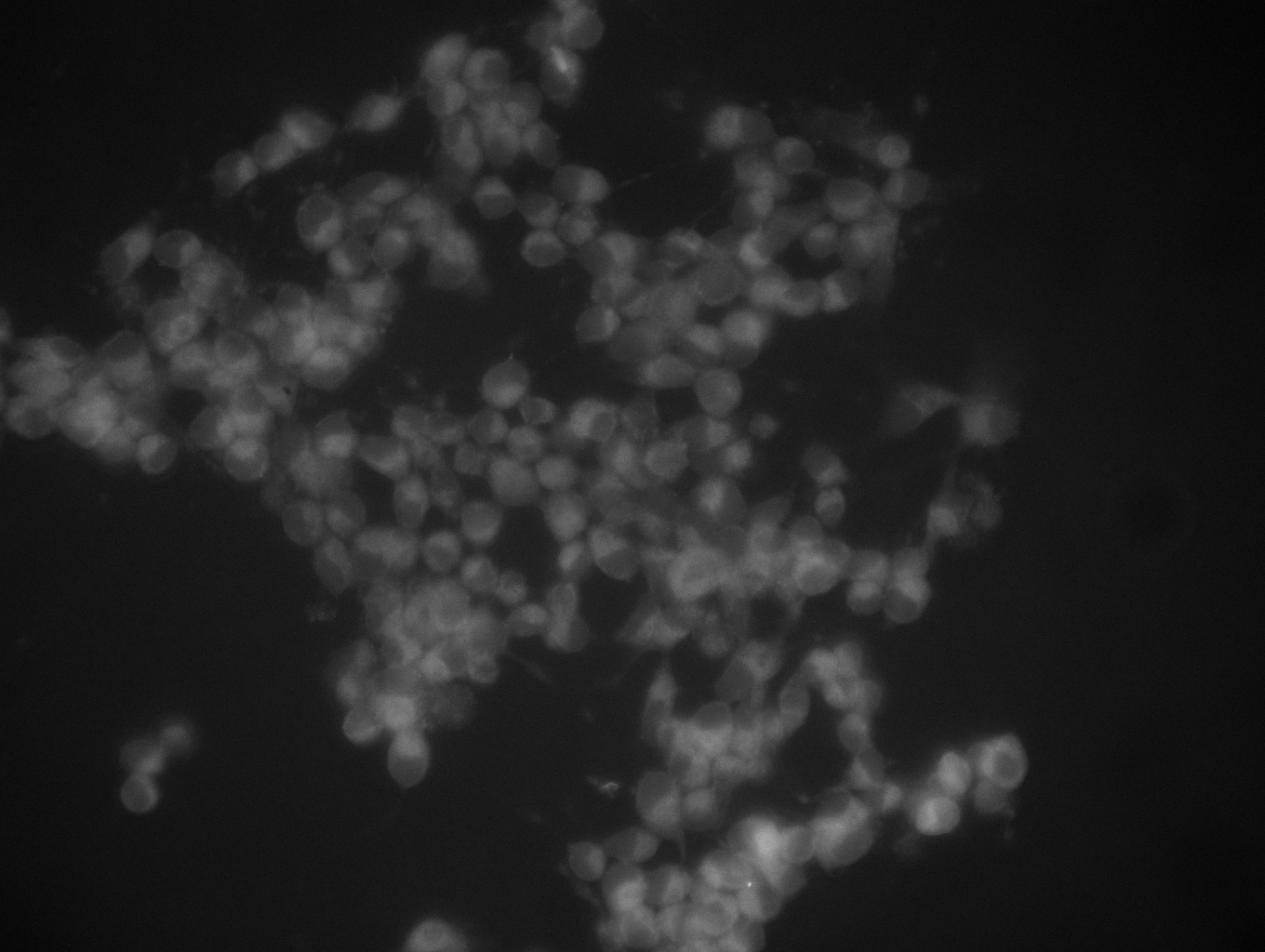

Supplement: Supplementary file 5 — Source data Fig. 4 [file 44321_2025_349_MOESM5_ESM.zip › Source data Figure 4/Fig4E/R2/PIT Representative photos R2/20x/super_FL20240126PIT390.tif]

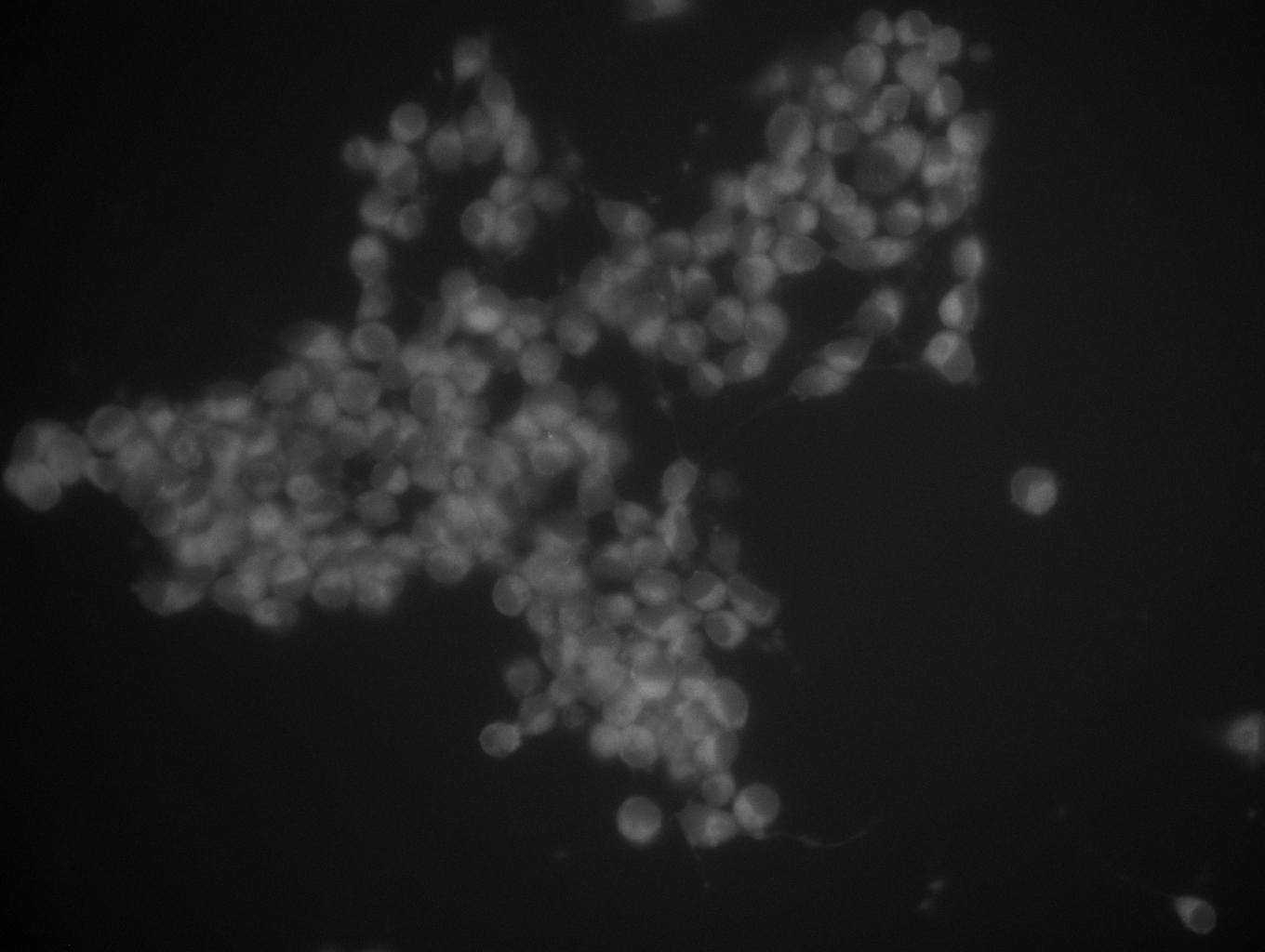

Supplement: Supplementary file 5 — Source data Fig. 4 [file 44321_2025_349_MOESM5_ESM.zip › Source data Figure 4/Fig4E/R2/PIT Representative photos R2/20x/super_FL20240126PIT396.tif]

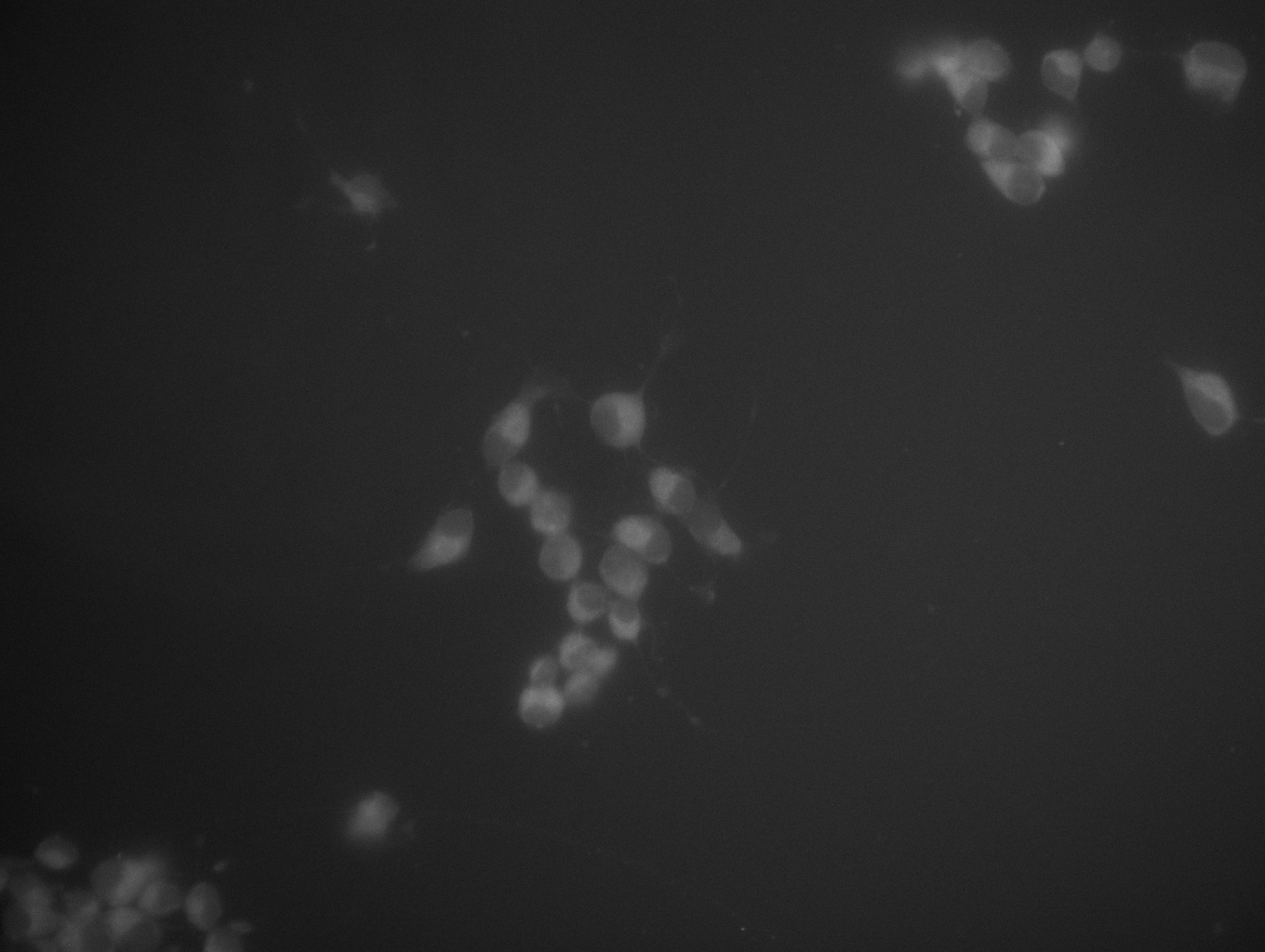

Supplement: Supplementary file 5 — Source data Fig. 4 [file 44321_2025_349_MOESM5_ESM.zip › Source data Figure 4/Fig4E/R2/PIT Representative photos R2/20x/super_FL20240126PIT398.tif]

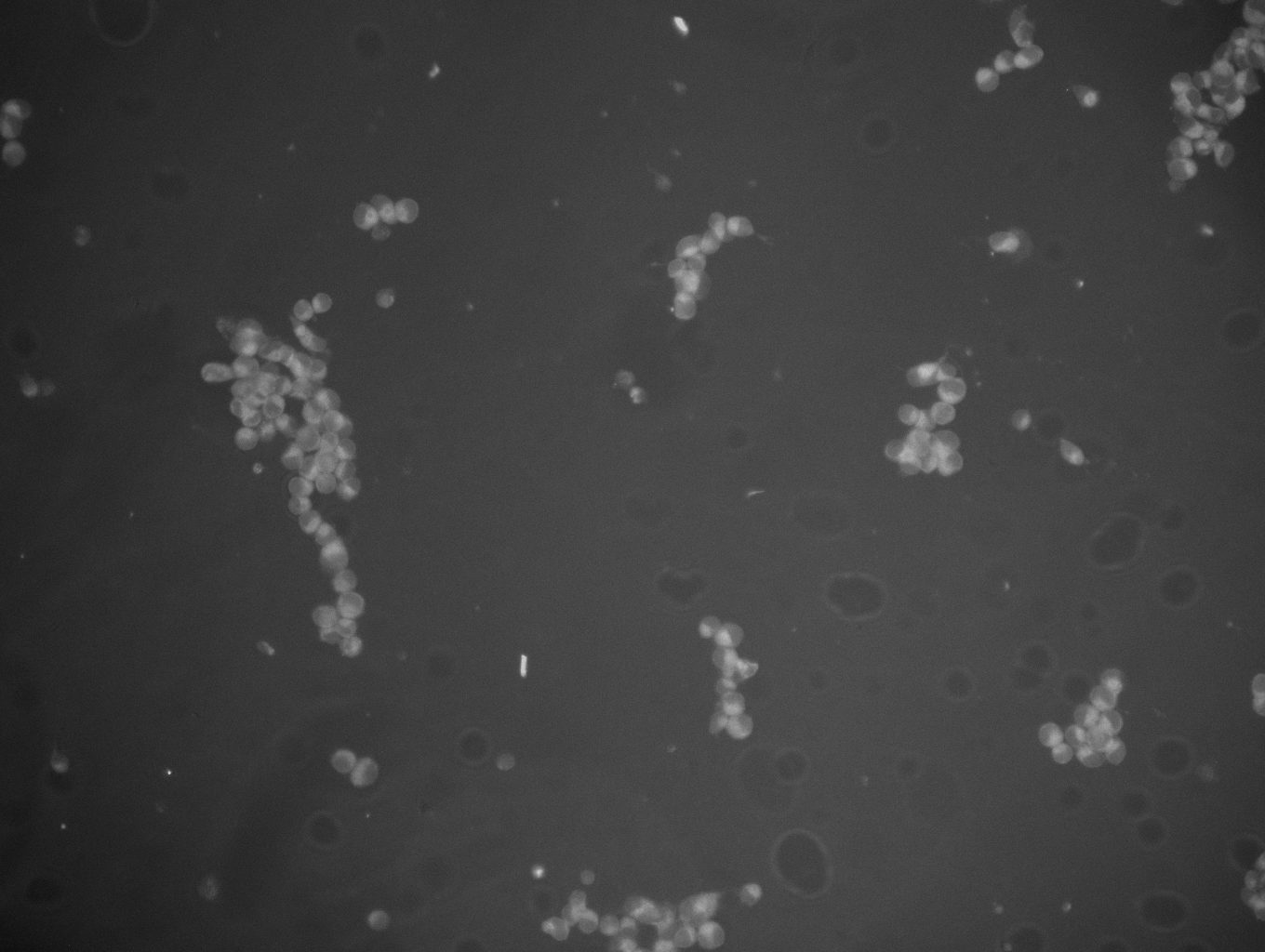

Supplement: Supplementary file 5 — Source data Fig. 4 [file 44321_2025_349_MOESM5_ESM.zip › Source data Figure 4/Fig4E/R2/PITPCZ Representative photos R2/10x/super_FL20240126277PITPCP.tif]

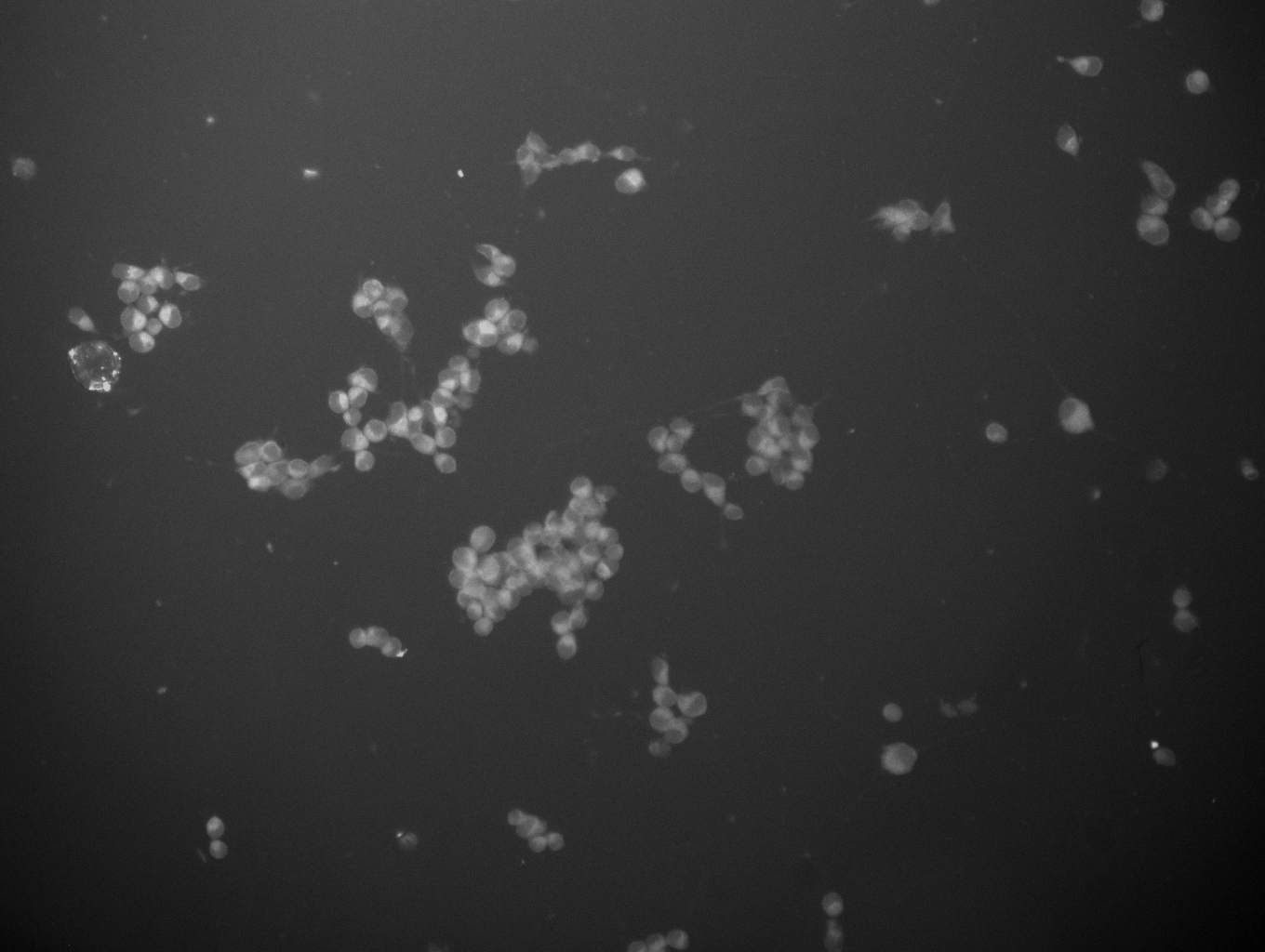

Supplement: Supplementary file 5 — Source data Fig. 4 [file 44321_2025_349_MOESM5_ESM.zip › Source data Figure 4/Fig4E/R2/PITPCZ Representative photos R2/10x/super_FL20240126281PITPCP.tif]

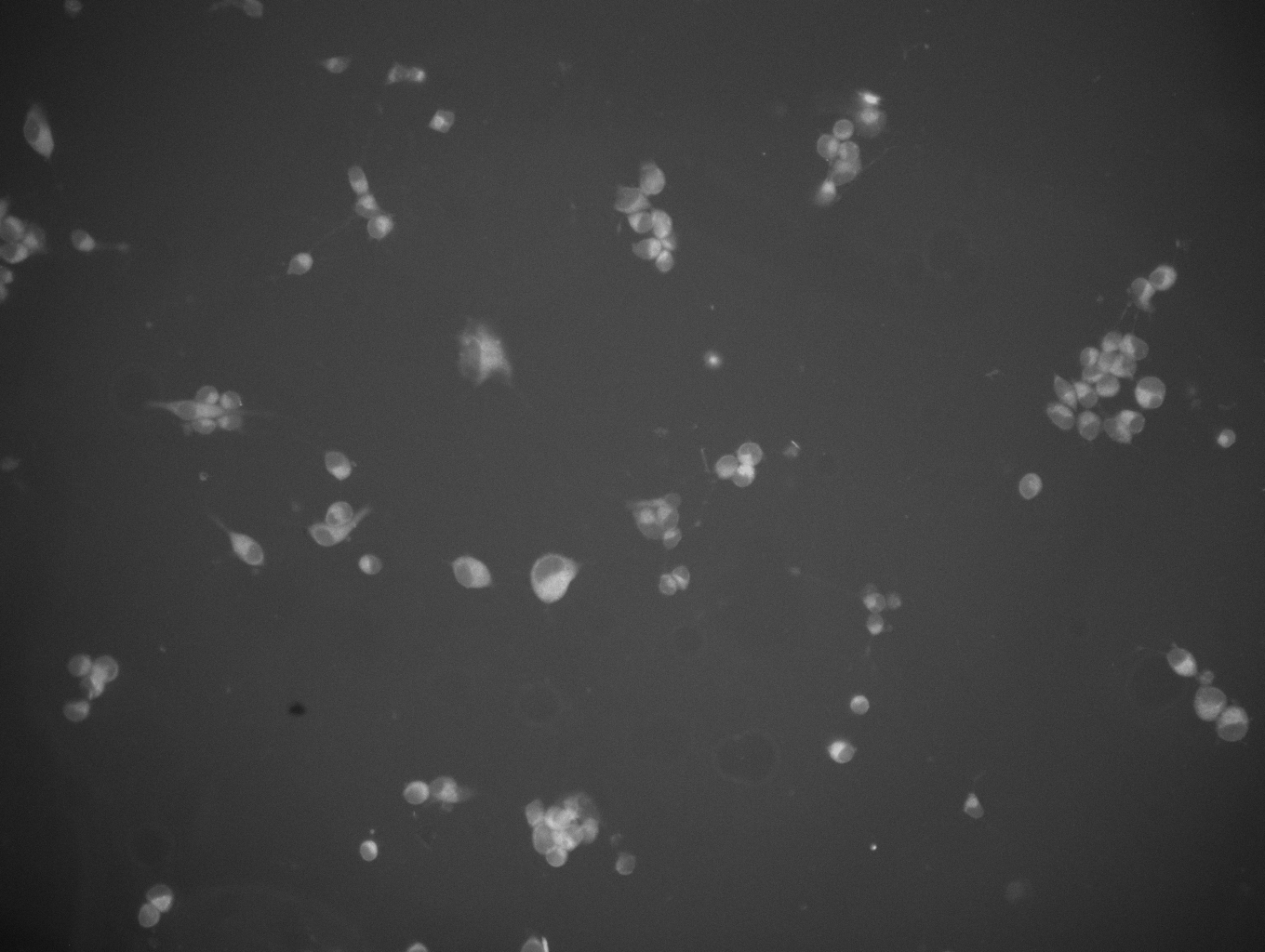

Supplement: Supplementary file 5 — Source data Fig. 4 [file 44321_2025_349_MOESM5_ESM.zip › Source data Figure 4/Fig4E/R2/PITPCZ Representative photos R2/10x/super_FL20240126283PITPCP.tif]

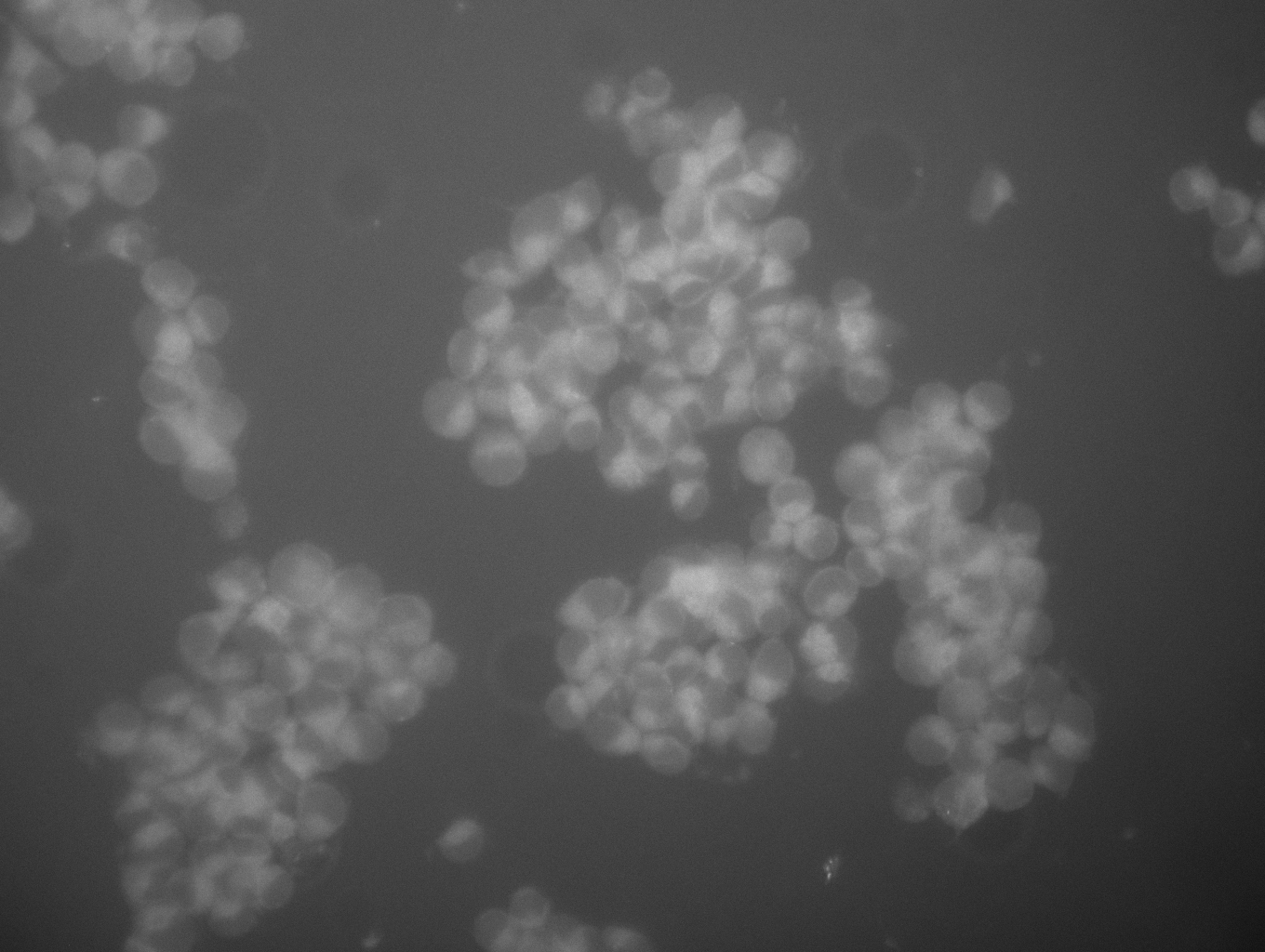

Supplement: Supplementary file 5 — Source data Fig. 4 [file 44321_2025_349_MOESM5_ESM.zip › Source data Figure 4/Fig4E/R2/PITPCZ Representative photos R2/20x/super_FL20240126313PITPCP.tif]

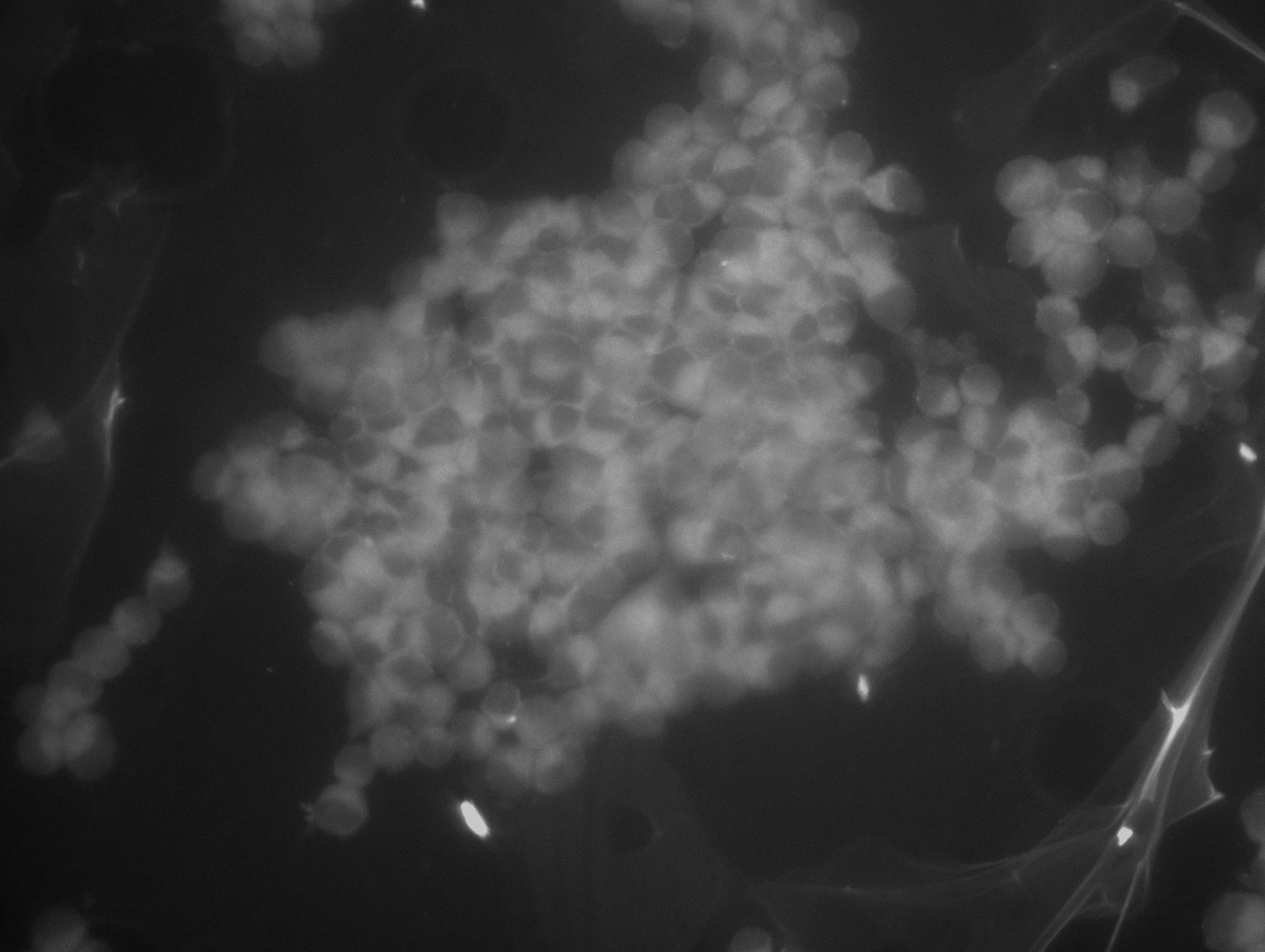

Supplement: Supplementary file 5 — Source data Fig. 4 [file 44321_2025_349_MOESM5_ESM.zip › Source data Figure 4/Fig4E/R2/PITPCZ Representative photos R2/20x/super_FL20240126315PITPCP.tif]

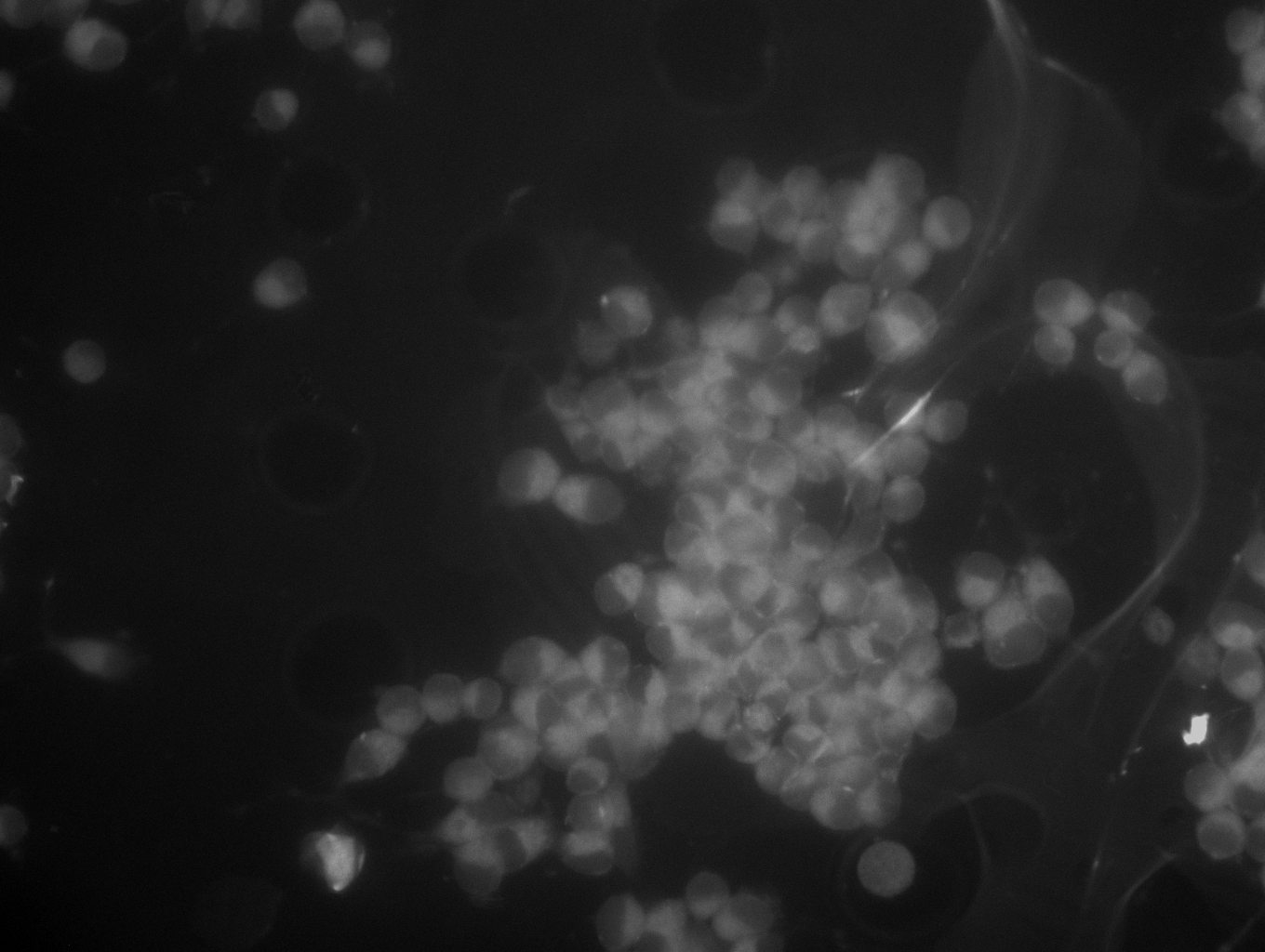

Supplement: Supplementary file 5 — Source data Fig. 4 [file 44321_2025_349_MOESM5_ESM.zip › Source data Figure 4/Fig4E/R2/PITPCZ Representative photos R2/20x/super_FL20240126317PITPCP.tif]

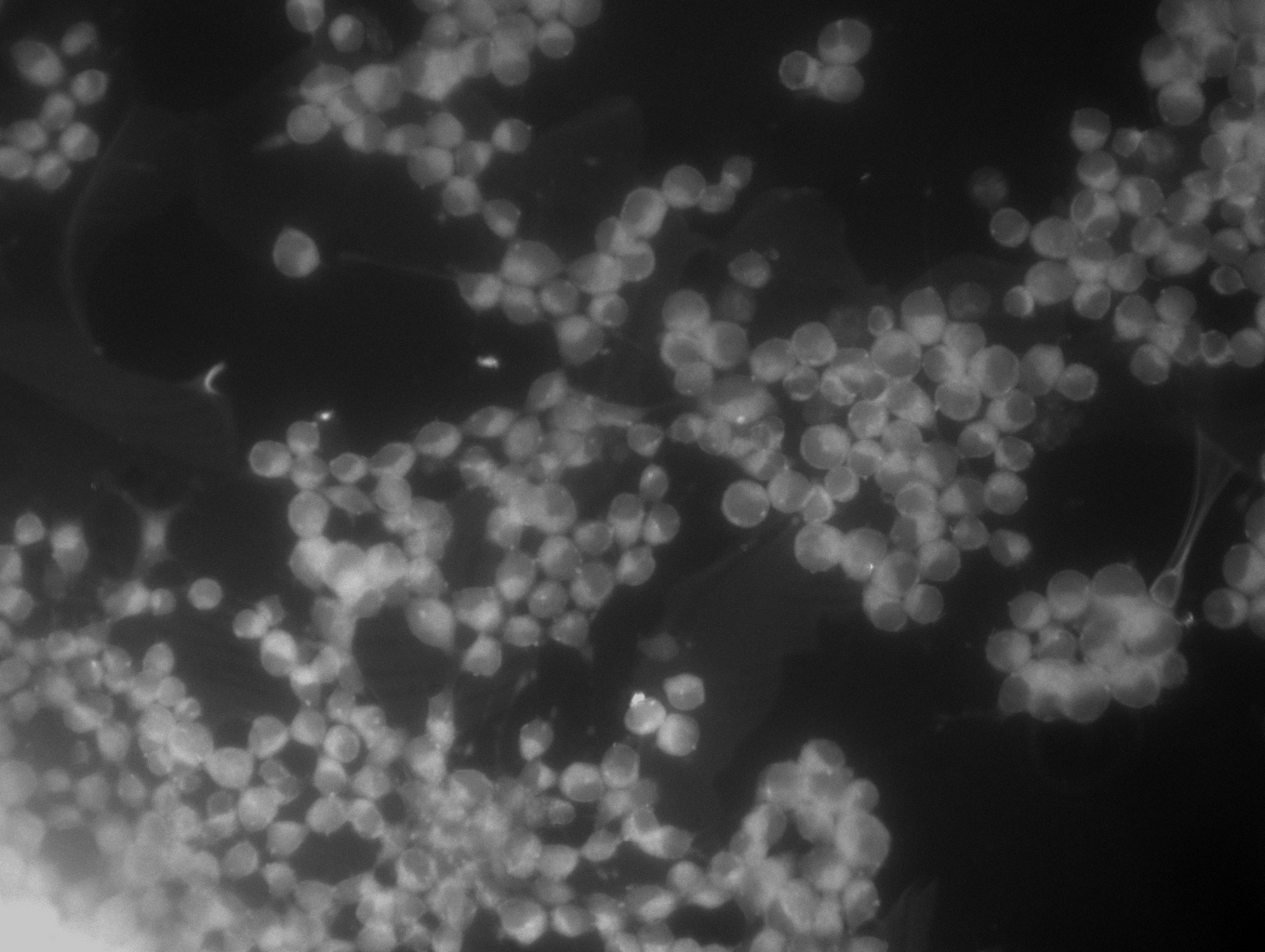

Supplement: Supplementary file 5 — Source data Fig. 4 [file 44321_2025_349_MOESM5_ESM.zip › Source data Figure 4/Fig4E/R2/PITPCZ Representative photos R2/20x/super_FL20240126319PITPCP.tif]

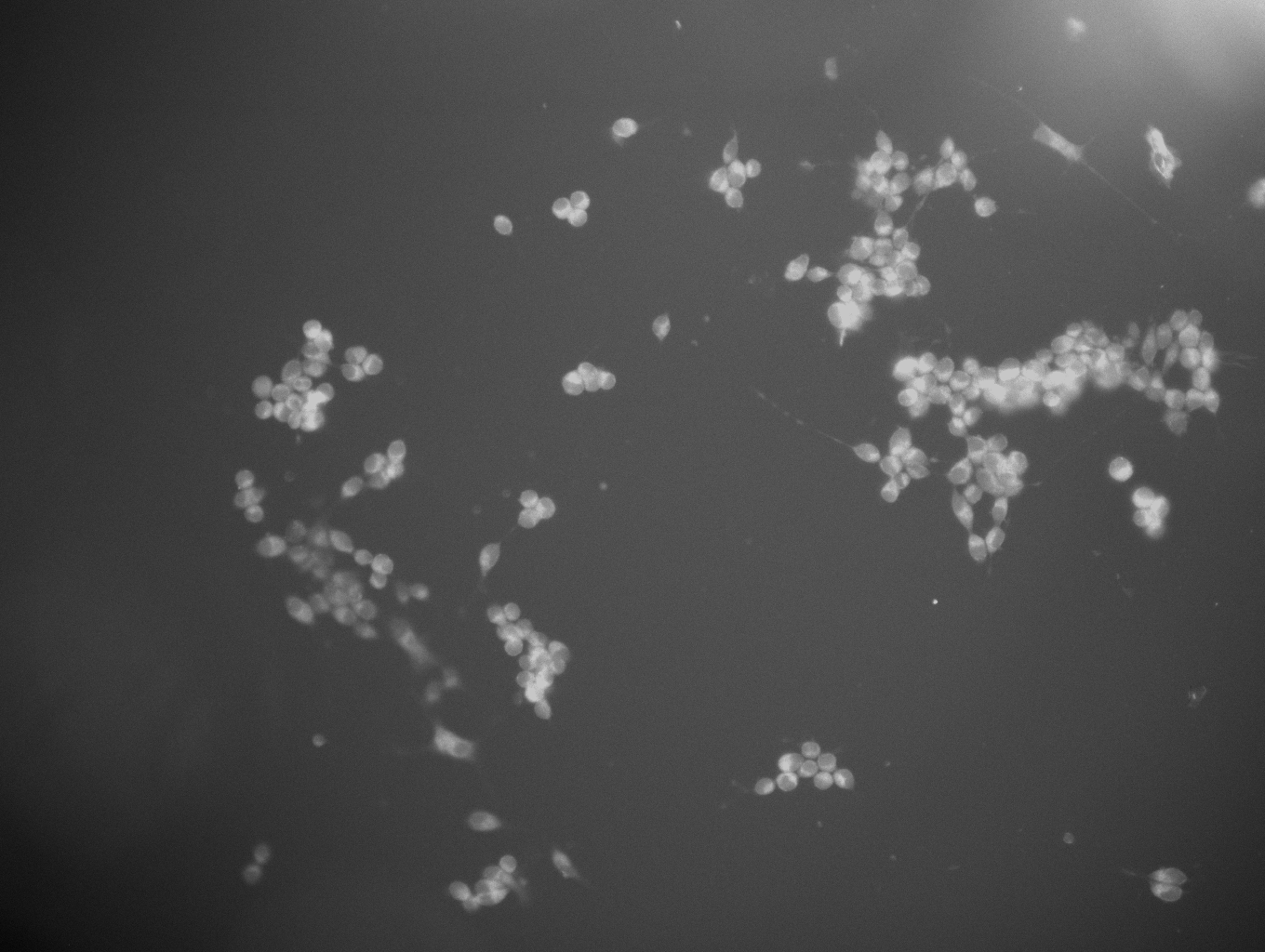

Supplement: Supplementary file 5 — Source data Fig. 4 [file 44321_2025_349_MOESM5_ESM.zip › Source data Figure 4/Fig4E/R2/U18666A_PosCtrl Representative photos R2/10x/super_FL20240126412UA_CTRL.tif]

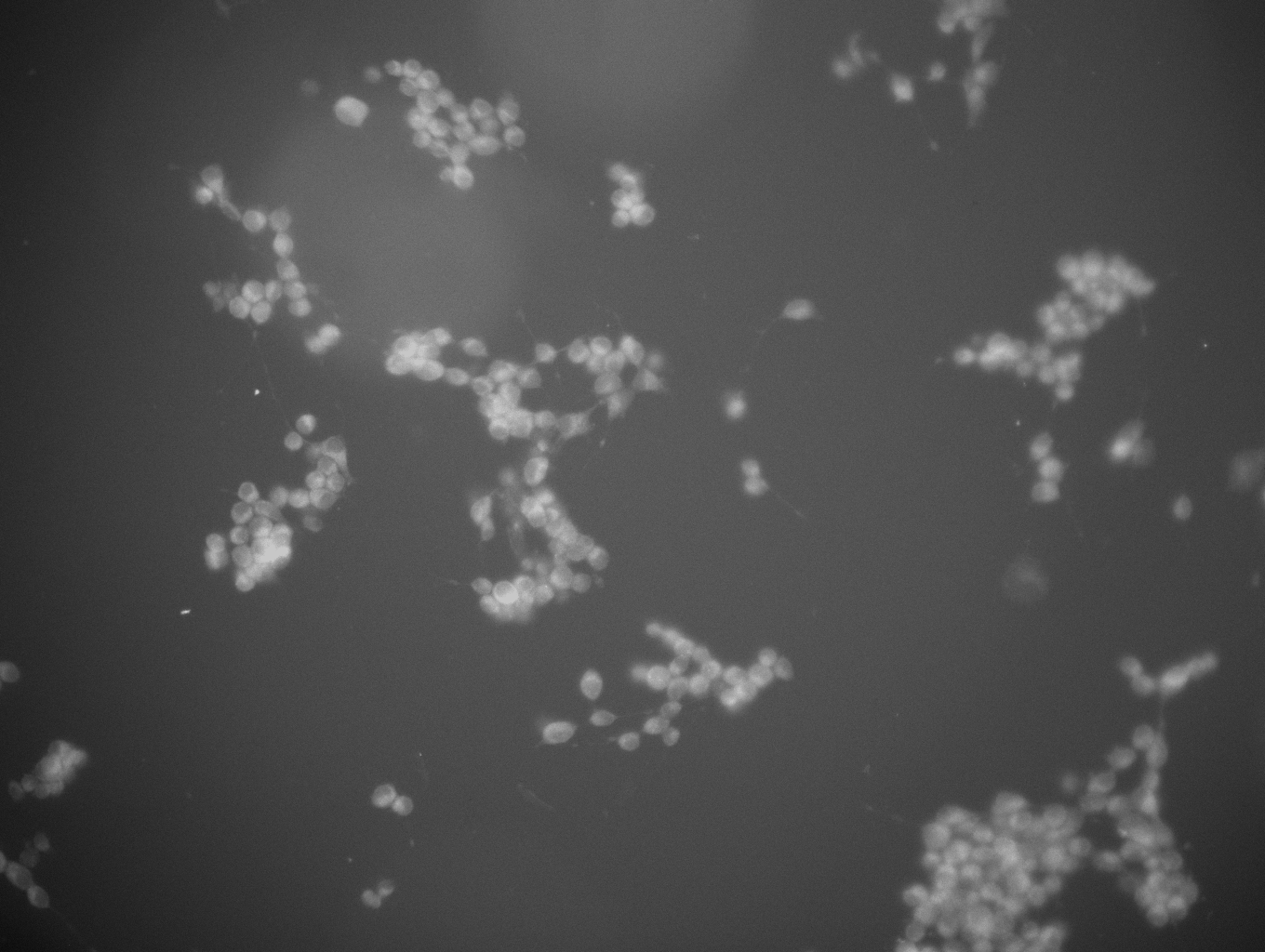

Supplement: Supplementary file 5 — Source data Fig. 4 [file 44321_2025_349_MOESM5_ESM.zip › Source data Figure 4/Fig4E/R2/U18666A_PosCtrl Representative photos R2/10x/super_FL20240126416UA_CTRL.tif]

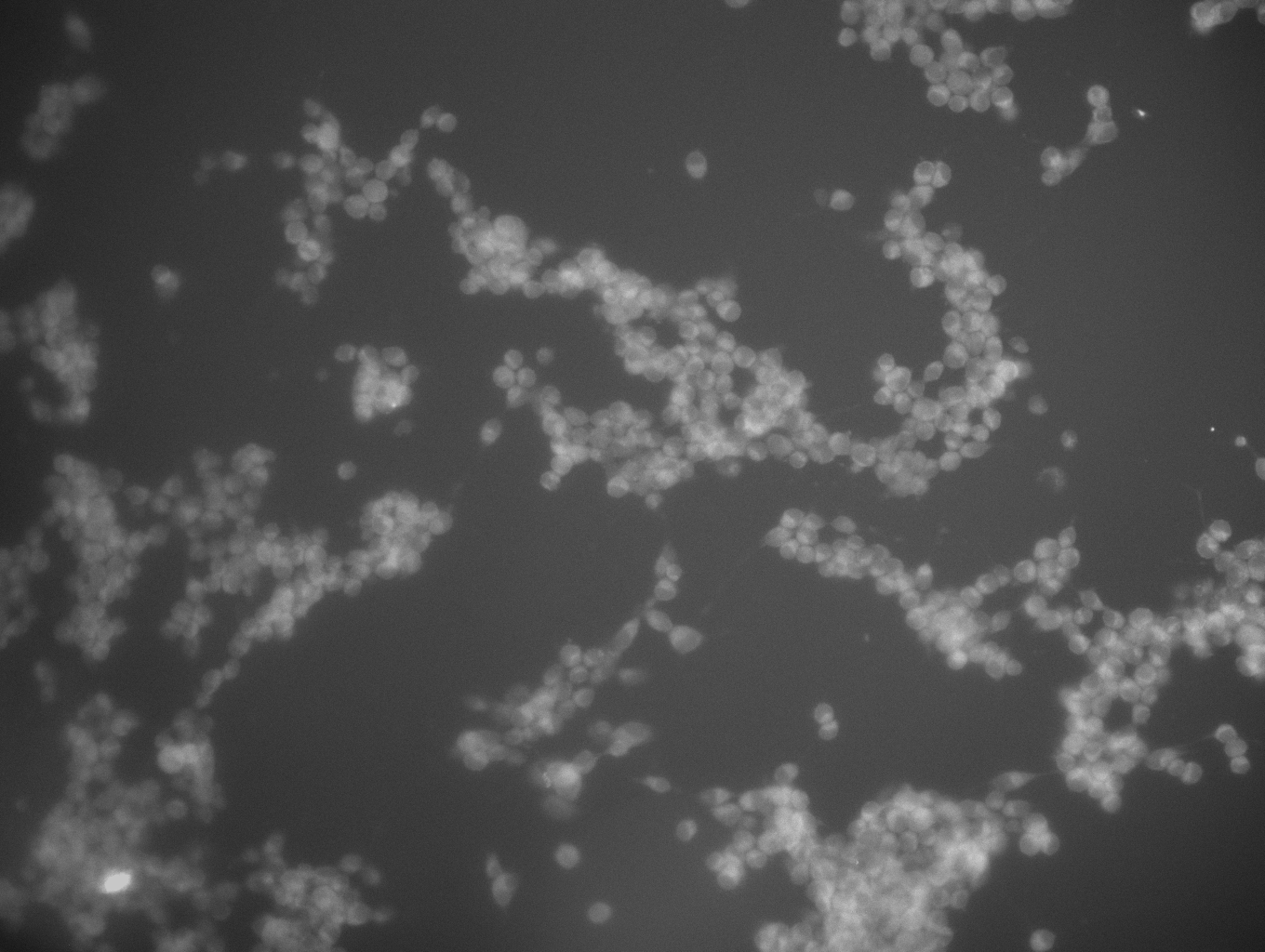

Supplement: Supplementary file 5 — Source data Fig. 4 [file 44321_2025_349_MOESM5_ESM.zip › Source data Figure 4/Fig4E/R2/U18666A_PosCtrl Representative photos R2/10x/super_FL20240126428UA_CTRL.tif]

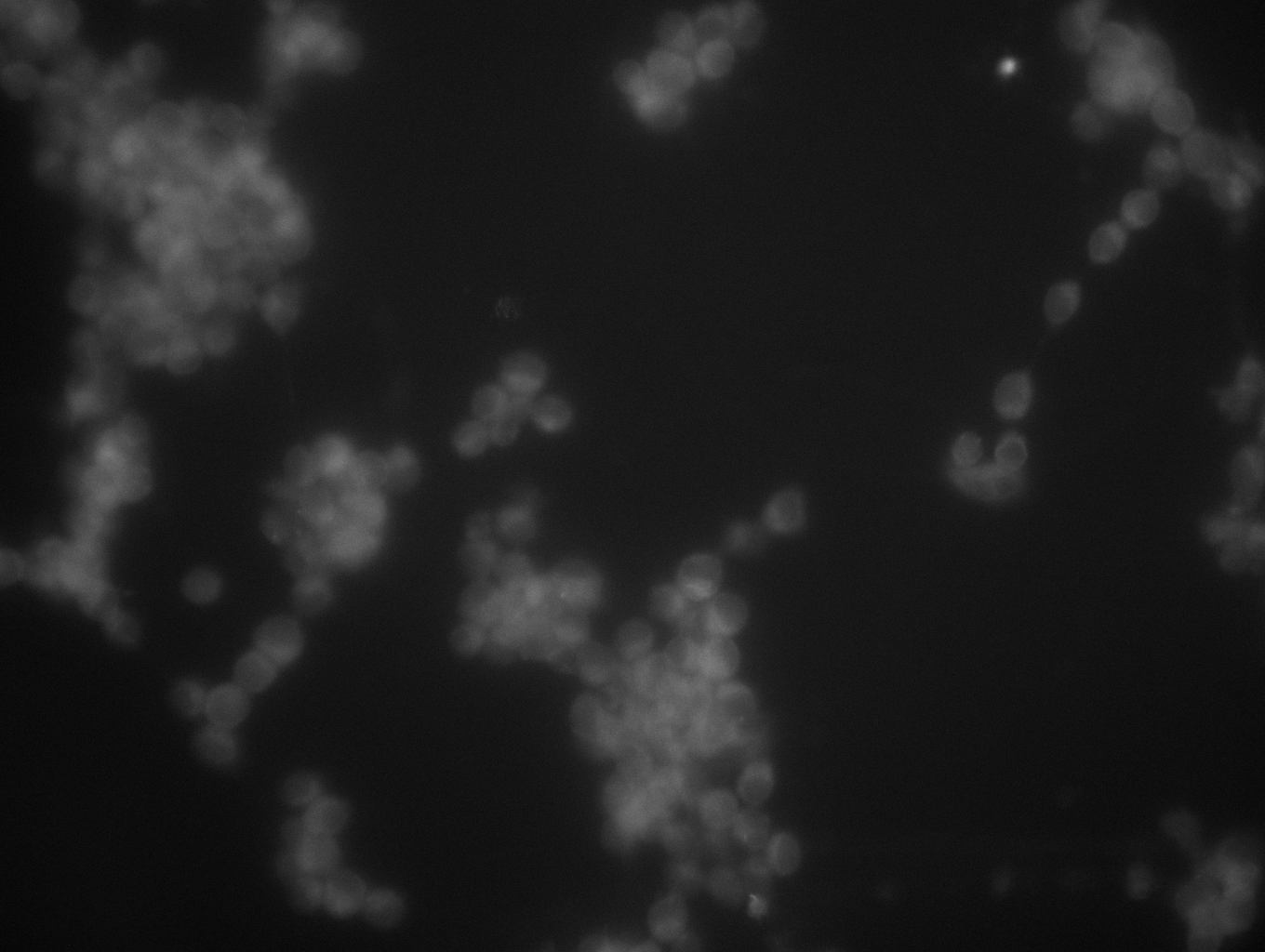

Supplement: Supplementary file 5 — Source data Fig. 4 [file 44321_2025_349_MOESM5_ESM.zip › Source data Figure 4/Fig4E/R2/U18666A_PosCtrl Representative photos R2/20x/super_FL20240126438UA_CTRL.tif]

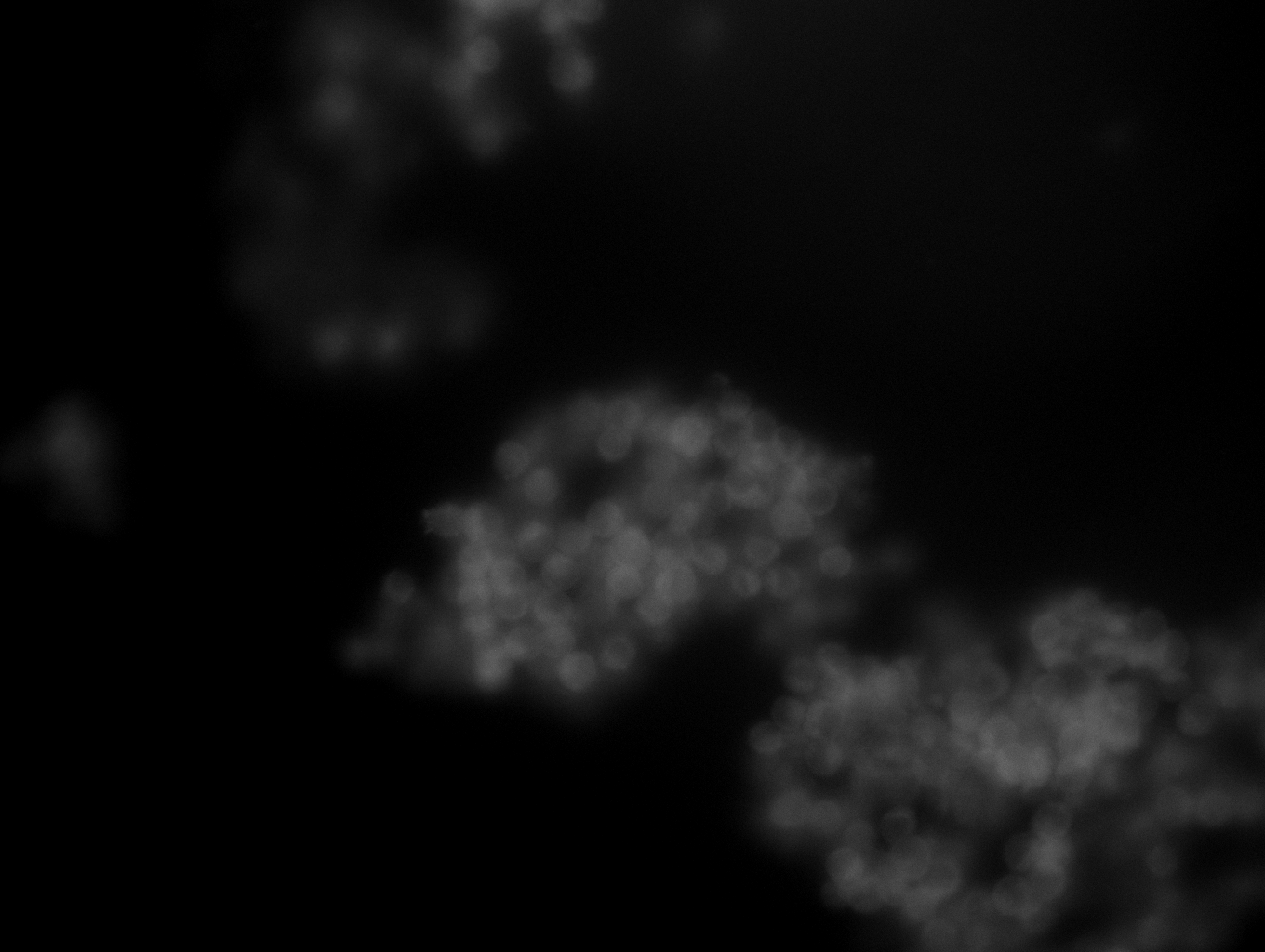

Supplement: Supplementary file 5 — Source data Fig. 4 [file 44321_2025_349_MOESM5_ESM.zip › Source data Figure 4/Fig4E/R2/U18666A_PosCtrl Representative photos R2/20x/super_FL20240126445UA_CTRL.tif]

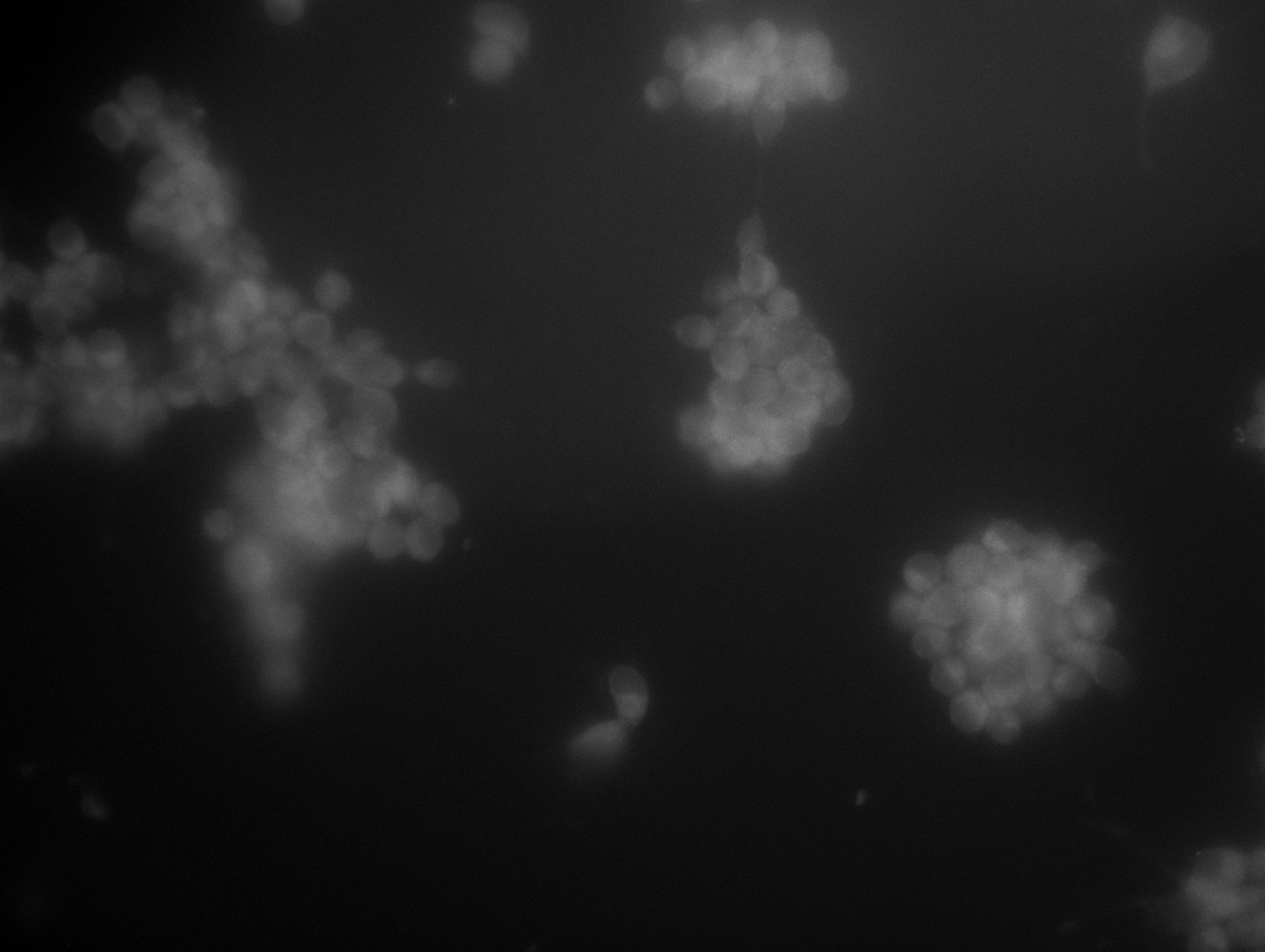

Supplement: Supplementary file 5 — Source data Fig. 4 [file 44321_2025_349_MOESM5_ESM.zip › Source data Figure 4/Fig4E/R2/U18666A_PosCtrl Representative photos R2/20x/super_FL20240126456UA_CTRL.tif]
