# Supplementary material for: Repurposing statins and phenothiazines to treat chemoresistant neuroblastoma
Source: EMBO Mol Med. 2025 Dec 23;18(2):433–61. doi: 10.1038/s44321-025-00349-6 (PMC12905276; doi:10.1038/s44321-025-00349-6)

Uncropped images coresponding to Fig 3D, 3E.
Sample order: Control, PIT, PCZ, Combination. Biological replicates separated by ladders or run on separate blots.

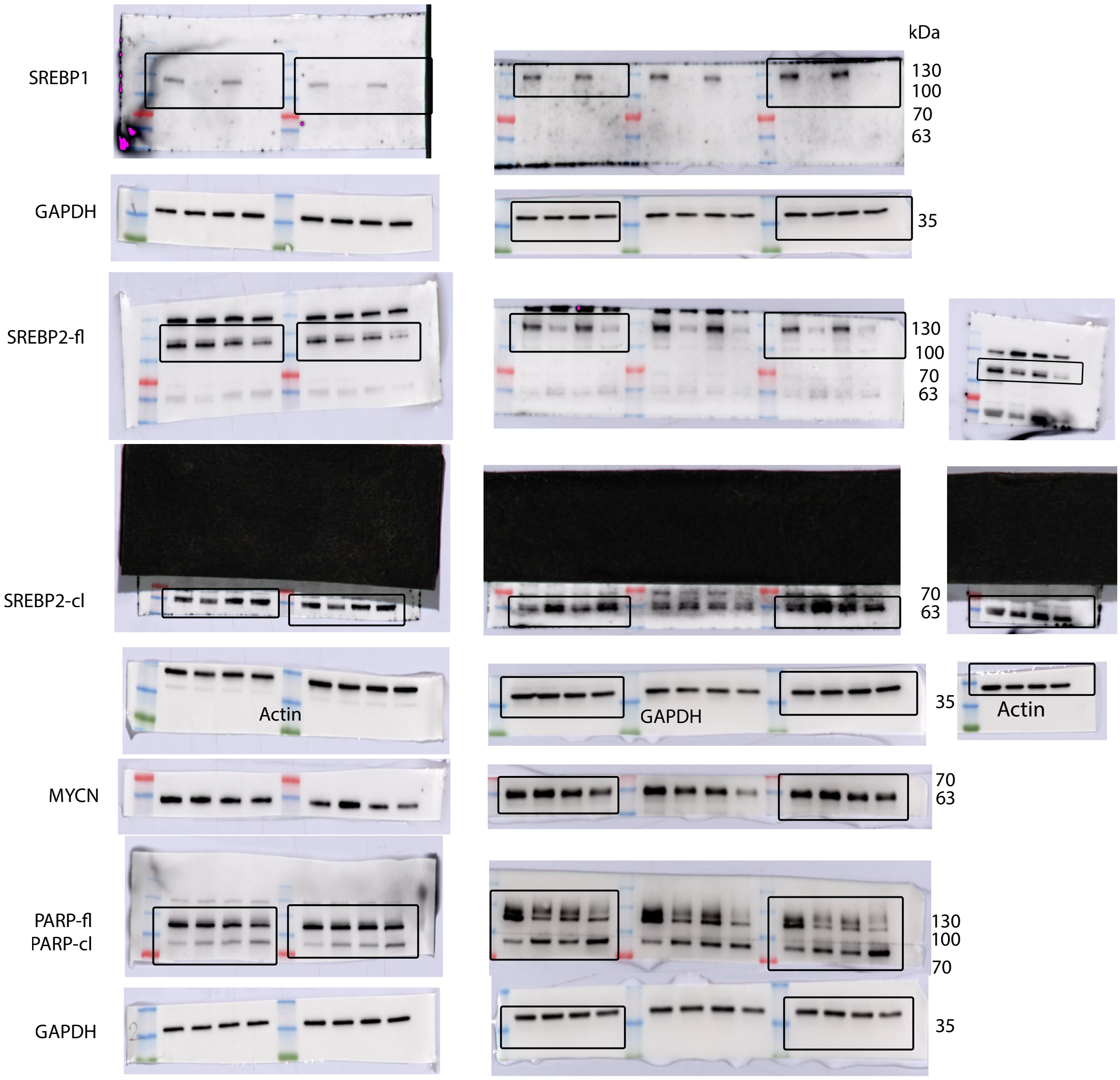

Supplement: Supplementary file 4 — Source data Fig. 3 [file 44321_2025_349_MOESM4_ESM.zip › Source data Figure 3/Fig3D_E/Uncropped WB images 3DE.pdf]
