# Supplementary material for: Repurposing statins and phenothiazines to treat chemoresistant neuroblastoma
Source: EMBO Mol Med. 2025 Dec 23;18(2):433–61. doi: 10.1038/s44321-025-00349-6 (PMC12905276; doi:10.1038/s44321-025-00349-6)

Uncopped images corresponding to Figure 4BC and S3G

Sample order: Control, PIT, PCZ, Combination. Biological replicates separated by ladders or run on separate blots.

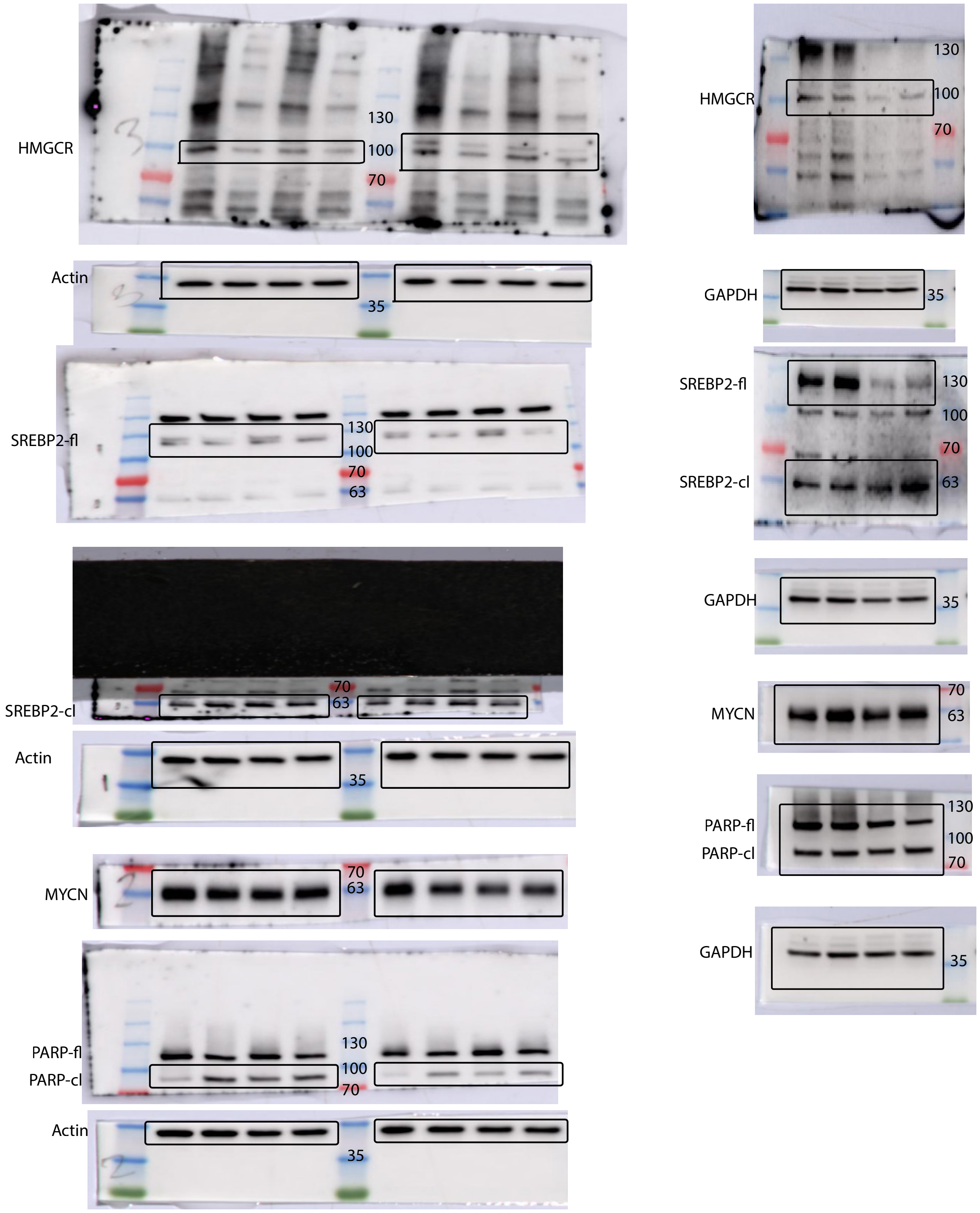

Supplement: Supplementary file 5 — Source data Fig. 4 [file 44321_2025_349_MOESM5_ESM.zip › Source data Figure 4/Fig4BC_S3G/Uncropped WB images siHMGCR experiments.pdf]
