# Supplementary material for: Repurposing statins and phenothiazines to treat chemoresistant neuroblastoma
Source: EMBO Mol Med. 2025 Dec 23;18(2):433–61. doi: 10.1038/s44321-025-00349-6 (PMC12905276; doi:10.1038/s44321-025-00349-6)
Supplement: Supplementary file 11 — Expanded View Figures [file 44321_2025_349_MOESM11_ESM.pdf]

Expanded View Figures

Figure EV1. Single drug efficacy and PCZ + PIT combination synergy.

(**A**) Dose–response curves of 3 or 7 days ($n = 2$). (**B**) Synergy matrix of the PCZ + PIT combination for LU-NB-1 and LU-NB-2 over 3 or 7 days ($n = 2$). (**C**) Response of non-*MYCN* amplified NB cell lines, SK-N-SH and SK-N-AS to Prochlorperazine and Pitavastatin over 3 or 7 days (data represent mean ± SD; $n = 3$). (**D**) Synergy matrix of the PCZ + PIT combination for SK-N-SH and SK-N-AS over 3 or 7 days ($n = 3$). (**E**) Subcutaneous LU-NB-1 PDXs tumor starting volume (control $n = 5$, PCZ $n = 4$, PIT $n = 5$, and combination $n = 6$, one-way ANOVA followed by Tukey's multiple comparisons test, n.s., boxes represent the interquartile range and whiskers indicate minimum and maximum values). (**F**) Average weight ratio per group. (**G**) Tumor size change from baseline for each mouse at day 8 (last day of all mice alive). (**H**) Tumor growth of individual mice over time. PCZ prochlorperazine, TFP trifluoperazine, PIT pitavastatin, i.t. intratumoral injection. Source data are available online for this figure.

 © The Author(s)

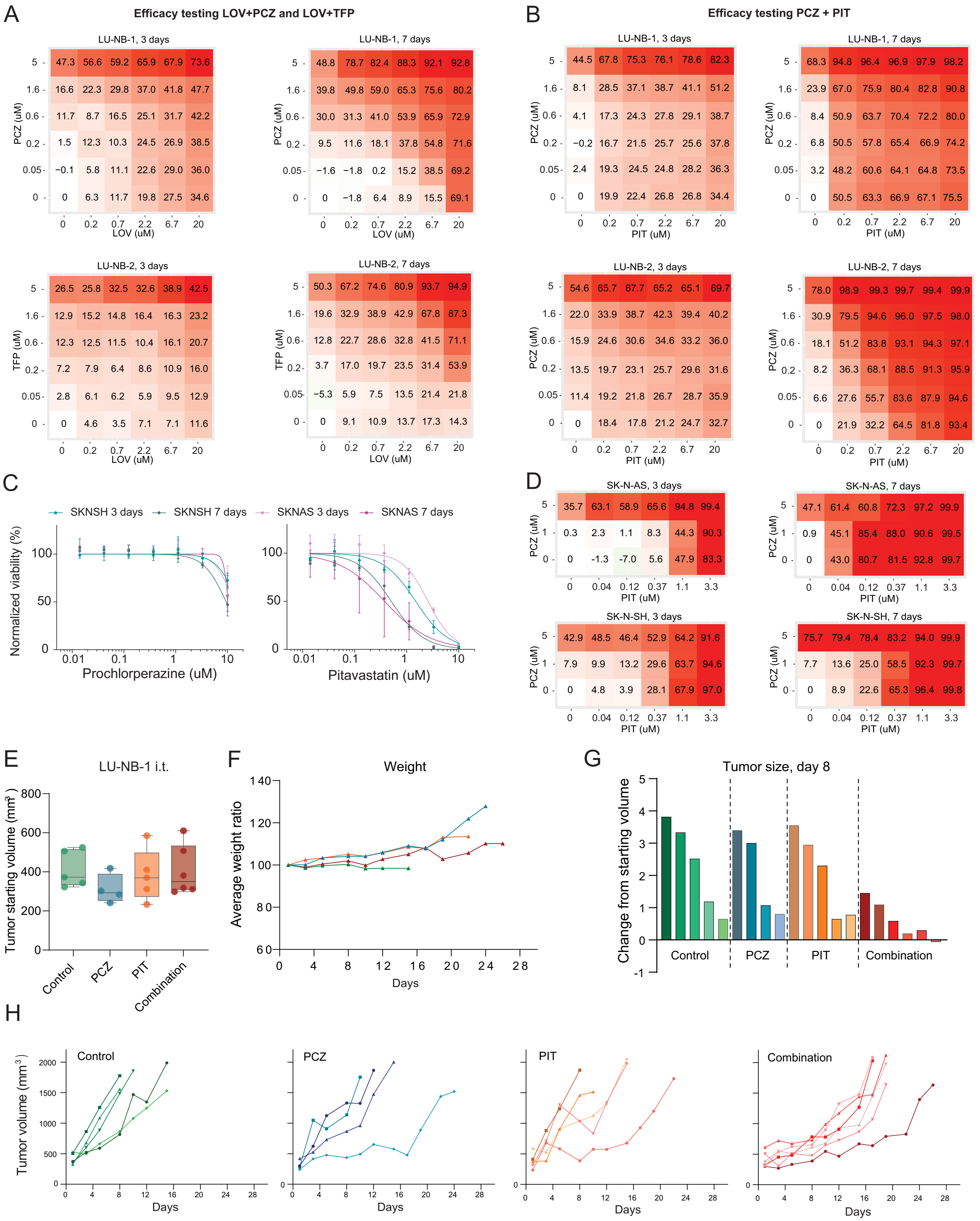
A
Efficacy testing LOV+PCZ and LOV+TFP
LU-NB-1, 3 days
5 47.3 56.6 59.2 65.9 67.9 73.6
1.6 16.6 22.3 29.8 37.0 41.8 47.7
0.6 11.7 8.7 16.5 25.1 31.7 42.2
0.2 1.5 12.3 10.3 24.5 26.9 38.5
0.05 −0.1 5.8 11.1 22.6 29.0 36.0
0 0 6.3 11.7 19.8 27.5 34.6
PCZ (uM)
0 0.2 0.7 2.2 6.7 20
LOV (uM)
LU-NB-1, 7 days
5 48.8 78.7 82.4 88.3 92.1 92.8
1.6 39.8 49.8 59.0 65.3 75.6 80.2
0.6 30.0 31.3 41.0 53.9 65.9 72.9
0.2 9.5 11.6 18.1 37.8 54.8 71.6
0.05 −1.6 −1.8 0.2 15.2 38.5 69.2
0 0 −1.8 6.4 8.9 15.5 69.1
PCZ (uM)
0 0.2 0.7 2.2 6.7 20
LOV (uM)
LU-NB-2, 3 days
5 26.5 25.8 32.5 32.6 38.9 42.5
1.6 12.9 15.2 14.8 16.4 16.3 23.2
0.6 12.3 12.5 11.5 10.4 16.1 20.7
0.2 7.2 7.9 6.4 8.6 10.9 16.0
0.05 2.8 6.1 6.2 5.9 9.5 12.9
0 0 4.6 3.5 7.1 7.1 11.6
TFP (uM)
0 0.2 0.7 2.2 6.7 20
LOV (uM)
LU-NB-2, 7 days
5 50.3 67.2 74.6 80.9 93.7 94.9
1.6 19.6 32.9 38.9 42.9 67.8 87.3
0.6 12.8 22.7 28.6 32.8 41.5 71.1
0.2 3.7 17.0 19.7 23.5 31.4 53.9
0.05 −5.3 5.9 7.5 13.5 21.4 21.8
0 0 9.1 10.9 13.7 17.3 14.3
TFP (uM)
0 0.2 0.7 2.2 6.7 20
LOV (uM)
B
Efficacy testing PCZ + PIT
LU-NB-1, 3 days
5 44.5 67.8 75.3 76.1 78.6 82.3
1.6 8.1 28.5 37.1 38.7 41.1 51.2
0.6 4.1 17.3 24.3 27.8 29.1 38.7
0.2 −0.2 16.7 21.5 25.7 25.6 37.8
0.05 2.4 19.3 24.5 24.8 28.2 36.3
0 0 19.9 22.4 26.8 26.8 34.4
PCZ (uM)
0 0.2 0.7 2.2 6.7 20
PIT (uM)
LU-NB-1, 7 days
5 68.3 94.8 96.4 96.9 97.9 98.2
1.6 23.9 67.0 75.9 80.4 82.8 90.8
0.6 8.4 50.9 63.7 70.4 72.2 80.0
0.2 6.8 50.5 57.8 65.4 66.9 74.2
0.05 3.2 48.2 60.6 64.1 64.8 73.5
0 0 50.5 63.3 66.9 67.1 75.5
PCZ (uM)
0 0.2 0.7 2.2 6.7 20
PIT (uM)
LU-NB-2, 3 days
5 54.6 65.7 67.7 65.2 65.1 69.7
1.6 22.0 33.9 38.7 42.3 39.4 40.2
0.6 15.9 24.6 30.6 34.6 33.2 36.0
0.2 13.5 19.7 23.1 25.7 29.6 31.6
0.05 11.4 19.2 21.8 26.7 28.7 35.9
0 0 18.4 17.8 21.2 24.7 32.7
PCZ (uM)
0 0.2 0.7 2.2 6.7 20
PIT (uM)
LU-NB-2, 7 days
5 78.0 98.9 99.3 99.7 99.4 99.9
1.6 30.9 79.5 94.6 96.0 97.5 98.0
0.6 18.1 51.2 83.8 93.1 94.3 97.1
0.2 8.2 36.3 68.1 88.5 91.3 95.9
0.05 6.6 27.6 55.7 83.6 87.9 94.6
0 0 21.9 32.2 64.5 81.8 93.4
PCZ (uM)
0 0.2 0.7 2.2 6.7 20
PIT (uM)
C
SKNSH 3 days
SKNSH 7 days
SKNAS 3 days
SKNAS 7 days
Normalized viability (%)
100
50
0
0.01 0.1 1 10
Prochlorperazine (uM)
0.01 0.1 1 10
Pitavastatin (uM)
D
SK-N-AS, 3 days
5 35.7 63.1 58.9 65.6 94.8 99.4
1 0.3 2.3 1.1 8.3 44.3 90.3
0 0 -1.3 -7.0 5.6 47.9 83.3
PCZ (uM)
0 0.04 0.12 0.37 1.1 3.3
PIT (uM)
SK-N-AS, 7 days
5 47.1 61.4 60.8 72.3 97.2 99.9
1 0.9 45.1 85.4 88.0 90.6 99.5
0 0 43.0 80.7 81.5 92.8 99.7
PCZ (uM)
0 0.04 0.12 0.37 1.1 3.3
PIT (uM)
SK-N-SH, 3 days
5 42.9 48.5 46.4 52.9 64.2 91.6
1 7.9 9.9 13.2 29.6 63.7 94.6
0 0 4.8 3.9 28.1 67.9 97.0
PCZ (uM)
0 0.04 0.12 0.37 1.1 3.3
PIT (uM)
SK-N-SH, 7 days
5 75.7 79.4 78.4 83.2 94.0 99.9
1 7.7 13.6 25.0 58.5 92.3 99.7
0 0 8.9 22.6 65.3 96.4 99.8
PCZ (uM)
0 0.04 0.12 0.37 1.1 3.3
PIT (uM)
E
LU-NB-1 i.t.
Tumor starting volume (mm³)
800
600
400
200
0
Control
PCZ
PIT
Combination
F
Weight
Average weight ratio
140
120
100
80
60
0 4 8 12 16 20 24 28
Days
G
Tumor size, day 8
Change from starting volume
5
4
3
2
1
0
-1
Control
PCZ
PIT
Combination
H
Tumor volume (mm³)
Control
2000
1500
1000
500
0
0 4 8 12 16 20 24 28
Days
PCZ
0 4 8 12 16 20 24 28
Days
PIT
0 4 8 12 16 20 24 28
Days
Combination
0 4 8 12 16 20 24 28
Days

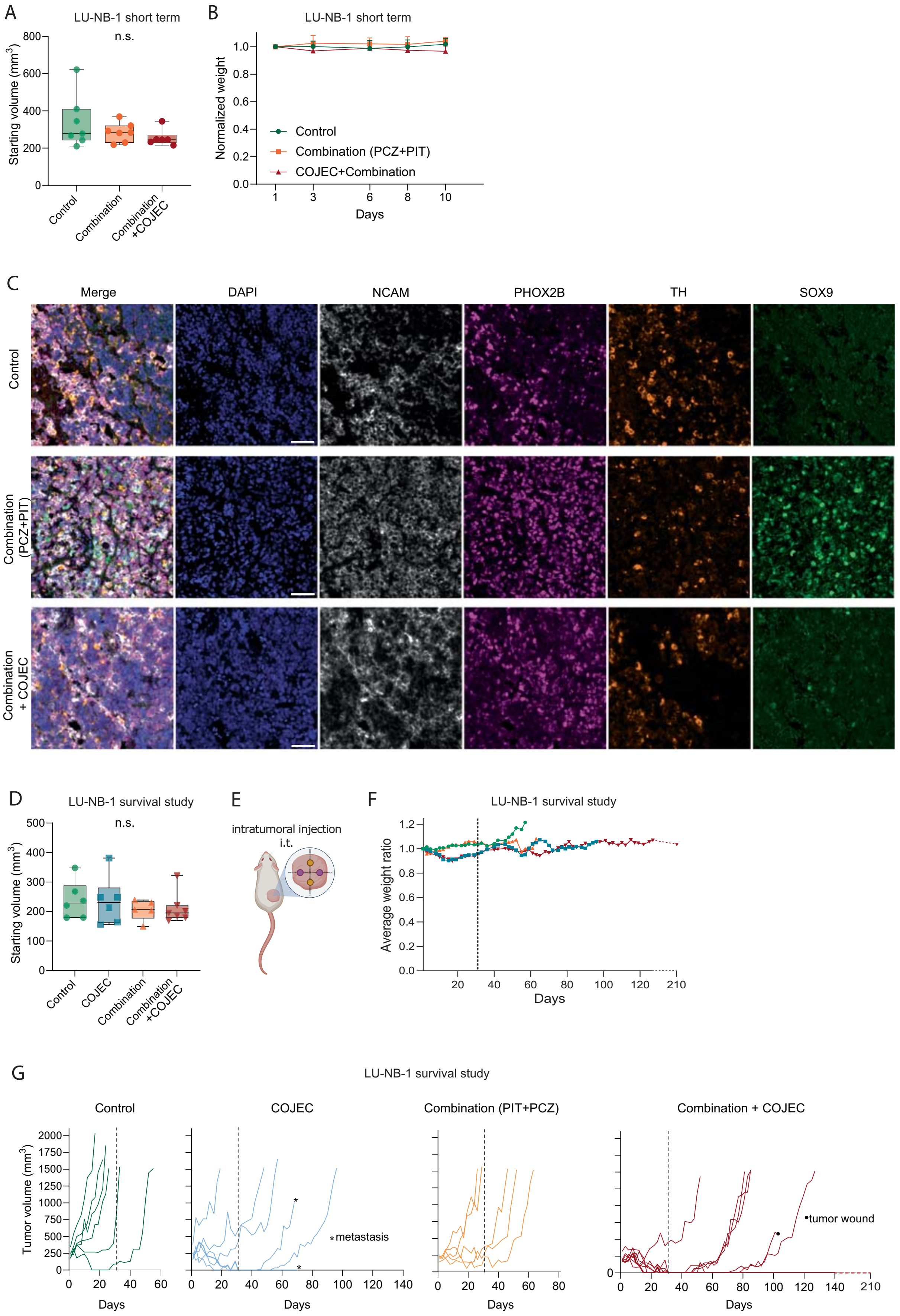
A
LU-NB-1 short term
n.s.
Starting volume (mm3)
Control
Combination
Combination +COJEC
B
LU-NB-1 short term
Normalized weight
Control
Combination (PCZ+PIT)
COJEC+Combination
Days
C
Merge
DAPI
NCAM
PHOX2B
TH
SOX9
Control
Combination (PCZ+PIT)
Combination + COJEC
D
LU-NB-1 survival study
n.s.
Starting volume (mm3)
Control
COJEC
Combination
Combination +COJEC
E
intratumoral injection i.t.
F
LU-NB-1 survival study
Average weight ratio
Days
G
LU-NB-1 survival study
Control
COJEC
Combination (PIT+PCZ)
Combination + COJEC
Tumor volume (mm3)
metastasis
tumor wound
Days

 © The Author(s)

Figure EV2. PCZ + PIT enhances standard-of-care COJEC compared to COJEC alone.

Short-term study. (**A**) LU-NB-1 PDXs tumor starting volume (control $n = 7$, combination $n = 7$, and combination + COJEC $n = 6$, one-way ANOVA followed by Tukey's multiple comparisons test, n.s. boxes represent the interquartile range and whiskers indicate minimum and maximum values). (**B**) Average weight ratio per group (control $n = 7$, combination $n = 7$, and combination + COJEC $n = 6$; data represent mean ± SD). (**C**) Representative images of single marker expression and of co-expression for each treatment group. NCAM = grey, PHOX2B = magenta, TH = orange, SOX9 = green. Scale bar = 50 µm. Survival study. (**D**) LU-NB-1 PDXs tumor starting volume (control $n = 6$, COJEC $n = 6$, combination $n = 5$, comb + COJEC $n = 7$, one-way ANOVA followed by Tukey's multiple comparisons test, n.s. boxes represent the interquartile range and whiskers indicate minimum and maximum values). (**E**) Schematic representation of intratumoral injections. (**F**) Average weight ratio per group throughout the study. Treatment stop marked with dotted line. (**G**) Tumor growth of individual mice over time for each of the groups. Treatment stop marked with dotted line. Combination=PCZ + PIT; COJEC- see Fig. 6G; PCZ prochlorperazine, PIT pitavastatin, i.t. intratumoral. Source data are available online for this figure.

© The Author(s)
