# Supplementary material for: Repurposing statins and phenothiazines to treat chemoresistant neuroblastoma
Source: EMBO Mol Med. 2025 Dec 23;18(2):433–61. doi: 10.1038/s44321-025-00349-6 (PMC12905276; doi:10.1038/s44321-025-00349-6)
Supplement: Supplementary file 4 — Source data Fig. 3 [file 44321_2025_349_MOESM4_ESM.zip › Source data Figure 3/Fig3F/250903 Raw images and specification.zip!/250903 Raw images and specification/Images specifications_CL.docx]

Images used for Fig. 3F

- LU-NB-2 CTRL_SREBP2_DAPI_crop_merge
- LU-NB-2 CTRL_SREBP2_crop
- LU-NB-2 CTRL_DAPI_crop
- LU-NB-2 PIT+PCP_SREBP2_DAPI_crop_merge
- LU-NB-2 PIT+PCP_SREBP2_crop
- LU-NB-2 PIT+PCP_DAPI_crop

Original raw file:

- LU-NB-2 CTRL_SREBP2_raw
- LU-NB-2 CTRL_DAPI_raw
- LU-NB-2 PIT+PCP_SREBP2_raw
- LU-NB-2 PIT+PCP_DAPI_raw

Images for the preparation of Fig. 3F were processed using Fiji ImageJ. Images were cropped to the final size (350 px X 350 px) from original raw file (1360 px X 1024 px). Single channels were merged through the Merge channels function and brightness adjusted maintaining the same parameters for both CTRL and treated samples.
